# Echocardiographic Evidence for Valvular Toxicity of Benfluorex: A Double-Blind Randomised Trial in Patients with Type 2 Diabetes Mellitus

**DOI:** 10.1371/journal.pone.0038273

**Published:** 2012-06-19

**Authors:** Geneviève Derumeaux, Laura Ernande, André Serusclat, Evelyne Servan, Eric Bruckert, Hugues Rousset, Stephen Senn, Luc Van Gaal, Brigitte Picandet, François Gavini, Philippe Moulin, on behalf the REGULATE trial investigators

**Affiliations:** 1 Echocardiography Laboratory, Hôpital Louis Pradel, Lyon, France; 2 Department of Medical Imaging and Endocrinology, Hôpital Louis Pradel, Lyon, France; 3 Department of Endocrinology and Metabolism, Hôpital Pitié Salpêtrière, Bâtiment Husson Mourier, Paris, France; 4 Internal Medicine, Centre Hospitalier, Pierre Benite, France; 5 Department of Statistics, University of Glasgow, Glasgow, United Kingdom; 6 Department of Diabetology, Metabolism & Clinical Nutrition, Faculty of Medicine, University Hospital Antwerp, Edegem-Antwerp, Belgium; 7 Institut de Recherches Internationales Servier, Courbevoie, France; 8 Endocrinology Department, Hôpital Louis Pradel, Hospices Civils de Lyon, University Lyon1, Lyon, France; University of British Columbia, Canada

## Abstract

**Objectives:**

REGULATE trial was designed to compare the efficacy and safety of benfluorex versus pioglitazone in type 2 diabetes mellitus (DM) patients.

**Methods:**

Double-blind, parallel-group, international, randomised, non-inferiority trial. More than half of the 196 participating centres were primary care centres. Patients eligible had type 2 DM uncontrolled on sulfonylurea. 846 were randomised. They received study treatment for 1 year. 423 patients were allocated to benfluorex (150 to 450 mg/day) and 423 were allocated to pioglitazone (30 to 45 mg/day). Primary efficacy criterion was HbA_1c_. Safety assessment included blinded echocardiographic evaluation of cardiac and valvular status.

**Results:**

*At baseline, patients were 59.1±10.5 years old with HbA1c 8.3±0.8%, and DM duration 7.1±6.0 years. During the study, mean HbA1c significantly decreased in both groups (benfluorex:* from 8.30±0.80 to 7.77±1.31 versus pioglitazone: from 8.30±0.80 to 7.45±1.30%). The last HbA1c value was significantly lower with pioglitazone than with benfluorex (p<0.001) and non-inferiority of benfluorex was not confirmed (p = 0.19). Among the 615 patients with assessable paired echocardiography (310 benfluorex, 305 pioglitazone), 314 (51%) had at least one morphological valvular abnormality and 515 (84%) at least one functional valvular abnormality at baseline. Emergent morphological abnormalities occurred in 8 patients with benfluorex versus 4 with pioglitazone (OR 1.99), 95% CI (0.59 to 6.69). Emergent regurgitation (new or increased by one grade or more) occurred more frequently with benfluorex (82 patients, 27%) than with pioglitazone (33 patients, 11%) (OR 2.97), 95% CI (1.91 to 4.63) and were mainly rated grade 1; grade 2 (mild) was detected in 2 patients with benfluorex and 3 with pioglitazone. There was no moderate or severe regurgitation.

**Conclusion:**

After 1 year of exposure, our results show a 2.97 fold increase in the incidence of valvular regurgitation with benfluorex and provide evidence for the valvular toxicity of this drug.

**Trial registration:**

www.controlled-trials.com ISRCTN 27354239. isrctn27354239

## Introduction

Treatment of type 2 diabetes mellitus aims to combine glycaemic control with safety to reduce events related to microvascular and cardiovascular complications. We have previously evaluated the efficacy of benfluorex versus placebo in achieving targets for glycaemic control. [1] Our results suggested that benfluorex might be useful as a second-line antidiabetic drug when metformin is not tolerated or contraindicated. In view of concerns surrounding the cardiovascular safety of antidiabetic treatments such as glitazones and sulfonylureas [2], these findings prompted us to set up the REGULATE (Randomised, double-blind study with comparison of bEnfluorex versus pioGlitazone in combination with sULfonylurea administered orAlly for the Treatment of type 2 diabEtes) trial to compare the efficacy and safety of benfluorex with pioglitazone.

**Figure 1:**
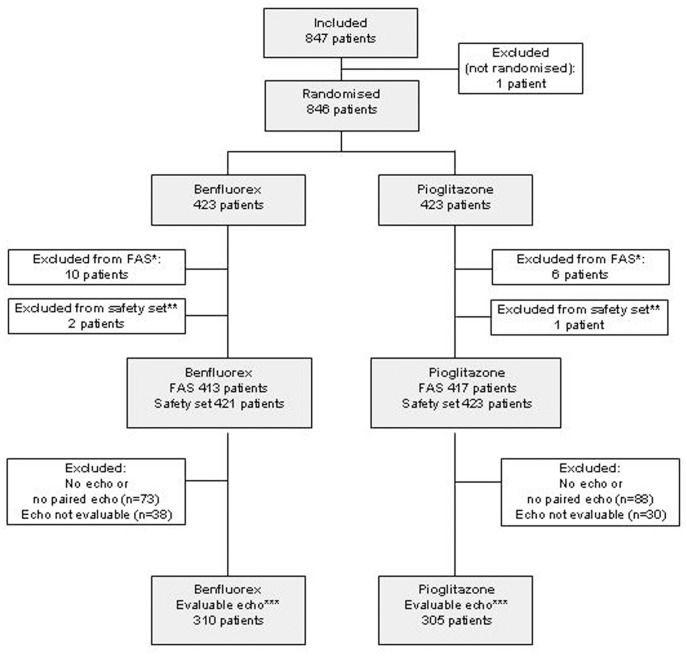
Flow of participants.

At the time of the design of the REGULATE study, a case report had incriminated benfluorex in the development of fibrotic valvular heart disease [3], followed later by similar observations of valvular regurgitation [4].Benfluorex, like fenfluramine, is metabolised into norfenfluramine [5],which is known to activate the serotonin 2B (5-HT_2B_) receptor with subsequent fibroblast proliferation and stimulation of glycosaminoglycan production [6].We therefore decided to use REGULATE to address the issue of valvular safety by performing a complete cardiac examination with echocardiography at baseline and at the end of the study with centralised blind reading. In view of additional case reports of pulmonary hypertension [7],a case-control study [8],a pharmaco-epidemiological study [9],as well as the preliminary results of REGULATE, the French and European authorities decided to withdraw benfluorex from the market 4 years after initiation of the study [10].We report here the complete results of the REGULATE trial, which was designed to compare the efficacy and safety of benfluorex versus pioglitazone over 1 year in type 2 diabetic patients who were not optimally controlled on sulfonylurea monotherapy.

**Table 1:** Main demographic and baseline characteristics in the randomised set.

	All (n = 846)	Benfluorex (n = 423)	Pioglitazone (n = 423)	p-value
Age (years)	59.1±10.5	59.6±10.3	58.6±10.6	0.187
Male	464 (55%)	225 (53%)	239 (57%)	0.369
Ethnic origin				0.140
- Caucasian	646 (76%)	319 (75%)	327 (77%)	
- Asian	157 (19%)	83 (20%)	74 (18%)	
- Other	43 (5%)	21 (5%)	22 (5%)	
Weight (kg)	80.5±14.4	79.7±14.0	81.4±14.8	0.094
Body mass index (kg/m^2^)	29.5±4.0	29.4±4.0	29.7±4.1	0.222
Waist circumference (cm)	101.3±12.1	100.6±11.9	102.1±12.3	0.064
Duration of diabetes (years)	7.1±6.0	7.4±6.0	6.7±5.9	0.098
HbA_1c_ (%)	8.3±0.8	8.3±0.8	8.3±0.8	0.773
Fasting plasma glucose (mmol/L)	9.9±2.6	9.9±2.7	9.8±2.5	0.785
Creatinine clearance <60 mL/min/1.73 m^2^	60 (7%)	36 (9%)	24 (6%)	0.140

Values are numbers (%) or means±SD.

**Figure 2:**
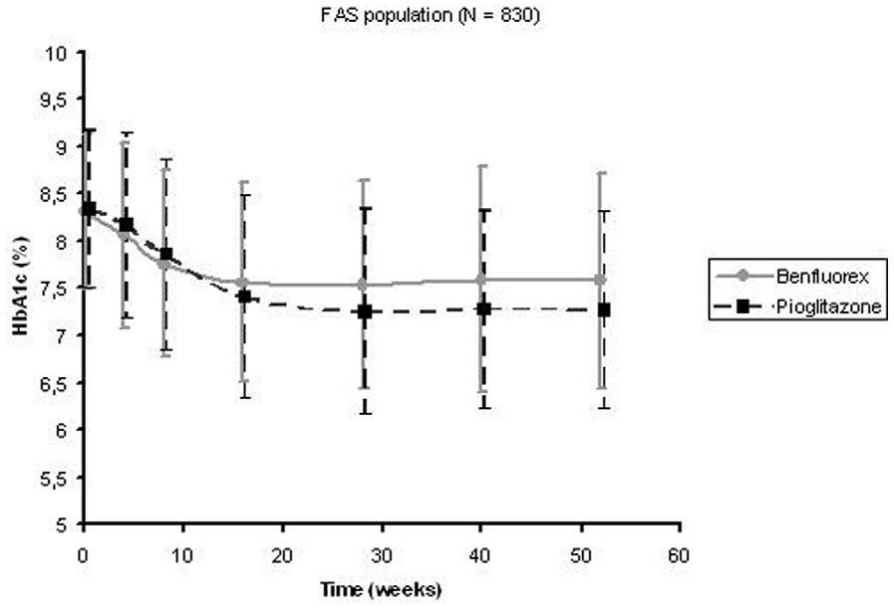
Change in glycosylated haemoglobin (HbA_1c_) in the full analysis set.

## Methods

The protocol for this trial and supporting CONSORT checklist are available as supporting information; see Checklist S1 and Protocol S1.

**Table 2:** Left ventricular (LV) function at baseline and at the end of the study.

Parameter (unit)	Benfluorex	Pioglitazone
**LV ejection fraction (%)**	n = 204	n = 196
- Baseline	69.0±7.2	69.1±7.1
- End	69.4±6.4	69.3±6.5
**LV end diastolic diameter (cm)**	n = 279	n = 272
- Baseline	4.67±0.64	4.70±0.65
- End	4.70±0.63	4.75±0.63
**LV end-systolic diameter (cm)**	n = 279	n = 272
- Baseline	2.71±0.51	2.75±0.54
- End	2.82±0.57	2.82±0.53
**Stroke volume (mL)**	n = 282	n = 253
- Baseline	81.9±44.7	82.5±44.4
- End	75.7±24.3	78.3±24.0
**Cardiac index (L/m/m^2^)**	n = 223	n = 208
- Baseline	3.03±0.84	2.99±1.42
- End	2.79±0.83	2.90±0.84
**Mitral E wave (cm/s)**	n = 325	n = 314
- Baseline	73.5±17.1	73.6±19.6
- End	72.9±18.4	78.5±22.0
**A wave (cm/s)**	n = 324	n = 311
- Baseline	82.4±20.0	82.9±23.1
- End	84.4±21.5	84.1±22.0
**Mitral E/A ratio**	n = 316	n = 309
- Baseline	0.93±0.29	0.94±0.33
- End	0.90±0.26	0.97±0.32
**Mitral E wave deceleration time (s)**	n = 310	n = 290
- Baseline	238.6±61.5	245.8±72.2
- End	246.3±74.8	236.5±65.6
**NTproBNP values (pg/mL)**	n = 341	n = 359
- Baseline	45.0 [26.0–95.0]	49.0 [25.0–94.0]
- End	63.0 [34.0–133.0]	70.0 [31.0–135.0]

Values are numbers (%) or means±SD, except for NT-proBNP, which are median [Q1;Q3]). n = number of assessable patients.

**Table 3:** Baseline morphological and functional valvular abnormalities.

	Benfluorex (n = 310)	Pioglitazone (n = 305)	All (n = 615)
**Morphological abnormalities**	n = 309	n = 305	n = 614
At least one abnormality on any valve	154 (50%)	160 (53%)	314 (51%)
**Aortic valve**	n = 306	n = 302	n = 608
At least one abnormality	101 (33%)	100 (33%)	201 (33%)
Thickness	95 (31%)	94 (31%)	189 (31%)
Calcification	33 (11%)	25 (8%)	58 (10%)
**Mitral valve**	n = 307	n = 300	n = 607
At least one abnormality	125 (41%)	127 (42%)	252 (42%)
Thickness	114 (37%)	120 (40%)	234 (39%)
Calcification	26 (9%)	20 (7%)	46 (8%)
**Tricuspid valve**	n = 302	n = 300	n = 602
At least one abnormality	10 (3%)	11 (4%)	21 (4%)
Thickness	10 (3%)	11 (4%)	21 (4%)
Calcification	–	–	–
**Functional abnormalities**	n = 309	n = 303	n = 612
At least one abnormality on any valve	260 (84%)	255 (84%)	515 (84%)
**Aortic valve**	n = 309	n = 302	611
At least one abnormality	53 (17%)	48 (16%)	101 (17%)
Regurgitation grade 1	49 (16%)	42 (14%)	91 (15%)
grade 2	–	4 (1%)	4 (0.7%)
Stenosis	7 (2%)	4 (1%)	11 (2%)
**Mitral valve**	n = 309	n = 300	n = 609
At least one abnormality	191 (62%)	180 (60%)	371 (61%)
Regurgitation Grade 1	186 (60%)	175 (58%)	361 (59%)
Grade 2	3 (1%)	5 (2%)	8 (1%)
Grade 3	1 (0.3%)	–	1 (0.2%)
Stenosis	1 (0.3%)	2 (0.7%)	3 (0.5%)
**Tricuspid valve**	n = 304	n = 299	n = 603
At least one abnormality	208 (68%)	228 (76%)	436 (72%)
Regurgitation Grade 1	208 (68%)	225 (75%)	433 (72%)
Grade 2	–	3 (1%)	3 (0.5%)
Stenosis	–	–	–

Values are numbers (%). n = number of assessable patients.

### Ethics Considerations

The trial designed in 2005 was conducted in accordance with the ethical principles stated in the Declaration of Helsinki 1964, as revised in Edinburgh, UK, October 2000. The protocol was finally approved by each institution’s ethics committee (listing provided in annexe) and LYON Sud Est II ethical committee (7^th^ of December 2005). All subjects provided written informed consent. The trial is registered on www.controlled-trials.com (ISRCTN 27354239).

**Table 4:** Emergent valvular functional abnormality.

Emergent valvular abnormality	Change in grade frombaseline to last value	Benfluorex(n = 310)	Pioglitazone(n = 305)	p value	Odds Ratio(95%CI)
**Aortic regurgitation**		n = 309	n = 302		
	Total	42 (14%)	3 (1%)	p<0.0001	15.52 [4.76;50.66]
	Grade 0 to 1	40 (13%)	3 (1%)		
	Grade 0 to 2	1 (0.3%)	–		
	Grade 1 to 2	1 (0.3%)	–		
**Mitral regurgitation**		n = 309	n = 300		
	Total	22 (7%)	14 (5%)	p = 0.19	1.58 [0.79;3.16]
	Grade 0 to 1	21 (7%)	13 (4%)		
	Grade 1 to 2	1 (0.3%)	1 (0.3%)		
**Tricuspid regurgitation**		n = 304	n = 299		
	Total	33 (11%)	17 (6%)	p = 0.024	2.01 [1.10;3.70]
	Grade 0 to 1	33 (11%)	15 (5%)		
	Grade 1 to 2	–	2 (0.7%)		
**At least two valvular emergent** **regurgitations**		16 (5%)	1 (0.3%)	P = 0.007	16.43 [2.124]
**3 valvular emergent regurgitations**		4 (1%)	–		

Values are numbers (%). n = number of assessable patients.

### Study Design and Patients

This randomised, double-blind, double-dummy, parallel-group, phase III study (196 centres in 8 countries) compared the efficacy and safety of 1 year’s treatment with benfluorex versus pioglitazone in patients with type 2 diabetes mellitus stratified according to baseline glycosylated haemoglobin (HbA_1c_) (≤8% or >8%) and country. Treatment was allocated under the responsibility of the CRO (S-Clinica, Brussels, Belgium) using a non-adaptative, centralised, balanced (one to one), stratified randomisation (interactive voice response system IVRS designed by the sponsor) according to country and HbA1C (≤8% or >8%). Each investigator received a personal pass-word from the IVRS system for treatment attribution. Both patients and investigators were blinded to treatment allocation. Because of metformin intolerance, all patients were treated by sulfonylurea at their usual dose that was maintained throughout the study, except in case of severe or repeated hypoglycaemia. The trial design involved a 4-week placebo run-in, followed by a 4-month uptitration period and an 8-month dose adaptation period; the aim was to reach the maximal dose tolerated by the patient according to HbA_1c_ (target level <6.5%) and tolerability. The daily dosage of oral benfluorex (150 to 450 mg) and pioglitazone (30 to 45 mg) was uptitrated according to fasting plasma glucose (FPG) levels (target 7.8 mmol/L or lower). The dosage could be reduced at any time according to safety (risk for hypoglycaemia).

**Figure 3:**
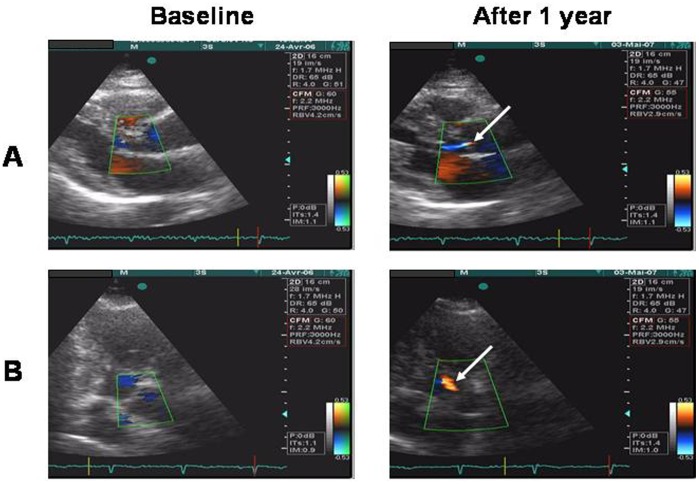
Example of an emergent grade 1 aortic regurgitation in a patient treated with benfluorex. In the parasternal views (row A) and the apical 3-chamber views (row B), grade 1 aortic regurgitation is shown by colour Doppler (arrow) after 1 year of treatment with benfluorex.

**Table 5:** Emergent adverse events leading to treatment withdrawal (in more than one patient in any group) by system organ class and preferred terms in the safety set (N = 844).

System organ class	Benfluorex (n = 421)		Pioglitazone (n = 423)	
	No ofevents	No (%) ofpatients	No ofevents	No (%) ofpatients
**All**	43	40 (10%)	37	35 (8%)
**Cardiac disorders**	7	7 (2%)	7	7 (2%)
Cardiac failure	2	2 (0.5%)	3	3 (0.7%)
Myocardial infarction	2	2 (0.5%)	2	2 (0.5%)
**Gastrointestinal disorders**	11	9 (2%)	2	2 (0.5%)
Diarrhoea	3	3 (0.7%)	–	–
Gastrointestinal disorder	2	2 (0.5%)	–	–
**Nervous system disorders**	5	5 (1%)	3	3 (0.7%)
**General disorders and administration site conditions**	2	2 (0.5%)	5	5 (1%)
Oedema	–	–	3	3 (0.7%)
Asthenia	2	2 (0.5%)	–	–
Oedema peripheral	–	–	2	2 (0.5%)
**Investigations**	2	2 (0.5%)	4	4 (0.9%)
Weight increase	–	–	3	3 (0.7%)
Hepatic enzyme increase	2	2 (0.5%)	–	–
**Neoplasms benign, malignant and unspecified (including cysts and polyps)**	3	3 (0.7%)	2	2 (0.5%)
**Blood and lymphatic system disorders**	2	2 (0.5%)	2	2 (0.5%)
Anaemia	2	2 (0.5%)	2	2 (0.5%)
**Infections and infestations**	3	3 (0.7%)	1	1 (0.2%)
**Respiratory, thoracic, and mediastinal disorders**	1	1 (0.2%)	2	2 (0.5%)
**Injury, poisoning, and procedural complications**	2	2 (0.5%)	–	–
Fall	2	2 (0.5%)	–	–
**Metabolism and nutrition disorders**	–	–	2	2 (0.5%)
**Musculoskeletal and connective tissue disorders**	2	2 (0.5%)	–	–
**Pregnancy, puerperium and perinatal conditions**	–	–	2	2 (0.5%)
Pregnancy	–	–	2	2 (0.5%)

Eligible patients were outpatients with type 2 diabetes mellitus aged between 35 and 80 years with stable bodyweight (body mass index [BMI] between 25 and 40 kg/m^2^ inclusive, except in India where BMI could range from 23 to 40 kg/m^2^) and HbA_1c_ between 7% and 10%. Patients had received a sulfonylurea at a stable dose for at least 3 consecutive months prior to the selection visit (at least 50% of the maximal recommended dose). Clinical heart failure, left ventricular (LV) ejection fraction less than 40%, or any significant valvular heart disease on echocardiography were exclusion criteria.

The pre-specified primary endpoint was HbA_1c_ level. The pre-specified secondary endpoints were low-density lipoprotein (LDL) cholesterol, FPG, waist circumference, and bodyweight, as well as safety criteria including echographic assessment of LV function and valvular status. HbA_1c_ was assessed at every visit (baseline, 1, 2, 4 7, 9, and 12 months) using NGSP-certified high performance liquid chromatography (HPLC) (Biorad Variant I). HbA_1c_, FPG, and standard biochemical, haematological, and lipid parameters, high sensitivity C-reactive protein (hsCRP), and N-terminal pro-brain natriuretic peptide (NT-proBNP) were assessed centrally. Creatinine clearance was calculated according to the Cockcroft formula.

### Safety

Safety was assessed by spontaneously reported adverse events, physical examination, recording of vital signs at each visit, and laboratory tests (including NT-proBNP) at least at baseline and final visit. A cardiac examination with 12-lead electrocardiogram and echocardiography was performed at baseline and final visit, and in case of suspected heart failure. Hypoglycaemic events were recorded in a patient diary and were based on suggestive clinical symptoms only and graded as mild, moderate, or severe. All cardiovascular events and serious events were blindly adjudicated by a Safety Committee during meetings on a monthly basis.

### Echocardiography

All enrolled patients underwent a complete transthoracic examination performed before entry to the study and at discharge with the same equipment within each centre. Each echocardiography centre was accredited by the Core Laboratory prior to participation in the study. Special attention was paid to the morphological study of the mitral, aortic, and tricuspid valves in addition with valvular and LV function. Recordings were centralised (Biomedical Systems, Missouri, USA) and then interpreted consensually by two experts (Hôpital Louis Pradel, Lyon, France) who were blinded to the treatment group but not to echocardiography sequence. To ensure that all clinically significant and non-significant valvular abnormalities were noted, a side-by-side interpretation of pairs of echocardiographic recordings was performed, including morphological (thickness, calcification) and functional (stenosis and regurgitation) assessment. This entire reading process was carried out over 3 months at the end of the study, according to a pre-specified protocol, before the study unblinding.

Abnormal valvular thickening was considered to be present when a local or widespread thickness of more than 3 mm was observed [11].Semi quantitative assessment of valve regurgitation was made according to the recommendations of the American Society of Echocardiography [12].Mitral or tricuspid valve regurgitation was graded using the colour Doppler jet-area method. Aortic regurgitation was graded according to the ratio of the width of the colour jet to the diameter of the outflow tract. Valve regurgitation was defined and quantified as follows: absent = 0, minor = 1, mild = 2, moderate = 3, or severe = 4. If one of the two readers differed from the other by one grade, the higher rating was assigned.

### Study Oversight

The Scientific Committee designed and oversaw the conduct and analysis of the trial in collaboration with representatives of the sponsor. Data were collected, managed, and analysed by the sponsor. The statistical analysis plan was written prior to study unblinding. An Adjudication Committee reviewed all serious adverse events and all cardiac and vascular adverse events, and defined echocardiographic parameters. The manuscript was prepared by a writing group, whose members had unrestricted access to the data, and was subsequently revised by all authors. All authors decided to submit the manuscript for publication and assume responsibility for its accuracy and completeness.

### Statistical Methods

Efficacy analyses were performed in the full analysis set (FAS), which was defined as included patients who had taken at least one dose of study treatment and who have at least one baseline and one post-baseline evaluation of HbA_1c_. The safety set was defined as included patients who had taken at least one dose of study treatment and with post-baseline safety information.

The effect of treatment on HbA_1c_ change from baseline to last post-baseline value was estimated in the FAS using an analysis of covariance (general linear model) with baseline and country (fixed effects) as covariates. The non-inferiority of benfluorex versus pioglitazone was studied using a non-inferiority margin set at 0.4%. The estimate (E) of the treatment difference is provided, together with standard error (SE), two-sided 95% confidence interval (CI), and corresponding p value. The same model was used to provide estimates of the treatment effect for secondary criteria. For LDL cholesterol, the superiority of benfluorex versus pioglitazone was tested and p value provided. Post-hoc analyses were performed to provide additional information on safety and efficacy.

For NT-proBNP, the last post-baseline value under treatment was compared between groups using an analysis of covariance (general linear model) on log_10_-transformed value with log_10_-transformed baseline and country (fixed effects) as covariates.

The rates of emergent morphological and/or functional valvular abnormalities were compared between treatment groups and odds ratios (ORs) and corresponding 95% CIs and p values were provided (Wald test). The robustness of the findings was checked using a post hoc multivariable analysis with logistic regression adjusting for multiple covariates was performed on this clinical event. The final model was obtained using a backward selection method including the treatment group and the following characteristics at baseline: age, gender, BMI, smoking habits, diabetes duration, creatinine clearance, hsCRP, HbA_1c_, LDL cholesterol, high-density lipoprotein (HDL) cholesterol, medical history of hypertension, use of statins, and morphological and functional valvular abnormalities.

Sample size was estimated on the change from baseline to last value of HbA_1c_, to demonstrate the non-inferiority of benfluorex versus pioglitazone, using a one-sided Student *t* test with a 2.5% type I error. The study protocol initially planned that, for a 1.5% SD and a 0.40% for the clinical equivalence margin, it was necessary to recruit 479 subjects per group in the FAS to demonstrate non-inferiority if the true difference was equal to zero (±0.05%), with a power of at least 95%. It was therefore planned to include 1000 patients (500 in each group), with at least 50% with baseline HbA_1c_ >8%; the number was reduced to 840 included patients (800 patients in the FAS, with at least 420 included patients with HbA_1c_ >8%) since a blinded re-estimate of the variance of HbA_1c_ indicated that the SD was lower (1.1%) than expected (1.5%).

All statistical analyses were performed using SAS software (version 8.2, SAS Institute, Cary, NC).

## Results

Between March 2006 and January 2008, 847 patients were included and 846 randomly allocated to treatment (423 benfluorex, 423 pioglitazone). Follow-up ended in January 2009. The trial profile is presented in **Figure 1**. The safety set (SS) comprised 844 patients and FAS 830 patients. A total of 683 patients had at least one baseline and one post-baseline echocardiography, of which 615 (73%) were evaluable for morphology and function on at least one valve (310 benfluorex, 305 pioglitazone).

There were no relevant differences in demographic and baseline characteristics between the two groups (**Table 1**). Overall, the population was aged 59.1±10.5 years, and had type 2 diabetes for 7.1±6.0 years. Patients were mildly obese (BMI, 29.5±4.0 kg/m^2^) with moderate blood glucose disequilibrium (HbA_1c_, 8.3%±0.8%). Comorbidities such as hypertension (506 [60%]) and dyslipidaemia (357 [42%]) were frequent, though only 86 (10%) had a history of macrovascular complications and 34 (4%) microvascular complications.

Mean treatment duration was 331±97 days. Most of the patients in the FAS were receiving maximal dose after 4 months (313 patients (82%) with benfluorex 450 mg/day, 296 patients (73%) with pioglitazone 45 mg/day, p = 0.006). Mean HbA_1c_ decreased from baseline to final value in both groups (–0.54%±1.12% with benfluorex, –0.88% ±1.24% with pioglitazone). Changes over time are presented in **Figure 2** **.** The non-inferiority of benfluorex versus pioglitazone was not confirmed (between-group difference 0.33, 95% CI (0.17 to 0.49), p = 0.19). The last HbA_1c_ value was 7.8%±1.3% with benfluorex and 7.5%±1.3% with pioglitazone reaching a difference between groups of 0.33%, 95% CI (0.17 to 0.49). Mean last FPG was higher with benfluorex than with pioglitazone (8.7±2.8 versus 8.1±2.6 mmol/L, p = 0.002). Last LDL cholesterol concentration was lower with benfluorex than with pioglitazone (2.87±0.79 versus 3.03±0.86 mmol/L, p = 0.005). Last HDL cholesterol level was higher in pioglitazone than in benfluorex group (1.28±0.33 versus 1.25±0.32 mmol/L, p<0.001). At final evaluation, both mean waist circumference and bodyweight were lower in the benfluorex group than in the pioglitazone group (waist circumference: 98.6±11.4 versus 104.1±12.6 cm, p<0.001; bodyweight: 78.2±14.3 Kg versus 84.8±16.0 Kg, p<0.001).The mean weight from baseline to last value was moderately decreased in the benfluorex group (−1.6±3.5 kg, p<0.0001) while it increased in the pioglitazone group (3.3±4.2 kg, p<0.0001).

The mean treatment duration in the patients with analyzable echocardiograms was 346±79 days and was similar in the two groups. The mean time between baseline and final echocardiogram was 352±74 days. LV function (LV ejection fraction) and filling pressure parameters were normal and similar in the two groups at baseline and discharge (**Table 2**). Median NT-proBNP concentration was moderately and similarly increased from baseline to last value in both groups from 45.0 to 63.0 pg/mL with benfluorex and from 49.0 to 70.0 pg/mL with pioglitazone. Since the tricuspid regurgitation trace was often inadequate for peak velocity quantification, systolic pulmonary arterial pressure was evaluable in only 68 patients, and no clinically relevant change was detected. Systolic pulmonary arterial pressure above 40 mm Hg after baseline was detected in 4/30 patients with benfluorex and 9/38 patients with pioglitazone.

About half of the patients had at least one morphological valvular abnormality at baseline, mainly mild thickening, while 84% had at least one functional abnormality in any valve, mostly grade 1 regurgitation (**Table 3** **)**. Mild aortic valve stenosis was found in 11 (2%) patients and mitral stenosis in 3 (0.5%) patients. The prevalence was similar in the two groups.

During the study, 12 (2%) patients had emergent morphological abnormalities (8 patients [3%] with benfluorex, 4 [1%] patients with pioglitazone) (OR 1.99, 95% CI 0.59–6.69, p = 0.26). Emergent regurgitation (new or increased by at least one grade) occurred more frequently with benfluorex (82 [27%] patients) than with pioglitazone (33 [11%] patients) (OR 2.97, 95% CI 1.91–4.63, p<0.0001) (**Table 4**). Regurgitations more frequently involved the aortic valve (42 [14%] patients with benfluorex, 3 [1%] patients with pioglitazone) (OR 15.52, 95% CI 4.76–50.66, p<0.0001) (**Figure 3**) than the mitral valve (21 [7%] patients with benfluorex, 14 [5%] patients with pioglitazone) (OR 1.58, 95% CI 0.79–3.16, p = 0.19) or the tricuspid valve (33 [11%] patients with benfluorex, 17 [6%] patients with pioglitazone) (OR 2.01, 95% CI 1.10–3.70, p = 0.024). Most of these emergent regurgitations were rated as grade 1 (81 [27%] patients with benfluorex, 30 [10%] patients with pioglitazone) (OR 3.25, 95% CI 2.06–5.13, p<0.0001). Emergent regurgitation graded 2 was detected in 2 (0.7%) patients with benfluorex and 3 (1%) patients with pioglitazone (p = 0.64). No moderate or severe regurgitation and no valvular stenosis occurred during the study.

These emergent valvular abnormalities were not associated with any clinical signs. In benfluorex group, NT-proBNP median level reached 70.5 pg/mL in patients with emergent regurgitation and 73.0 pg/mL in patients without emergent regurgitation.

Multivariable analysis identified the following predictors for emergent valvular regurgitation in the overall population: systemic hypertension at baseline (OR 0.58, 95% CI 0.37–0.90), female gender (OR 1.61, 95% CI 1.04–2.48), morphological abnormalities at baseline (OR 1.78, 95% CI 1.14–2.78), and treatment with benfluorex (OR 3.20, 95% CI 2.03–5.05). When considering only the benfluorex group, similar odds ratios were found for morphological abnormalities at baseline (OR 2.23, 95% CI 1.28–3.88) and female gender (OR 1.83, 95% CI 1.06–3.16), whereas none were found significant in the pioglitazone group, likely due to the low number of emergent regurgitations.

Six patients died during the trial: 2 in benfluorex group (metastatic neoplasm, and acute renal failure subsequent to surgery for metastatic ovarian cancer) and 4 in pioglitazone group (road traffic accident, plasmacytoma, aspergillosis, and myocardial infarction). None of the deaths was considered as treatment-related according to the investigators or Adjudication Committee. Emergent adverse events leading to treatment interruption are listed in **Table 5**. The total number of patients with at least one serious adverse event was 32 in benfluorex group and 37 in pioglitazone group. Emergent suspected hypoglycaemia affected less frequently benfluorex (38 [9%]) than pioglitazone (56 [13%]) patients (p = 0.052).

## Discussion

REGULATE shows that benfluorex was less effective at reducing HbA_1c_ than pioglitazone and was responsible for an increase in valvular regurgitation with an estimated odds ratio of nearly 3. Of note**,** our study planned an echocardiographic follow**-**up and aimed to take into account all degrees of valvular regurgitation, including grade 1 cases. This is the largest prospective controlled study to assess the development of cardiac valvular abnormalities with benfluorex, and therefore supports a causal relationship. The echocardiography study was designed to evaluate changes in valvular regurgitation with time. Side-by-side assessment of echocardiograms eliminated potential bias due temporal variability or drift, as reported in serial echocardiography observations [13].In this population of diabetic patients with a high prevalence of baseline minor valvular abnormalities, we observed a significant increase in the incidence of valvular regurgitation with benfluorex (82) versus pioglitazone (33) (OR 2.97, 95% CI (1.91 to 4.63), p<0.0001). These regurgitations were mainly grade 1 and located on the aortic valve. None of them was associated with any clinical symptoms or adverse events. Most affected the aortic valve and cannot be related to volume overload. Moreover, in pioglitazone group, despite a significant weight gain partly related to hypervolemia, there was a smaller increase in mitral and aortic regurgitation, though a protection of pioglitazone against endocardial alteration seems unlikely. There was no significant change in valvular morphology between the two groups. As we identified valvular thickening at baseline in more than 50% of patients and did not quantify the increase in thickness, we failed to observe any significant morphologic changes. In addition, the assessment of valvular thickening may vary with the machine-settings however the randomized design of our trial should avoid any systematic bias. We did not observe any restrictive valvular motion as previously described by Van Camp et al in pergolide induced valvulopathy [14].

Drug-induced valvular heart disease is characterised by diffuse collections of thick white plaques composed of myofibroblasts. These plaques are deposited on the endocardial surfaces. Echocardiography usually shows thickening of both valve leaflets and the subvalvular apparatus. In the mitral valve, thickening and shortening of chordae tendinae cause tethering of the posterior leaflet responsible for mal-coaptation of leaflets and valvular regurgitation.

This detrimental effect of benfluorex occurred despite a relatively short-term exposure (about 1 year), but at higher cumulative doses (mean 151.5 g) than those recently reported to be responsible for severe regurgitation [9].Indeed, a retrospective analysis of a cohort of 1 million diabetic patients (4% treated with benfluorex) reported a dose-effect relationship, with a relative risk of hospitalisation of 2.9 (95% CI 2.2–3.7) [9]. Patients with lower cumulative doses (<40.5 g) were less likely to be hospitalised for valvular insufficiency. In our study, despite the high cumulative dose, no severe valvular regurgitation was observed over 1 year’s exposure. Our findings regarding the emergence of valvular regurgitation were consistent in term of odds ratio with the relative risk of hospitalisation for valvular disease reported in this retrospective study [9].

Long treatment duration and high doses of fenfluramine have been shown as predictors of fibrotic valvular regurgitation [15]. The prevalence of valvular abnormalities increased in patients treated with fenfluramine/phentermine versus controls, after only 6 months’ exposure and mainly resulted in mild grades of aortic regurgitation [15], [16]. Similar results were found in patients treated with dexfenfluramine [13]. From case-control reports, evidence exists for severe mitral regurgitation requiring surgery [8].

Benfluorex, a fenfluramine derivative, was prescribed in type 2 diabetes patients but was often misused as an appetite suppressant. Norfenfluramine is one of the active metabolites of benfluorex [4], and may be involved in the development of valvular heart disease in exposed patients through an agonist effect on the 5-HT_2B_ receptors. The role of 5-hydroxytryptamine (5-HT), its transporter (5-HTT), and 5-HT_2B_ receptors has been established in various pathological and experimental studies[17]–[21]. Reduced 5-HT inactivation and therefore increased bioavailability of 5-HT was the first proposed mechanism for the valvular heart disease observed in individuals treated with fenfluramine/phentermine [20]. A key role for 5-HTT expressed by cardiac and lung tissues, was proposed through a modulation of systemic availability of 5-HT [22]. Indeed, this protein is responsible for 5-HT uptake and subsequent inactivation of 5-HT passing through the lung [22]. This may explain why patients exposed to appetite suppressants develop mainly left-sided valvular abnormalities, whereas carcinoid valve alterations are located mostly on the right side.

Among the serotonergic receptor subtypes, the 5-HT_2B_ receptor was identified as the mitogenic receptor that would be activated by all the valvulopathic drugs, such as fenfluramine, methysergide, ergotamine, pergolide, and cabergoline [23]–[27]. Previous studies in mice lacking or overexpressing 5-HT_2B_ receptors also revealed important roles for this receptor in heart morphogenesis and cardiomyocyte hypertrophy [28]. Additionally, cyproheptadine, a 5-HT_2B_ receptor antagonist, was shown to prevent toxic valvulopathy in a rat model of pergolide-induced valvular heart disease [23]. However, as underlined by Rothman et al [19], if activation of 5-HT_2B_ receptors is necessary to produce valvular heart disease, other factors such as 5-HTT expression [29], hypoxia, inflammatory cytokines, and drugs, might also determine the individual susceptibility to develop the lesion, its anatomic location and its severity [17]. This might explain why a limited subset of patients may progress to severe valvular damage, sometimes in short period of times. Our study failed to identify a group at higher risk for valvular abnormalities since multivariable analysis did not detect any clinical or biological parameter indicating a higher risk profile except for previous valvular abnormalities and gender.

Benfluorex was withdrawn from the market in 2009 with the contribution of the REGULATE trial results, which provided evidence for a causal relationship between exposure to benfluorex and valvular alterations. The French Medicine drug agency (AFSSAPS) recommends echocardiography in patients who received benfluorex.

One important unresolved question is whether progression or regression or latent development of valvular regurgitation will occur after drug withdrawal. A prospective survey is ongoing to explore this issue in the French population exposed to benfluorex.

## Supporting Information

Protocol S1 **Trial protocol.**
(PDF)

Checklist S1 **CONSORT Checklist.**
(DOC)
