# Supplementary material for: Echocardiographic Evidence for Valvular Toxicity of Benfluorex: A Double-Blind Randomised Trial in Patients with Type 2 Diabetes Mellitus
Source: PLoS One. 2012 Jun 19;7(6):e38273. doi: 10.1371/journal.pone.0038273 (PMC3378581; doi:10.1371/journal.pone.0038273)
Supplement: Protocol S1 — Trial protocol. (PDF) [file pone.0038273.s001.pdf]

INSTITUT DE RECHERCHES INTERNATIONALES SERVIER

I. R. I. S.

Direction de la Recherche et du Développement

6, place des Pléiades - 92415 Courbevoie Cedex Tel.: 01 55 72 60 00 - Fax: 01 55 72 60 11 - Telex: 610 959 F

Document title	***CLINICAL STUDY PROTOCOL***
Study title	***A one-year multicentre, international, randomised, double-blind study with comparison of benfluorex (150 mg bid or 150 mg tid) versus pioglitazone (30 mg od or 45 mg od) in combination with sulfonylurea administered orally for the treatment of type 2 diabetes.***
Study drug	***S00780***
	benfluorex
	(Mediator®, Mediaxal®, Lipascor®)
Indication	***Type II Diabetes***
Development phase	***Phase III***
Protocol code	***Protocol N° CL3-00780-148***
	EudraCT number 2005-004798-60
Sponsor	***Institut de Recherches International Servier (I.R.I.S.)***
Date of the protocol	***Final version - 28 October 2005***
International Coordinator	***Prof. Philippe Moulin, MD, PhD<br>Service d'Endocrinologie - Unité 11<br>Hôpital Cardio-Vasculaire et Pneumologie Louis Pradel<br>28, avenue Doyen Lépine – BRON BP Lyon Montchat<br>69394 Lyon Cedex 3 – FRANCE***

Amendment

N°	Final version date

CONFIDENTIAL

2. STUDY SUMMARY SHEET

**Name of the sponsor:**<br>**I.R.I.S.**<br>**6, place des Pléiades**<br>**92415 Courbevoie - FRANCE**	**Individual Study Table Referring to Part of the Dossier**	*(For National Authority Use only)*
**Name of Finished Product:**	**Volume:**	
**Name of Active Ingredient:**<br>**benfluorex**<br>***(S00780)***	**Page:**	

Title of study: A one-year multicentre, international, randomised, double-blind study with comparison of benfluorex (150 mg bid or 150 mg tid) versus pioglitazone (30 mg od or 45 mg od) in combination with sulfonylurea administered orally for the treatment of type 2 diabetes.
Protocol N°: CL3-00780-148

International coordinator: Prof. Philippe Moulin, MD, PhD
Service d'Endocrinologie - Unité 11
Hôpital Cardio-Vasculaire et Pneumologie Louis Pradel
28, avenue Doyen Lépine – BRON BP Lyon Montchat
69394 Lyon Cedex 3 – FRANCE

Study centre:
Multicentre study performed in 5 countries (France, Argentina, Germany, India, South Africa). The recruitment of patients will be competitive whatever the country.

**Study period:**<br>- Study duration for the participant: 1 year + run-in (4 weeks)<br>- Study completion date: 1st February 2008	**Study development phase: III**

Objectives: The primary objective is to demonstrate the non-inferiority of the combination benfluorex plus sulfonylurea compared to the combination pioglitazone plus sulfonylurea on the evolution of HbA1c (main criterion) over one year of treatment. Both benfluorex and pioglitazone will be administered at optimal dosage for the treatment of type 2 diabetic patients insufficiently controlled on sulfonylurea monotherapy.
The secondary objective is to demonstrate the superiority of benfluorex combined with sulfonylurea compared to pioglitazone combined with sulfonylurea on Low Density Lipoprotein (LDL) cholesterol level.
The other secondary objectives are to assess the efficacy on Fasting Plasma Glucose (FPG), the safety profile, the cardiovascular risk profile and the economic balance of the two treatments.

Methodology:
- randomised (by IVRS), double-blind, double-dummy, parallel group, comparative (benfluorex versus pioglitazone) study,
- with stratification at baseline on country and HbA1c ≤ 8% or > 8%.

Number of participants:
Planned: 950 patients analysed in the Full Analysis Set (475 by group), 1000 included with at least 500 patients with baseline HbA1c > 8%.

Diagnosis and main criteria for inclusion:
Out-patients with type 2 diabetes diagnosed according to WHO criteria, male or female aged ≥ 35 years and ≤ 80 years, BMI between 25 and 40 kg/m2 (inclusive), treated in monotherapy with a sulfonylurea at stable dose for at least 3 consecutive months prior to the selection visit and at dose ≥ 50% of the maximal recommended one with a percentage of HbA1c > 7% and ≤ 10% as measured by High Performance Liquid Chromatography (HPLC) in a central laboratory.

Study drug: Benfluorex (S00780) administered orally at the daily doses of 150 mg (dose I), then 300 mg (dose A) or 450 mg (dose B).
Benfluorex will start at inclusion visit (W0) at 150 mg. The dose will be increased in all the patients from 150 mg to 300 mg two weeks after W0 visit. At W8, possibility to increase the dose to 450 mg to obtain FPG level below 7.8 mmol/L. From W16 until W52, dose level adapted to obtain an HbA1c level < 6.5% (all measurements performed by central laboratory within 7 days before the visit). Possibility to decrease the study dose at any moment in case of safety issue.
During the whole study, all patients will maintain unchanged the dose of their usual sulfonylurea treatment except in case of severe or repeated hypoglycaemia.

Name of the sponsor: ***I.R.I.S.***<br>***6, place des Pléiades***<br>***92415 Courbevoie - FRANCE***	**Individual Study Table Referring to Part of the Dossier**<br>**Volume:**<br><br>**Page:**	*(For National Authority Use only)*
**Name of Finished Product:**<br>***Trade Name (specify the country)***		
**Name of Active Ingredient:**<br>**benfluorex**<br>***(S00780)***		

Comparator: Pioglitazone administered orally at the daily doses of 30 mg (dose A) or 45 mg (dose B).
Pioglitazone will start at W0 at 30 mg, with the possibility to increase the dose to 45 mg at W8 to obtain FPG level below 7.8 mmol/L. From W16 to W52, dose level adapted to obtain an HbA1c level < 6.5% (all measurements performed by central laboratory within 7 days before the visit). Possibility to decrease the study dose at any moment in case of safety issue.
During the whole study, all patients will maintain unchanged the dose of their usual sulfonylurea treatment except in case of severe or repeated hypoglycaemia.

Duration of treatment: a 52-week double-blind comparative study preceded by a 4- week placebo run-in period.

Criteria for evaluation:
(Central laboratory for all biological parameters).
EFFICACY MEASUREMENTS:
Primary efficacy criterion: HbA1c (%) at each visit (W0, W4, W8, W16, W28, W40 and W52).
Secondary efficacy criteria:
- LDL cholesterol at each visit.
- FPG at each visit.
- Fasting plasma insulin at W0 and W52 visit (HOMA-IR).
- Other fasting serum lipids:
    - total cholesterol, High Density Lipoprotein (HDL) cholesterol and triglycerides at each visit.
    - Non-HDL cholesterol, Apo A1, Apo B at W0, W28 and W52 visit and LDL particle size (optional) at W0 and W52 visit.
- High sensitive C Reactive Protein (hsCRP) at W0, W40 and W52 visit.
- Waist circumference at W0, W28 and W52 visit.

SAFETY MEASUREMENTS:
- Recording of adverse events (including hypoglycaemia) at each visit.
- Follow-up of cardiovascular events at each visit (Adjudication Committee).
- Centralised biochemistry (including lactic acid assessment at W0 and W52) at W0, W28 and W52 visit.
- Liver parameters follow-up (focusing on ALAT) at W0, W4, W8, W16, W28, W40 and W52 visit.
- Haematology tests at each visit with haemoglobin and haematocrit follow-up.
- NT-proBNP at W0, W028 and W52.
- Physical examination (sitting blood pressure, heart rate, body weight) at each visit.
- Echocardiography (with centralized reading) and 12- lead ECG performed at W0, W52 and in case of suspicion of heart failure.

OTHER:
- Serum library taken at W0 and W52 will be kept at the central laboratory in order to perform further tests in diabetes (no genetic test), its complication markers and safety issues if necessary.
- Economic balance.

Statistical methods:
<u>Efficacy analysis</u>:
Primary efficacy criterion: HbA1c (%)
Main analytical approach: change from baseline to the last post-baseline value
The main analysis will test the non-inferiority of benfluorex to pioglitazone on the change from baseline value to last post-baseline value using a general linear model studying treatment effect with baseline and country as covariates. The non-inferiority limit will be set at 0.4%.
Other efficacy criteria will be analysed with the same model.

**Name of the sponsor:** ***I.R.I.S.***<br>***6, place des Pléiades***<br>***92415 Courbevoie - FRANCE***	**Individual Study Table Referring to Part of the Dossier**<br>**Volume:**<br><br>**Page:**	*(For National Authority Use only)*
**Name of Finished Product:**<br>***Trade Name (specify the country)***		
**Name of Active Ingredient:**<br>**benfluorex**<br>***(S00780)***		

Safety analysis:
Descriptive statistics will be provided for adverse events (including cardiovascular events and oedemas), episodes of hypoglycaemia, laboratory tests, vital signs (blood pressure, heart rate, weight), ECG and echocardiography abnormalities.
Other analysis: Economic balance.

Contractual signatories

I, the undersigned, have read the foregoing protocol and the "Participant information and consent form" document attached to the protocol and agree to conduct the study in compliance with such documents, GCP and the applicable regulatory requirements.

	NAME	DATE	SIGNATURE
**COORDINATOR / INVESTIGATOR** :	Pr [illegible]	14/3/6	[signature]
**CENTER NUMBER** 1000			
**DIRECTOR OF THERAPEUTIC RESEARCH:**	I. Guez	8/11/05	[signature]
**DIRECTOR OF THE THERAPEUTIC RESEARCH DEPARTMENT:**	FRANCILLARD	08/11/2005	[signature]

3. TABLE OF CONTENTS

1. STANDARD COVER PAGE ... 1

2. STUDY SUMMARY SHEET ... 2

3. TABLE OF CONTENTS ... 5

4. GLOSSARY AND DEFINITIONS ... 9

5. ADMINISTRATIVE STRUCTURE OF THE STUDY ... 11

5.1. Non sponsor parties ... 11
5.2. Sponsor parties ... 14
5.3. Departments/organisations responsible for local management of the study ... 14

6. BACKGROUND INFORMATION ... 15

7. STUDY OBJECTIVES AND PURPOSE ... 17

8. STUDY DESIGN ... 18

8.1. Assessment criteria ... 18
8.2. Experimental design ... 19
8.2.1. Study plan ... 19
8.2.2. Investigational schedule ... 21
8.3. Measures to minimise bias ... 22
8.4. Study products and blinding systems ... 23
8.4.1. Products administered ... 23
8.4.2. Treatment management ... 25
8.4.3. Management of blinding systems ... 27
8.5. Premature discontinuation of the study ... 27
8.6. Source data ... 27

9. SELECTION AND WITHDRAWAL OF PARTICIPANTS ... 28

9.1. Selection criteria ... 28
9.1.1. Demographic characteristics ... 28
9.1.2. Medical and therapeutic criteria ... 28
9.1.3. Informed consent ... 28
9.2. Non-selection criteria ... 28
9.2.1. General criteria ... 28

9.2.2. Medical and therapeutic criteria 28
9.3. Inclusion criteria 29
9.4. Non-inclusion criteria 29
9.5. Additional information recorded at the selection/inclusion visit 30
9.6. Participant withdrawal criteria 30
9.6.1. Withdrawal criteria 30
9.6.2. Procedure 31

10. TREATMENT OF PARTICIPANTS 33

10.1. Treatment administered 33
10.2. Treatment dispensing 35
10.3. Previous and concomitant treatments 36
10.4. Treatment compliance 37
10.5. Disease management 37
10.6. Arrangements after the discontinuation of the study treatment 37

11. ASSESSMENT OF EFFICACY 38

11.1. Efficacy measurements 38
11.2. Methods and measurement times 38

12. ASSESSMENT OF SAFETY 39

12.1. Safety measurements 39
12.2. Methods and measurement times 39
12.3. Adverse events 41
12.3.1. Responsibilities of investigator 41
12.3.1.1. Recording of adverse events in the case report form 41
12.3.1.1.1. Events to be recorded 41
12.3.1.1.2. Recording methods 43
12.3.1.2. Follow-up of adverse events 43
12.3.1.3. Procedure for an event requiring immediate notification 44
12.3.1.4. Evaluation of causality 45
12.3.2. Responsibilities of sponsor 45
12.3.3. Responsibilities of Adjudication Committee 45

13. OTHER ASSESSMENTS NOT SPECIFICALLY RELATED TO EFFICACY OR SAFETY 46

13.1. Economic balance 46
13.2. Sample library 46

14. STATISTICS 47

14.1. Statistical analysis 47
14.1.1. Evaluation criteria 47
14.1.2. Statistical elements 47
14.1.3. Analysis sets 48
14.1.4. Statistical methodology 48
14.2. Determination of sample size 50

15. DIRECT ACCESS TO SOURCE DATA / DOCUMENTS 50

16. QUALITY CONTROL AND QUALITY ASSURANCE 51

16.1. Study monitoring 51
16.1.1. Before the study 51
16.1.2. During the study 51
16.2. Computerised medical file 52
16.3. Audit – Inspection 52
16.4. Supervisory Committees 52
16.4.1. Scientific Committee 52
16.4.2. The Adjudication Committee 53

17. ETHICS 54

17.1. Ethics Committee(s) 54
17.2. Study conduct 54
17.3. Participant information and informed consent 54
17.4. Modification of the information sheet and consent form 55

18. DATA HANDLING AND RECORD KEEPING 56

18.1. Study data 56
18.2. Data management 56
18.3. Archiving 57

19. INSURANCE 57

20. OWNERSHIP OF THE RESULTS – PUBLICATION POLICY 58

21. ADMINISTRATIVE CLAUSES 59

21.1. Concerning the company I.R.I.S. and the investigator 59
21.1.1. Persons to inform 59
21.1.2. Protocol amendment 59
21.1.3. Final study report 59
21.2. Concerning I.R.I.S. 60
21.3. Concerning the investigator 60
21.3.1. Confidentiality - Use of information 60
21.3.2. Organisation of the centre 60
21.3.3. Documentation supplied to I.R.I.S. 61

22. REFERENCES 62

23. APPENDICES 64

List of tables

Table (5.1) 1 – Non sponsor parties – Global level ... 11
Table (5.1) 2 – Non sponsor parties - France ... 13
Table (5.1) 3 – Non sponsor parties – Argentina... 13
Table (5.1) 4 – Non sponsor parties – Germany... 13
Table (5.1) 5 – Non sponsor parties – India ... 13
Table (5.1) 6 – Non sponsor parties – South Africa ... 13
Table (5.2) 1 – Sponsor parties ... 14
Table (5.3) 1 – Departments/organisations responsible for local management of the study.... 14
Table (8.4.1) 1 –Description of study products... 23
Table (8.4.1) 2 – Description of packaging... 24
Table (10.1) 1 - Description of dose levels and administration schedule ... 34
Table (10.1) 2 - Criteria for adaptation of study treatments... 35

List of figures

Figure (8.2.1) 1 – Study plan... 19
Figure (8.2.2) 1 – Investigation schedule ... 21

List of appendices

Appendix 1: World Medical Association Declaration of Helsinki ... 65
Appendix 2: Statement on insurance policy... 70
Appendix 3: General Policy for handling study data ... 76
Appendix 4: WHO criteria of diabetes mellitus... 79
Appendix 5: Summary of product characteristics of Actos® (Pioglitazone)... 80
Appendix 6: Summary of product characteristics of Mediator® (Benfluorex) ... 89
Appendix 7: Concomitant medications ... 94
Appendix 8: Hypoglycaemia... 95
Appendix 9: Blood sampling in case of overdosage ... 97
Appendix 10: Procedure to be followed in case of suspicion of heart failure ... 98
Appendix 11: Procedure to be followed in case of oedema... 99
Appendix 12: Procedure to be followed in case of anaemia ... 100
Appendix 13: Adjudication Committee Procedure ... 101
Appendix 14: Chronic Heart Failure Classification (NYHA)... 103
Appendix 15: Creatinine Clearance, Cockroft and Gault Formula... 104
Appendix 16: Protocol of Echocardiography Doppler... 105

4. GLOSSARY AND DEFINITIONS

ADMA	:	Acceptability Data Management and Analysis
ALAT	:	ALanine AminoTransferase
Apo A1	:	Apolipoprotein A1
Apo B		Apolipoprotein B
ASAT	:	ASpartate AminoTransferase
ASSE	:	Assessment (for Selection Visit)
bid	:	bis in die (twice a day)
BMI	:	Body Mass Index
cm	:	centimeter
CRF	:	Case Report Form
CRO	:	Contract Research Organisation
CSCD	:	Clinical Supply Coordination Department
CV	:	Curriculum Vitae
C°	:	Degree Celsius
DPP	:	Digital Pen and Paper
DSR	:	Drug Shipment Request
ECG	:	ElectroCardioGram
e.g.	:	exempli gratia (for example)
EMEA	:	European Medicines Agency
FAS	:	Full Analysis Set
FPG	:	Fasting Plasma Glucose
g/dL	:	gram per deciLitre
g/L	:	gram per Litre
G/L	:	Giga (10^9) per Litre
GCP	:	Good Clinical Practice
GP	:	General Practitioner
γ-GT	:	Gamma-Glutamyl Transferase
HbA1c	:	Glycated Haemoglobin
HDL	:	High Density Lipoprotein
HOMA-IR	:	Homeostatic Model Assessment-Insulin Resistance
HPLC	:	High-Performance Liquid Chromatography
hsCRP	:	high sensitive C Reactive Protein
I.R.I.S.	:	Institut de Recherches Internationales Servier
ICH	:	International Conference on Harmonisation
i.e.	:	id est (that is)
IU	:	International Unit
IVRS	:	Interactive Voice Response System
LADA	:	Latent Autoimmune Diabetes in Adults
LDL	:	Low Density Lipoprotein
LVEF	:	Left Ventricular Ejection Fraction
MedDRA	:	Medical Dictionary for Regulatory Activities
mg/L	:	milligram per Litre
mmol/L	:	millimol per Litre
mL/min	:	milliLitre per minute
mmHg		millimetre of mercury
NGSP	:	National Glycohemoglobin Standardization Program
NSAID	:	Non Steroidal Anti-Inflammatory Drug
NT-proBNP	:	N-Terminal pro-Brain Natriuretic Peptide
NYHA	:	New York Heart Association
od	:	omni die (once a day)
OAD	:	Oral Anti-Diabetic Drug

pmol/L	:	picomol per Litre
po	:	per os (orally)
PPAR	:	Peroxisome Ploriferator-Activated Receptors
PPS	:	Per Protocol Set
QC	:	Quality Control
RS	:	Randomised Set
SAE	:	Serious Adverse Event
tid	:	ter in die (three times a day)
TZD	:	Thiazolidinediones
UKPDS	:	United Kingdom Prospective Diabetes Study
ULN	:	Upper Limit Normal
W	:	Week
WHO	:	World Health Organization
WHO-DRL	:	World Health Organisation, Drug Reference List

5. ADMINISTRATIVE STRUCTURE OF THE STUDY

5.1. Non sponsor parties

Table (5.1) 1 – Non sponsor parties – Global level

Role	Title. Initial Forename. Name	Work address and telephone number
International Coordinator	**Prof. Philippe MOULIN, MD, PhD**	Service d'Endocrinologie - Unité 11<br>Hôpital Cardio-Vasculaire et Pneumologie Louis Pradel<br>28, avenue Doyen Lépine<br>BRON BP Lyon Montchat<br>69394 Lyon Cedex 3 –FRANCE<br>+33 (0) 4 72 68 13 04
Scientific Committee	**Prof. Philippe MOULIN, MD, PhD**	Service d'Endocrinologie - Unité 11<br>Hôpital Cardio-Vasculaire et Pneumologie Louis Pradel<br>28, avenue Doyen Lépine<br>BRON BP Lyon Montchat<br>69394 Lyon Cedex 3 – FRANCE<br>+33 (0) 4 72 68 13 04
	**Prof. Eric BRUCKERT**	Hôpital Pitié Salpêtrière<br>Service Endocrinologie - Métabolisme<br>47-83 bld de l'Hôpital<br>75651 Paris Cedex 13 – FRANCE<br>+33 (0) 1 42 17 78 68
	**Prof. Geneviève DERUMEAUX**	Service d'Explorations Fonctionnelles - Laboratoire d'Echocardiographie<br>Groupement Hospitalier Est<br>Hôpital Louis Pradel<br>28, avenue Doyen Lépine<br>BRON BP Lyon Montchat<br>69394 Lyon Cedex 3 – FRANCE<br>+33 (0) 4 72 11 90 87
	**Prof. Stephen SENN**	University of Glasgow<br>Department of Statistics<br>15 University Gardens<br>University of Glasgow<br>GLASGOW<br>G12 8QQ UNITED KINGDOM<br>+ 44 (0) 141 330 5141
	**Prof. XXX**	to be announced

Adjudication Committee	**Prof. Geneviève DERUMEAUX**	Service d'Explorations Fonctionnelles - Laboratoire d'Echocardiographie<br>Groupement Hospitalier Est<br>Hôpital Louis Pradel<br>28, avenue Doyen Lépine<br>BRON BP Lyon Montchat<br>69394 Lyon Cedex 3 – FRANCE<br>+33 (0) 4 72 11 90 87
	**Prof. Hugues ROUSSET**	Service de Médecine Interne<br>Centre Hospitalier Lyon-Sud<br>69495 PIERRE-BENITE Cedex<br>FRANCE<br>+33 (0) 4 78 56 90 49
	**Dr André SERUSCLAT**	Département d'Imagerie Médicale et Endocrinologie<br>Groupement Hospitalier Est - Hôpital Louis Pradel<br>28, avenue Doyen Lépine<br>BRON BP Lyon Montchat<br>69394 Lyon Cedex 3 – FRANCE<br>+33 (0) 4 72 49 80 50

Name	Tasks	Address and telephone number
BARC	**Central laboratory**	3b, Industrie park Zwijnaarde<br>9052 GENT – BELGIUM<br>+ 32 9 329 23 23
S-CLINICA	**CRO in charge of IVRS**	6, Chaussée de Bondael<br>1050 BRUXELLES – BELGIUM<br>+ 32 2 645 05 50
KAYENTIS	**CRO in charge of Digital Pen and Paper CRF**	2, route de la Noue – BP 76<br>91193 GIF SUR YVETTE<br>FRANCE<br>+ 33 (0) 1 64 86 58 32
BIOTRIAL S.A.	**Data management, coding and statistic analysis**	Technopole Atalante Villejean<br>Rue Jean-Louis Bertrand<br>35000 Rennes – FRANCE<br>+ 33 (0) 2 99 59 91 91
To be announced	**CRO in charge of echocardiography central reading**	

Table (5.1) 2 – Non sponsor parties - France

Role	Title. Initial Forename. Name	Work address and telephone number
Coordinator	**Prof. Philippe MOULIN, MD, PhD**	Service d'Endocrinologie - Unité 11<br>Hôpital Cardio-Vasculaire et Pneumologie Louis Pradel<br>28, avenue Doyen Lépine<br>BRON BP Lyon Montchat<br>69394 Lyon Cedex 3 – FRANCE<br>+33 (0) 4 72 68 13 04

Table (5.1) 3 – Non sponsor parties – Argentina

Role	Title. Initial Forename. Name	Work address and telephone number
Coordinator	To be announced	

Table (5.1) 4 – Non sponsor parties – Germany

Role	Title. Initial Forename. Name	Work address and telephone number
Coordinator	To be announced	

Table (5.1) 5 – Non sponsor parties – India

Role	Title. Initial Forename. Name	Work address and telephone number
Coordinator	To be announced	

Table (5.1) 6 – Non sponsor parties – South Africa

Role	Title. Initial Forename. Name	Work address and telephone number
Coordinator	To be announced	

The list of investigators is given in a document attached to the protocol for each country.

5.2. Sponsor parties

Table (5.2) 1 – Sponsor parties

I.R.I.S., 6 Place des Pléiades - 92415 COURBEVOIE CEDEX (FRANCE)
Tel: (33).1.55.72.60.00 - Fax: (33).1.55.72.60.11

Role	Title. Initial Forename. Name	Work address and telephone number
Director of Therapeutic Research	David GUEZ, MD	+33 (0) 1 55 72 70 90
Director of Metabolism Division	Marie FRANCILLARD, MD, PhD<br>marie.francillard@fr.netgrs.com	+33 (0) 1 55 72 74 52
Project Director	Brigitte PICANDET, MD<br>brigitte.picandet@fr.netgrs.com	+33 (0) 1 55 72 34 34
Director of Biometry Direction	Pascale DELOFFRE, PharmD<br>pascal.deloffre@fr.netgrs.com	+33 (0) 1 55 72 65 57
Director of Pharmacovigilance Division	Francis WAGNIART, MD<br>francis.wagniart@fr.netgrs.com	+33 (0) 1 55 72 70 70

5.3. Departments/organisations responsible for local management of the study

Table (5.3) 1 – Departments/organisations responsible for local management of the study

Country	Name of the department/ organisation	Work address and telephone number
FRANCE	***EURAXI PHARMA (Monitoring)***	10, rue Guttenberg , B.P. 80325<br>37303 JOUE-LES-TOURS Cedex - FRANCE<br>Tél. : + 33 2 47 74 30 30
ARGENTINA	***To be announced***	
GERMANY	***To be announced***	
INDIA	***To be announced***	
SOUTH AFRICA	***To be announced***	

6. BACKGROUND INFORMATION

Type 2 diabetes mellitus is rapidly expanding worldwide. In 2025, the number of people with diabetes is expected to rise up to 300 millions and more than 90% of these patients will have type 2 diabetes (1). The prevalence of type 2 diabetes is associated with a high morbidity and mortality. This prevalence rises with age and therefore is expected to increase along with the demographic shift to an older population that is occurring in the developed world. Other factors contributing to the increasing prevalence are changes in lifestyle (diet, reduction in exercise, increased obesity) and a greater genetic susceptibility.

Type 2 diabetes mellitus is a state of hyperglycaemia characterised by insulin resistance, that is reduced insulin action at the level of the liver, adipose tissue and skeletal muscle as well as a progressive β-cell failure.

The incidence and progression of macrovascular and microvascular complications of type 2 diabetes are strongly associated with the degree of hyperglycaemia. Tight control of type 2 diabetes has been clearly shown to delay or prevent complications, a 1% reduction of HbA1c being associated with a 21% reduction of diabetes-related mortality (2); control of blood pressure and blood lipids must also be assessed and treated as aggressively as blood glucose in order to minimize complications from diabetes (multifactor intervention (3)).

From the release of UKPDS (United Kingdom Prospective Diabetes Study) results in 1998, the usual target value for HbA1c in patients with type 2 diabetes progressively moved down from 7% to 6.5% and even lower (4-6). To achieve such goals, early initiation of combined therapy is being recognized as desirable in type 2 diabetes, whatever be the first line of treatment. Based on the pathophysiology of type 2 diabetes, combination therapy with an insulinosecretagogue and an insulinosensitizer provides a rationale approach to reduce blood glucose levels in poorly controlled type 2 diabetic patients on top of diet, lifestyle counseling and exercise. In the UKPDS, addition of metformin (insulinosensitizer) to sulfonylurea (insulinosecretagogue) led to successful metabolic control.

Thiazolidinediones (TZD) represent a new class of insulino-sensitizing agents which activates nuclear receptors called peroxisome proliferators-activated receptors (PPAR)γ, which are expressed predominantly in adipose tissue and regulate the transcription of genes involved in adipocyte differentiation, and glucose and lipid metabolism. Thiazolidinediones are recommended in combination therapy when sulfonylurea in monotherapy at maximum tolerated dose has failed to obtaining blood glucose control. Thiazolidinediones expand plasma volume and may cause fluid retention with anaemia, weight gain, and peripheral oedema, leading to contraindication to patients with cardiac failure as well as mildly elevated liver enzymes.

Benfluorex is an agent that possesses both anti-hyperglycaemic and hypolipidaemic properties. Its main action in diabetic patients is to improve insulin sensitivity, as demonstrated in 3 double-blind, placebo-controlled studies using the euglycaemic (7;8) or the isoglycaemic (9) clamp technique. Its mode of action is different from that of metformin or thiazolidinediones (10;11). Benfluorex improves muscle insulin resistance with an increase in the glucose transporter GLUT-4 (12;13) and in glucose oxidation (14). It also decreases hepatic glucose output with an effect on glucose neogenesis through an inhibition of phosphoenol pyruvate carboxykinase (15;16) with a concomitant decrease in mitochondrial

ß-oxidation of fatty acids through an inhibition of carnitine palmityl transferase I (11). Benfluorex has no effect on basal or stimulated insulin secretion in diabetic rats (17;18).

Although benfluorex has been used in the treatment of type 2 diabetes for many years, its efficacy in combination with other oral antidiabetic agents has mostly been demonstrated in small-scale studies (19;20). Its efficacy in type 2 diabetes in combination with sulfonylureas versus placebo was recently confirmed by the large scale study CL3-00780-146 in which a decrease of HbA1c of –1% versus placebo was observed. Moreover, a benefit or a neutral effect was observed on cardiovascular risk factors such as weight, blood pressure, triglycerides and LDL cholesterol.

Given the complementary mode of action of benfluorex and sulfonylureas, the former acting on peripheral insulin action, and the latter on insulin secretion, i.e. the two main processes altered in type 2 diabetes, benfluorex should be of particular interest in this combination.

Furthermore, the only contraindications to treatment with benfluorex are currently: individual hypersensitivity to benfluorex chlorydrate or to one of its components, and chronic pancreatitis. In addition, treatment with benfluorex does not require any specific monitoring for safety. Its combination to sulfonylureas should therefore be a good alternative to the other options in term of addition of agents acting on insulin sensitivity.

Two TZD (rosiglitazone and pioglitazone) are currently available on the market, the most prescribed in Europe being rosiglitazone. Since pioglitazone is less deleterious than rosiglitazone in regard to LDL cholesterol and serum triglycerides levels (21), pioglitazone will be the TZD used in this study.

The objective of this study is to demonstrate that benfluorex in combination with sulfonylurea allow to obtain a comparable glycaemic control in type 2 diabetic patients over 1 year in comparison to pioglitazone both treatments given in combination with sulfonylureas and when the sulfonylurea alone at maximal tolerated dose is not sufficient to reach therapeutic targets. A secondary objective, related to efficacy, is to demonstrate the superiority of benfluorex on LDL cholesterol plasma level.

All pre-clinical and clinical data are presented in the Investigator's Brochure n° 3 (22).

Benfluorex is registered in more than 50 countries and is marketed in 28 countries under different trade names: Mediator® in France, Portugal, Morocco and Vietnam, Lipophoral® in Greece, Mediaxal® in Malaysia, Hong Kong and Singapore, Lipascor® in Argentina and Venezuela.

The study will be conducted in compliance with the protocol, Good Clinical Practice (GCP) and the applicable regulatory requirement.

7. STUDY OBJECTIVES AND PURPOSE

The purpose of this study is to compare the efficacy and the safety profile of benfluorex and pioglitazone in type 2 diabetic patients, not optimally controlled on sulfonylurea, over 1 year.

The primary objective will be to demonstrate the non-inferiority of the combination sulfonylurea plus benfluorex compared to the combination sulfonylurea plus pioglitazone on the evolution of HbA1c.

Both benfluorex and pioglitazone will be administered at optimal dosage for the treatment of type 2 diabetic patients insufficiently controlled on sulfonylurea monotherapy.

The main secondary objective is to demonstrate the superiority of benfluorex combined with sulfonylurea compared to pioglitazone combined with sulfonylurea on LDL cholesterol level.

The other secondary objectives will be to evaluate and compare over 1 year both combination therapies on the following:

- Fasting Plasma Glucose evolution.
- Insulin Resistance assessed by homeostatic model assessment (HOMA-IR).
- Cardiovascular risk profile.
- Safety and acceptability.
- Cost of the two treatments through an economic balance.

8. STUDY DESIGN

8.1. Assessment criteria

The primary efficacy criterion will be HbA1c centrally measured by HPLC.

The secondary criteria will be:
Efficacy criteria:

- Low Density Lipoprotein (LDL) cholesterol assessed by direct method.
- Fasting Plasma Glucose (FPG) levels.
- Insulin, Insulin Resistance assessed by homeostatic model assessment (HOMA-IR).
- Markers of the cardiovascular risk:
    - Other fasting lipids parameters: total cholesterol, High Density Lipoprotein (HDL) cholesterol, triglycerides, non-HDL cholesterol, Apo A1, Apo B and LDL particle size (optional),
    - Waist circumference.
- Inflammatory factor : high sensitive C Reactive Protein (hsCRP).

Safety and acceptability profile will be assessed by:

- Adverse events including oedemas and cardiovascular events (for all suspected events of cardiac origin, a cardiological examination will be performed).
- Hypoglycaemia events classified according to the EMEA guidelines in major, minor and suggestive episodes.
- Physical examination including body weight.
- Cardiological examination
- N-Terminal pro-Brain Natriuretic Peptide (NT-proBNP) (23; 24).
- Laboratory centralised examinations: standard biochemistry (including hepatic parameters focusing on ALAT and lactic acid) and haematology (including haemoglobin and haemotocrit follow-up).

Other:

- **Economic outcomes**: use of a patient diary to record information regarding any adverse event and any medical, para-medical visit or complementary examination apart from the protocol occurring during the study.

- **Serum library** to perform further tests in diabetes (no genetic test), its complication markers and safety issues in case of necessity.

8.2. Experimental design

8.2.1. Study plan

The study plan is shown in Figure (8.2.1) 1.

This is a randomised, double-blind, double-dummy, parallel group, comparative, multicentre, international phase III study with therapeutic benefit. The active comparator drug used in this study is pioglitazone.

The study will be performed in **1000 type 2 diabetic out patients** (500 patients in each group) followed up by general practitioners or by specialists. The recruitment will be competitive whatever the country.

Figure (8.2.1) 1 – Study plan

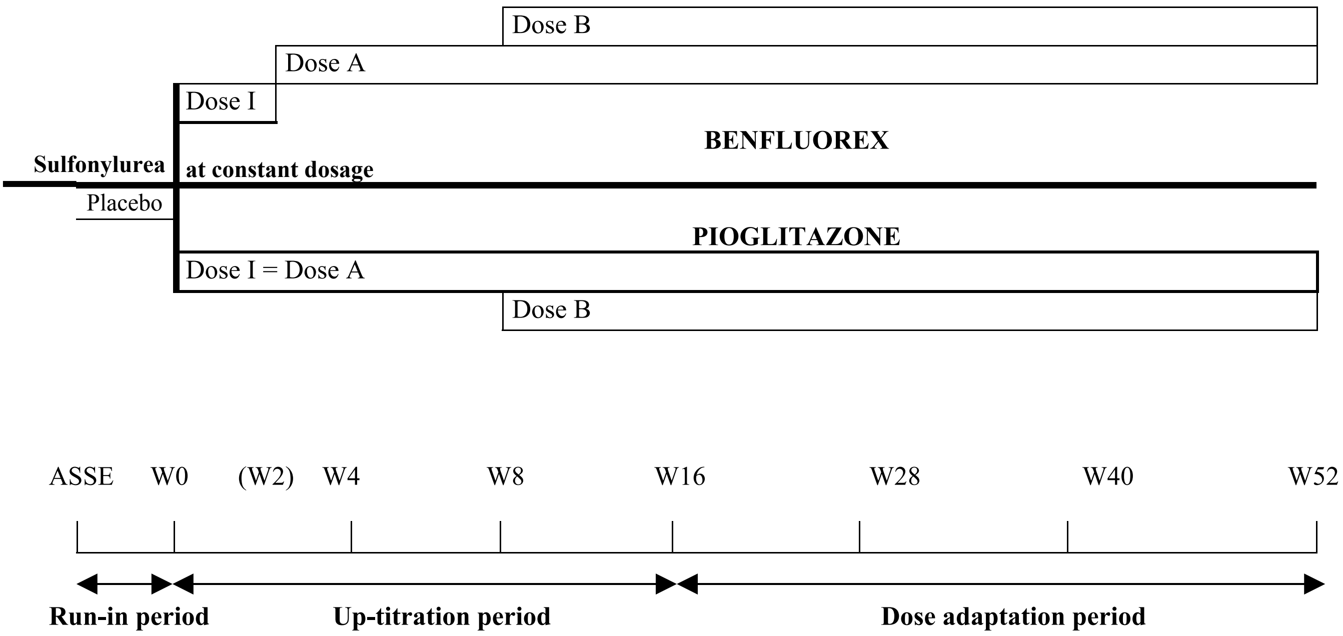

ASSE : selection visit
W0 :inclusion visit
Dose I (Initial dose) = 150 mg of benfluorex or 30 mg of pioglitazone
Dose A = 300 mg of benfluorex or 30 mg of pioglitazone
Dose B = 450 mg of benfluorex or 45 mg of pioglitazone

See § 10.1 "Treatment administered" for the description of the titration criteria.

A total of 8 clinical visits will be carried out for each patient during the study. The study will proceed as follows (see figure (8.2.2.)1 for the details of examinations):

♦ **the selection visit (ASSE)** performed 4 weeks (+/- 1 week) prior to inclusion to inform the patient about the study (written informed consent to be signed), to evaluate his/her eligibility before starting a placebo run-in period in order to evaluate the patient compliance and before performing the examinations required for the study.

After selection, and until the end of the study, all the patients will maintain unchanged their usual sulfonylurea treatment except in case of severe or repeated hypoglycemia. The patient will also receive reinforced diet and exercice advice at selection and throughout the study.

♦ **the inclusion visit (W0)** to confirm the eligibility of the patients by checking all the clinical and biological data prescribed at the selection visit as well as the use of concomitant medications and the compliance following run-in period. The eligible patient will start the randomised study for a 1-year double-blind period divided into a 16-week up-titration period and a 36-week dose adaptation period.

During the **up-titration period**, 2 visits will be performed after W0 and until W16:

♦ **visit W4** will be performed 4 weeks (± 1 week) after W0.
♦ **visit W8** will be performed 4 weeks (± 1 week) after W4.

The total duration of the titration period should not exceed 16 weeks + 2 weeks.

During the **dose adaptation period,** 4 visits will be performed from W16 until W52:

♦ **visit W16** will be performed 8 weeks (± 1 week) after W8.
♦ **visit W28** will be performed 12 weeks (± 2 weeks) after W16.
♦ **visit W40** will be performed 24 weeks (± 2 weeks) after W16.
♦ **visit W52** will be performed 36 weeks (± 2 weeks) after W16.

The total duration of the study for one patient is 52 weeks (± 2 weeks) (not taking into account the placebo run-in period between the selection visit ASSE and the inclusion visit W0).

Premature discontinuation of treatment does not necessarily mean that the patient prematurely stops the participation in the study. Information to be collected during the follow-up of these participants is given in section 9.6.2. These follow-up modalities are used to insure the efficacy and safety evaluation of all the patients who received the study drug.

8.2.2. Investigational schedule

Figure (8.2.2) 1 describes the efficacy and safety measurements assessed during the study.

Figure (8.2.2) 1 – Investigation schedule

	Selection	Inclusion	Treatment period					End of Study
			Up-titration period			Dose adaptation period		
	ASSE	W0	W4	W8	W16	W28	W40	W52
Informed consent	X							
IVRS call	X	X		X	X	X	X	
Selection / inclusion criteria	X	X						
Demography, life style, Medical history	X							
Physical examination including vital signs - weight	X	X	X	X	X	X	X	X
Cardiological examination[1]		X						X
Diet and exercise instructions	X	X	X	X	X	X	X	X
Patient diary checking		X	X	X	X	X	X	X
**Efficacy measurements**								
HbA1c, FPG		X	X	X	X	X	X	X
Fasting insulin		X						X
Fasting serum lipids[2]		X	X[3]	X[3]	X[3]	X	X[3]	X
LDL particle size (optional)		X						X
hsCRP		X					X	X
Waist circumference		X				X		X
**Safety measurements**								
Standard biology[4]		X	X[5]	X[5]	X[5]	X	X[5]	X
Lactic acid and uric acid		X						X
Creatinine clearance[6]		X						
NT-proBNP		X				X		X
Adverse events		X	X	X	X	X	X	X
Suspected hypoglycaemia		X	X	X	X	X	X	X
Concomitant treatments	X	X	X	X	X	X	X	X
Allocation of run-in placebo	X							
Allocation of study treatments		X	X	X	X	X	X	
Compliance (drug accountability)		X[7]	X	X	X	X	X	X
Economic balance	X							X
Sample library		X						X

[1] Cardiological examination performed by a cardiologist including 12-lead ECG and Doppler Echocardiography (with central reading) for W0, at W52 and in case of suspicion of heart failure.
[2] Lipids: total cholesterol, LDL cholesterol, HDL cholesterol, triglycerides, non-HDL cholesterol, Apo A1 and Apo B.
[3] Only total cholesterol, LDL cholesterol, HDL cholesterol, triglycerides.
[4] Standard biology: complete blood cells count, red blood cell indexes, differential cell count with blood smears, platelet count, total proteins, creatinine, albumin, sodium, potassium, chloride, total bilirubin, ALAT, ASAT, γ-GT, alkaline phosphatase.
[5] Haemotology and liver enzymes (ALAT, ASAT, γ-GT - according to the recommendations, if ALAT > 3 times the upper normal limit, see procedure to follow in section 12.2. *"Methods and measurements times"*.)
[6] Creatinine clearance (Cockroft and Gault formula: see appendix 15) will be calculated by the central laboratory only at W0.
[7] Compliance during the placebo run-in period.

The blood volume (included sample library) taken from the patient during the whole study will not exceed 120 mL.

8.3. Measures to minimise bias

This is a multicentre, international, randomised, double-blind, double-dummy, parallel group study.

The following methods will be used to protect the blinding vis-à-vis the participants and the investigators:

- The tablets of benfluorex and benfluorex placebo will have the same aspect (size, colour) and taste. The capsules of pioglitazone and pioglitazone placebo will also be strictly identical.

- All the packaging (blisters, boxes, labels) will be strictly identical between the two treatment groups.

- In each group, patients will receive a product and the placebo of the other drug (in the benfluorex group: 3 tablets of benfluorex and 1 capsule of pioglitazone placebo will be administered per day, and in the pioglitazone group: 3 tablets of benfluorex placebo and 1 capsule of pioglitazone will be administered per day).

- The investigator will provide the CRO in charge of IVRS with all data needed for the evaluation of the dose level increase/decrease. The CRO in charge of IVRS will be responsible for the management of dose level increase/decrease according to the treatment group following the specific procedures described below.

- Sealed code list will be available at the I.R.I.S. emergency desk. No envelop with the code will be available in centres. The Adjudication Committee is blinded. The management of the blinding system is described in the section section 8.4.3. "*Management of blinding systems*".

- Treatments will be allocated under the responsibility of the CRO responsible for the **Interactive Voice Response System (IVRS)** by non-adaptative, centralised, balanced (1:1), stratified randomisation, according to country and HbA1c (≤ 8 % or > 8 %) at W0.

The structure responsible for designing the randomisation lists will be the Biometrics Direction of I.R.I.S..

All the efficacy and safety laboratory measurements will be analysed by a **central laboratory** in blind of the treatment allocated.

Circumstances under which the code is to be broken are described in section 8.4.3. "*Management of blinding systems*".

All the echocardiographies will be assessed by a central reading structure in blind of the treatment assignment.

8.4. Study products and blinding systems

8.4.1. Products administered

The following product treatment will be given:

- Benfluorex: from 150 mg od to 150 mg tid.
- Pioglitazone: 30 mg od or 45 mg od.

A benfluorex placebo is used in the benfluorex group (dose I and dose A) and during the whole study in the pioglitazone group to maintain an administration three times daily, while a pioglitazone placebo is administered in the benfluorex group once daily (at breakfast) to protect the blinding (see table (10.1) 1 *"Description of dose levels and administration schedule"*).

Table (8.4.1) 1 provides a description of the description of the study product(s).

Table (8.4.1) 1 –Description of study products

	benfluorex	***benfluorex placebo***
Pharmaceutical form of therapeutic units	Coated tablets	Coated tablets
Unit dosage	150 mg	0 mg
Appearance, colour	White, round	White, round
Composition	benfluorex hydrochloride<br><br>Excipients: maize march, sodium hydrogen carbonate, carmellose sodium, white beeswax, titanium dioxide, ethylcellulose, glyceryl oleate, polysorbate 80, povidone, sucrose, anhydrous colloidal silica, magnesium stearate, talc	lactose monohydrate<br><br>Excipients: maize march, sodium hydrogen carbonate, carmellose sodium, white beeswax, titanium dioxide, ethylcellulose, glyceryl oleate, polysorbate 80, povidone, sucrose, anhydrous colloidal silica, magnesium stearate, talc

	pioglitazone 30 mg	***pioglitazone 45 mg***	***pioglitazone placebo***
Pharmaceutical form of therapeutic units	capsules	capsules	capsules
Unit dosage	30 mg	45 mg	0 mg
Appearance, colour	Red, opaque	Red, opaque	Red, opaque
Composition	pioglitazone hydrochloride	pioglitazone hydrochloride	lactose monohydrate
	Excipients: carmellose calcium, hydroxypropyl cellulose, lactose monohydrate, magnesium stearate		

Table (8.4.1) 2 provides a description of the packaging of the study products.

Table (8.4.1) 2 – Description of packaging

**Placebo packaging from ASSE to W0**	
Number of units of the pharmaceutical form per primary packaging	1 blister card contains 21 tablets and 7 capsules.
Number of primary packaging per secondary packaging	One smaller bag of 5 blister cards with placebo will be packed in one box (4 weeks of treatment + 1 extra-week of treatment).
Number of secondary packaging per participant and per treatment period	**<u>Visit ASSE</u>:** Box "Run-in" with placebo.<br>Each box has a detachable white label* indicating the therapeutic unit number, the batch number and the expiry date.
**Benfluorex and pioglitazone packaging from W0 to W8**	
Number of units of the pharmaceutical form per primary packaging	1 blister card contains 21 tablets and 7 capsules.
Number of primary packaging per secondary packaging	<u>Box "N°1"</u>: One smaller bag of 2 blister cards dose I will be packed in one box (2 weeks of treatment).<br><br><u>Box "N°2"</u>: One smaller bag of 3 blister cards dose A will be packed in one box (2 weeks of treatment + 1 extra week of treatment).<br><br><u>Box "W4"</u>: One smaller bag of 5 blister cards dose A will be packed in one box (4 weeks of treatment + 1 extra week of treatment).
Number of secondary packaging per participant and per treatment period	The cartridge "W0" and the box "W4" have a detachable green label* indicating the therapeutic unit number, the batch number and the expiry date.<br><br>**<u>Visit W0</u>:** Box "W0-W4" containing :<br>• **1 cartridge** "W0" containing the 2 boxes: Box "N°1" and Box "N°2"<br>• **1 Box** "W4 "

* The detachable label must be stuck on the appropriate file as indicated in the section 10.2 (Treatment dispensing)

**Benfluorex and pioglitazone packaging starting from W8**	
Number of units of the pharmaceutical form per primary packaging	1 blister card contains 21 tablets and 7 capsules
Number of primary packaging per secondary packaging	2 smaller bags of 7 blister cards will be packed in one box (treatment for 12 weeks + 2 extra-weeks).
Number of primary packaging per secondary packaging and per treatment period	Each box has a detachable blue label* indicating the therapeutic unit number, the dose, the batch number and the expiry date.<br><br>**<u>Visit W8, W16, W28, W40</u>:** Box " W8 – W40 "

*The detachable label must be stuck on the appropriate file as indicated in the section 10.2 (Treatment dispensing)

The labelling of packages complies with the local regulatory requirements, as well as the recommendations of the European Guide to Good Manufacturing Practice.

8.4.2. Treatment management

For the placebo run-in period, therapeutic unit allocation will be managed centrally by the CRO in charge of IVRS.

For the double-blind period, randomisation and therapeutic unit number allocation will be managed centrally by the CRO in charge of IVRS.

Treatments will be supplied directly from the manufacturing site to the study site or via an intermediate depot. These details as well as the method of initial and re-supply of study treatments will be described in the task specifications of the CRO responsible for the IVRS and any modifications to the procedure occurring during the study will be documented in an amendment to the initial task specifications.

The **Clinical Supply Coordination Department (CSCD)** will be responsible for:

- manufacturing of study treatments and corresponding placebo,
- packaging of treatment units,
- labelling of treatment units,
- preparation of the study drug lists and transfer them to the CRO in charge of IVRS,
- dispatching of treatment units to centres or to the intermediate depot upon request of the CRO in charge of IVRS by means of DSR (Drug Shipment Request) directly or indirectly via the therapeutic division.

Note: The CSCD may subcontract part or all of these tasks. It will have specific contracts with each subcontractor.

The **Sponsor** will be responsible for:

- opening the study centres,
- activating the study centres in the IVRS (via CRO in charge of monitoring),
- initiation of shipment of treatment units,
- follow up extra study treatment requirements,
- management of amendments to the initial task specifications of the CRO in charge of IVRS**,**
- verification on site of the study drug given to the patients (monitoring of stuck labels in the study medication dispensation page of the CRF) via CRO in charge of monitoring,
- definition of user profiles with restricted access according to their role in the study and corresponding to their qualification.

The **CRO in charge of IVRS** will be responsible for:

- central randomisation,
- management and documentation of treatment unit reception and availability in the study centres and in depots,
- informing the CSCD about the treatment unit requirements for supply or re-supply for each centre,
- management of expiry date,
- at each visit, attribution treatment unit numbers to patient according to their treatment group,
- management of dose increase and dose decrease if safety concern,
- management of code list and request for code breaking (possibility 24 hours a day and 7 days a week),
- availibility for questions concerning IVRS 24 hours a day and 7 days a week for investigators.

The **Investigator and/or the pharmacist** of the health care establishment will be responsible for:

- compliance wih the IVRS instruction manual provided prior to the study by the CRO in charge of IVRS,
- the receipt and the storage: any defect or deterioration in the study products and/or in the packaging must be notified to the monitor. This information must be entered in the IVRS at the time of acknowledgment of receipt,
- dispensing of therapeutic units (see section 10.2. *"Treatment dispensing"),*
- drug accountability of used and unused products. This accountability will be checked on a regulary basis by the study monitor,
- local destruction (by a certified structure) or return to the sponsor of treatments will be initiated by the sponsor. After destruction, a certificate of destruction with details of the treatment unit numbers destroyed will be provided to the sponsor.

The treatment should be stored in ambient, dry conditions in a secure area with restricted access.

Treatment management will be verified on a regular basis by the study monitor.

The investigator and/or the pharmacist of the healthcare establishment and/or a designated person from their study team must complete in real time all the documents provided by the sponsor concerning treatment management (acknowlegment of receipt of therapeutic unit,

therapeutic unit tracking form model “centre”, therapeutic unit tracking form model “intermediate storage” and recuperation and destruction of therapeutic unit form).

The investigator and/or the pharmacist of the healthcare establishment should only use the treatment provided for the participants involved in the study.

All defects or deterioration of treatments or their packaging are to be reported to the study monitor and/or to the IVRS. The investigator will notify the monitor of all complaints set out by a participant (change of taste, appearance…).

In the event of anticipated return of treatments to the sponsor (batch recall), the sponsor will prepare an information letter intended for the investigator and/or pharmacist of the healthcare establishment. This letter will be sent by the person locally responsible for the study to each study centre. On receipt of the letter, the investigator and/or the pharmacist will identify the participants in possession of the treatment at the time the incident becomes known, by using, among other tools, the therapeutic unit tracking form, and will contact them immediately.

8.4.3. Management of blinding systems

The blinding system is managed by the CRO in charge of IVRS.

The code for any study participant should only be broken by the investigator or authorised person if it is absolutely necessary to ascertain the type of treatment given. We remind the investigator that whatever be the antidiabetic treatment used, there is no antidote in case of overdosage. So the code have to be broken only in case of “necessity”.

If the investigator or authorized person should break the code for any participant, he/she must call the IVRS and follow instructions described in the user manual provided prior to the start of the study. The participant will discontinue the study drug.

8.5. Premature discontinuation of the study

The sponsor, after having informed the coordinators, may terminate the study before its scheduled term. Two copies of the written confirmation will be dated and signed by the investigator. The Ethics Committees and Regulatory Authorities will be informed according to local regulations.

8.6. Source data

Biological reports results with normal range values will be considered as source data.

9. SELECTION AND WITHDRAWAL OF PARTICIPANTS

9.1. Selection criteria

9.1.1. Demographic characteristics

- Male or female aged ≥ 35 years and ≤ 80 years,
- BMI between 25-40 Kg/m^2 inclusive (stable body weight),
- BMI range (25-40 Kg/m^2) could be modified in countries where definition of overweight is different. It will be specified if needed.

9.1.2. Medical and therapeutic criteria

- Type 2 diabetic outpatients diagnosed according the WHO criteria (see appendix 4).
- Currently treated in monotherapy with a sulfonylurea at stable and well-tolerated dose for at least 3 consecutive months prior to the selection visit. The sulfonylurea dose must be ≥ 50% of the maximal recommended dose according to local monography. If the sulfonylurea dose is below 50%, the investigator should give a justification.
- Not optimally controlled according to the last available HbA1c value under sulfonylurea treatment likely to fit in with inclusion criteria.
- Without severe diabetic complications which according to the investigator could interfere with the efficacy or safety evaluation of the study.
- Able to comply with the study procedures and follow the study instructions particularly to fill in the diary card.

9.1.3. Informed consent

Obtained as described in section 17.3. of the protocol.

9.2. Non-selection criteria

9.2.1. General criteria

- Unlikely to be compliant to study medication and visits.
- Pregnancy, breastfeeding or possibility of becoming pregnant during the study without effective contraception (investigator's judgement).
- Drug and/or alcohol abuse and patients at risk for acute alcoholic poisoning.
- Participation in another study at the same time or within 3 months prior to selection, or participant already enrolled in the present study.
- Major surgery planned during the study period.

9.2.2. Medical and therapeutic criteria

- Type 1 diabetes.
- Known Latent Autoimmune Diabetes in Adults (LADA).
- Type 2 diabetes treated in the previous 3 months with an oral hypoglycaemic agent (OHA) other than a sulfonylurea.
- Type 2 diabetes treated with more than 1 OHA in the previous 3 months.
- Insulin treatment in the previous year, except short term treatment for exceptional reason such as surgery.
- Any expected indication of chronic insulin treatment during the study (> 7 days).

- Decreasing body weight ≥ 5% or body weight loss of at least 4 kg in the previous 3 months.
- Dyslipidaemia treated with a lipid medication modified during the previous 3 months or uncontrolled dyslipidaemia requiring a lipid medication or an adaptation of lipid medication dosage at inclusion or in the 2 months following the inclusion.
- Contraindication to treatment by pioglitazone (see appendix 5: the summary of pioglitazone product characteristics) i.e.:
    - o Known hypersensitivity to pioglitazone or to any of the components.
    - o Heart failure or history of cardiac failure (NYHA grade I to IV; see appendix 14).
    - o Hepatic impairment or other evidence of liver disease.
    - o Combination with insulin.
- Contraindication to treatment by benfluorex (as referred in the product characteristics, see appendix 6) i.e.:
    - o Chronic pancreatitis.
    - o Known hypersentivity to benfluorex hydrochloride or to one of the components.
- Uncontrolled and clinically significant disease or known malignancy that could interfere with the conduct of the study limit life expectancy or require prohibited treatment (including unstable angina).
- Recent major cardiovascular event in the previous 6 months (myocardial infarction, stroke, acute ischemia).
- High blood pressure levels: Sitting systolic blood pressure > 180 mmHg or sitting diastolic blood pressure > 100 mmHg. This criterion must be rechecked at inclusion, if the values are still over these limits, the patient must not be included.
- Active known retinopathy with neo-vascularisation and/or sight-threatening macular oedema. The patient may be selected only if the retinopathy has been stable for at least 6 months following laser therapy.
- Acute or chronic condition apart from type 2 diabetes that would compromise end-points evaluation: e.g. all factors/diseases that interfere with the HbA1c analysis (serious anaemia, haemoglobinopathy, haemolysis, blood donation in the previous 12 weeks).
- History of acute pancreatitis.

9.3. Inclusion criteria

- Patient still fulfilling all the selection criteria.
- **HbA1c** measured between selection and inclusion (central laboratory, HPLC method) must be **> 7% and ≤ 10%**. At least 500 patients will have HbA1c in the range]8 % ; 10%].

9.4. Non-inclusion criteria

- Presence of non-selection criteria after the clinical and biological examinations performed since the visit ASSE and particularly if blood pressure is above the upper limit at both the selection and the inclusion visit, the patient must not be included (see section 9.2).

- Patient unable to comply with the study treatment particularly if compliance assessed at the W0 visit of run-in placebo capsules or run-in placebo tablets is > 120% or < 80%.

- Left Ventricular Ejection Fraction (LVEF) < 40% and any major cardiac pathology discovered at echocardiography according to local assessment (see appendix 16).
- Abnormal biological results:
    - ALAT > 2.5 times the Upper Limit Normal (ULN) value.
    - Haemoglobin level < 12 g/dL in men and < 11 g/dL in women.
    - Creatinine clearance < 30 mL/min according to the Cockroft and Gault formula (see appendix 15).
    - Triglycerides > 4 g/L (4,6 mmol/L.)
- Unauthorised concomitant treatments (see appendix 7).

9.5. Additional information recorded at the selection/inclusion visit

- Patient smoking habits.
- Patient physical examination.
- Patient medical and surgical history.
- History of type 2 Diabetes.
- History of sulfonylurea therapy.
- Waist circumference.

9.6. Participant withdrawal criteria

9.6.1. Withdrawal criteria

Premature discontinuation of treatment, does not necessarily mean that the patient prematurely stops the participation in the study. Information to be collected during the follow-up of these participants is given in section 9.6.2. These follow-up modalities are used to insure the efficacy and safety evaluation of all patients who received the study drug.

The reasons for premature discontinuation of treatment are:

- **Adverse events or any condition incompatible with continuation of either treatment** according to the judgement of the investigator or for which the continuation of the study is recommended (refer to the investigator's brochure of benfluorex and pioglitazone monography in appendices 5 and 6). These include:

    - **Hypoglycaemia incompatible with treatment continuation**, e.g. occurrence of one episode of severe hypoglycaemia or repeated non severe hypoglycaemia (> 3 per month) if the sulfonylurea dose can not be decreased anymore.

    - **ALAT increase > 3 times the ULN value**, after 2 consecutive tests performed in 7 days apart (see appendix 5).

    - **Cardiac failure** (see appendix 10).

    - **Clinically significant anaemia** defined by an haemoglobin level < 12g/dL in men and < 11g/dL in women and a decrease ≥ 1.5 g/dL in men and ≥ 1 g/dL in women (see appendix 12).

- **Major deviation to protocol incompatible with continuation of either treatment** (including **pregnancy**).

- **Treatment failure (lack of efficacy) defined as uncontrolled metabolic state with:**

  - **HbA1c > 9.5% at W16** or **HbA1c > 9% at W28 or W40** unless increase to dose level B is still possible. If external factors (poor compliance, change of diet and lifestyle, occurrence of infectious or inflammatory disease, intake of corticosteroids) are detected, the investigator will reinforce diet advice and recommendations about treatment intake and/or study protocol compliance. At the following study visit, if the HbA1c is still > 9% the study treatment will be stopped.
  - **or hyperglycaemia** unexplained by external factors and confirmed by either:
    - o clinical symptoms (thirst, polyuria,…) and FPG ≥ 13.9 mmol/L
    - o or no symptom and FPG ≥ 13.9 mmol/L at 2 consecutive measurements separated by an interval of 2 to 4 weeks.

- **Any medical event requiring administration of an unauthorised concomitant treatment** (see paragraph 10.3).

- **Non-medical reason** (to be carefully described).

- **Lost to follow-up**: when the investigator has no news of the participant, he/she must make every effort to contact him/her, to establish the reason for the discontinuation of treatment, and to suggest the participant for coming to an end-of-study visit. If all these attempts to contact the participant fail, the investigator can then declare the participant “lost to follow-up”. The investigator should document all these attempts in the corresponding medical file.

In cases corresponding to the above, the study treatment will be stopped and the investigator will be responsible to take all the appropriate therapeutic measures to control the glycaemic level.

9.6.2. Procedure

The investigator must record the reason(s) and the exact date of premature discontinuation of treatment in the case report form.

If more than one reason is given, the investigator must indicate the main reason.

The following data must be collected and recorded in the CRF during the withdrawal visit with a priority order (1 to 5):

- Main evaluation criterion: **HbA1c** sampling for the last value under treatment should be performed **before** stopping the study treatment and if not possible as soon as possible within 10 days after stopping the study treatment.

- **Fasting plasma glucose**, **serum insulin and lipids** (LDL cholesterol, HDL cholesterol, total cholesterol, triglycerides): sampling should be performed **before** stopping the study treatment to be considered as the last value under treatment and if not possible, as soon as possible after stopping the study treatment.

- **Full physical examination** (including sitting blood pressure, heart rate, body weight, waist circumference), **ECG 12-lead, echocardiography** and **full biology** at the time of premature discontinuation.

- **Adverse events** (including hypoglycaemic episodes), concomitant medications and compliance to the study treatment.

- **Economic outcomes**.

The investigator will call the IVRS to register the patient with the status "treatment discontinuation".

In the case of withdrawal from the study due to an adverse event (event requiring immediate notification or not), the investigator must make every effort to collect the information relating to the outcome of the event. If necessary, the information will be collected afterwards (see section 12.3.1.2.). This information will be recorded in that part of the case report form which concerns adverse events. If the investigator cannot collect the information from a visit, he must collect it from the doctor ensuring the follow-up of the participant.

If the study is stopped or if treatment is discontinued as a result of an event requiring immediate notification, the procedure described in section 12.3.1.3. is to be implemented.

10. TREATMENT OF PARTICIPANTS

10.1. Treatment administered

Benfluorex, pioglitazone and the placebo will be administered three times per day (from selection to the end of the study): one capsule and one tablet during breakfast, one tablet during lunch and one tablet during dinner.

The usual sulfonylurea treatment will be maintained throughout the study except in case of severe or repeated hypoglycaemia (> 3 episodes in a month). It will not be provided by the Sponsor.

♦ **Placebo run-in period (ASSE – W0):**

At selection, the patient will continue his/her usual sulfonylurea treatment and will start a placebo run-in period (4 weeks) in order to evaluate the patient compliance.

At visit ASSE, all patients will receive one box which will be allocated by the CRO in charge of the IVRS.

♦ **Up-Titration period (W0 - W16):**

At inclusion, the patient will be randomly assigned to one of the two treatment groups (benfluorex or pioglitazone in addition to his/her usual sulfonylurea) for the double blind comparative period (W0 – W52), which is divided into 2 periods: an up-titration period from W0 to W16 and a dose adaptation period from W16 to the end of the study.

The dose level will be up-titrated in order to reach the optimal dosage for both medications:

At visit W0, one box for the period W0-W8 will be allocated by the CRO in charge of IVRS. This box will contain one cartridge "W0" for the period W0-W4 and one box "W4" for the period W4-W8.

At W0, all the patients will receive one cartridge containing 2 treatment boxes:

- One box "N°1" (dose I)
- One box "N°2" (dose A)

All patients will start the study treatment with the box N° 1 (dose I). Two weeks after the inclusion visit, the patients will be asked to take the box N° 2 (dose A).

At W4, the investigator will give the box "W4" which is corresponding to dose A without calling IVRS.

At W8, all the patients will receive a new treatment box which will be allocated by the CRO in charge of the IVRS according to the following rules:
All the patients treated will be adapted according to the FPG value.
The dose will be increased to dose level B if FPG is higher than 7.8 mmol/l except in case of safety concerns.

♦ **Dose adaptation period (W16 - W52):**

At Visit W16, W28 and Visit W40: the dose will be adapted according to HbA1c and the safety to obtain the maximal dose tolerated by the patient. If HbA1c is higher than 6.5% at dose A with a good tolerance, the dose will be increased to dose B. At any time during the study and according to safety, the dose can be decreased from dose B to dose A.

The table (10.1) 1 *"Description of dose levels and administration schedule"* and (10.1) 2 *"Criteria for adaptation of study treatments"* summarizes the dose levels and administration schedule for both products (benfluorex and pioglitazone) and the criteria for the adaptation of study treatments.

Table (10.1) 1 - Description of dose levels and administration schedule

	**benfluorex group**			**pioglitazone group**		
	**Breakfast**	**Lunch**	**Dinner**	**Breakfast**	**Lunch**	**Dinner**
**Dose I**	benfluorex placebo [1] + pioglitazone placebo [2]	benfluorex placebo [1]	150 mg	benfluorex placebo [2] + 30 mg	benfluorex placebo [2]	benfluorex placebo [2]
**Dose A**	benfluorex placebo [1] + pioglitazone placebo [2]	150 mg	150 mg	benfluorex placebo [2] + 30 mg	benfluorex placebo [2]	benfluorex placebo [2]
**Dose B**	150 mg + pioglitazone placebo [2]	150 mg	150 mg	benfluorex placebo [2] + 45 mg	benfluorex placebo [2]	benfluorex placebo [2]

[1] A placebo is given temporarily (bid for the dose level I and od for the dose level A) to maintain an administration three times daily during the up-titration period.
[2] A placebo of the other drug (pioglitazone placebo in the benfluorex group; benfluorex placebo in the pioglitazone group) is given to protect the blind.

Table (10.1) 2 - Criteria for adaptation of study treatments

<table>
<tr><th>Visit</th><th>Criteria</th><th>Study treatment</th><th>Sulfonylurea</th></tr>
<tr><td colspan="3">Placebo run-in period (from ASSE to W0)</td><td rowspan="10">Pre-study dose maintained throughout the study except in case of severe or repeated hypoglcaemia (> 3 per month)</td></tr>
<tr><td>ASSE</td><td>/</td><td>All patients will start with placebo</td></tr>
<tr><td colspan="3">Up-titration period (from W0 to W16)
Adaptation of study treatment to maximal or optimal dose</td></tr>
<tr><td>W0</td><td>/</td><td>All patients will start with dose I (initial).</td></tr>
<tr><td>W2
(no visit)</td><td>/</td><td>All patients will receive dose A.</td></tr>
<tr><td>W4</td><td>/</td><td>All patients will receive dose A.</td></tr>
<tr><td>W8</td><td>FPG
Safety</td><td>If FPG > 7.8 mmol/l: the dose will be increased to dose B, except in case of safety concerns.
Otherwise: the dose is kept constant at dose A.</td></tr>
<tr><td colspan="3">Dose adaptation period (from W16 to W52)</td></tr>
<tr><td>W16, W28, W40</td><td>Dose increase:
HbA1c
Safety</td><td><u>For the patient receiving dose A</u>:
If HbA1c > 6.5% and no safety concerns: the dose will be increased to dose B.</td></tr>
<tr><td>W16, W28, W40</td><td>Dose decrease:
Safety</td><td><u>For the patient receiving dose A</u>:
In case of major safety concerns: the patient will be withdrawn.
<u>For the patient receiving dose B</u>:
In case of major safety concerns: the dose will be decreased to dose A or the patient will be withdrawn. In case of immediate withdrawal, see appendix 10</td></tr>
</table>

10.2. Treatment dispensing

The treatments will be dispensed at selection (ASSE), at inclusion (W0) and intermediate visits (W4, W8, W16, W28 and W40) by the investigator and/or the pharmacist of the health care institution, after prescription by the investigator or a co-investigator, in accordance with the study plan. Before the dispensing, the investigators must call the IVRS to know the therapeutic unit number for the study treatment except at W4 visit.

For the placebo run-in period, therapeutic unit allocation will be managed by the CRO in charge of the Interactive Voice Response System.

For the double-blind period, randomisation and therapeutic units allocation will be performed under the responsibility of the CRO in charge of IVRS. Treatments will be allocated by non-adaptative, centralised, balanced, stratified randomisation, according to country and HbA1c at W0 (HbA1c ≤ 8 % or HbA1c > 8 %).

The investigator will be compliant with the IVRS procedures which will be detailed in the user manual provided by IVRS prior to the start of the study.

The investigator should only use the study medication for the patients included in the study. The study medication can also be given by a co-investigator named in the "Organisation of the centre" document under investigator responsibility.

The study treatment will only be dispensed during the study. At the last visit of the study, the investigator will propose a treatment adapted to the nature of the clinical state of the participant.

The detachable portion of the label on the treatment box must be stuck in the case report form by the investigator or on the prescription form if the treatments are dispensed by a pharmacist.

In case of study treatment lost, the patient must immediately inform the investigator who will have to call the IVRS to obtain a new therapeutical unit number.

In case of dose decrease between two visits, the investigator must call the IVRS to obtain a new therapeutic unit number.

10.3. Previous and concomitant treatments

Detailed concomitant treatments are presented in Appendix 7.

Treatments not authorised during the study starting from the selection visit (ASSE) until the last visit (W52).

- All oral antidiabetic agents other than the usual sulfonylurea prescribed before ASSE authorised according to the selection criteria.
- Insulin : in case of insulin therapy for $\geq$ 7days, the patient will be withdrawn from the study (contraindicated with glitazones).
- Drugs contraindicated with sulfonylurea (miconazole is an absolute contraindication; please refer to the monography of the prescribed sulfonylurea).

Treatments only allowed for short term use in exceptional cases

- Oral and injected corticosteroids are not allowed in chronic use. These medications can only be accepted if needed for a short period (10 days maximum) at a distance of at least 1 month from the forthcoming evaluation sampling. If the delay from the evaluation sampling is inferior to one month, this evaluation sampling should be postponed accordingly.

Treatments authorised in chronic use and concomitant treatments

- Lipid lowering drugs are authorised under conditions (see section 9.2.2).

Special warnings and special precautions for use

- Concomitant administration of NSAIDs and pioglitazone may increase the risk of oedema and, therefore, should be considered with the greatest precautions.

Details of previous and concomitant treatments should be recorded by the investigator on the appropriate pages, entitled "Concomitant treatments" of the case report form.

10.4. Treatment compliance

The number of tablets and capsules dispensed and the number of tablets and capsules returned by the participant should be counted by the investigator or a designated person from his/her team and recorded in the case report form.

Each participant must record all new concomitant medication taken between visits with corresponding dates in a diary provided by the investigator.

Compliance will be assessed at the study visits W0, W4, W8, W16, W28, W40 and W52.

10.5. Disease management

Patient will receive counselling about diabetes, nutrition and physical activity at the selection visit. The prescribed diet should be maintained throughout the study without any modification. He/she will receive information about how to manage hypoglycaemia.

The patient will be provided with a diary card at the selection visit and will be instructed how to use it (e.g. record of any clinically relevant event). This diary is a source document. It must be identified with the name and surname of the patient and kept in the patient’s medical file. The patient must return it at each visit and it must be carefully controlled by the investigator.

10.6. Arrangements after the discontinuation of the study treatment

After the discontinuation of the study treatment, the participant will receive a treatment and/or have access to other appropriate care by his doctor.

11. ASSESSMENT OF EFFICACY

11.1. Efficacy measurements

Efficacy measurements performed for each visit are indicated in Figure (8.2.2)1 – Investigation schedule. Most of them are biological measurements. They include HbA1c, LDL cholesterol, FPG, fasting plasma insulin, fasting serum lipids (other than LDL cholesterol) and hsCRP.
The waist circumference will be measured.

11.2. Methods and measurement times

All efficacy biological measurements will be performed in a central laboratory. Collection and shipment procedures will be specified in a separated document available prior to the start of the study.

The main parameter for the efficacy assessment is the **HbA1c** measurement. It will be assessed at each visit: at W0, W4, W8, W16, W28, W40 and W52. The blood sample
(3 mL EDTA vacutainer) should be performed within 7 days before the corresponding visit and HbA1c will be analysed by standardised HPLC method (NGSP certification).

The first secondary criterion is the measurement of LDL cholesterol. It will be performed at each visit: at W0, W4, W8, W16, W28, W40 and W52. The blood sampling should be performed in the morning after at least 8 hours fasting. The dinner preceding this sampling should be light (no fat rich diet, no excess of alcohol).

Blood sampling for the other biological efficacy parameters will be performed in the same conditions as LDL cholesterol: FPG will be assessed at each visit. Fasting plasma insulin will be assessed at baseline and W52. Triglycerides, HDL cholesterol and total cholesterol will be assessed at each visit. Apo A1, Apo B, non-HDL cholesterol will be assessed at W0, W28 and W52, LDL size particle will be assessed at W0 and W52 (and according to countries) and hsCRP will be assessed at W0, W40 and W52.

Throughout the study, the central laboratory results will be transmitted as soon as available to the corresponding investigator, who will keep the original data in the patient file and a copy in the CRF, along with a clinical assessment.

The waist circumference will be measured at W0, W28 and W52 visit. Investigators will be provided a specific device in order to be able to perform this measurement with the same tool during the whole study.

12. ASSESSMENT OF SAFETY

12.1. Safety measurements

Safety measurements during the study, as indicated in Figure (8.2.2) 1– Investigation schedule, are:

- Recording of adverse events including hypoglycaemia at each visit.
- Reviewing of cardiovascular events by the Adjudication Committee.
- Assessing the aetiology of oedema at inclusion and during the study (see appendix 11).
- Centralised biochemistry focusing on ALAT, lactic acid and haematology with haemoglobin, haematocrit follow-up.
- NT-proBNP follow-up.
- Body weight, heart rate, blood pressure, ECG and echocardiography.

12.2. Methods and measurement times

- **Hypoglycaemic episodes:** each patient likely to be included will receive training on diabetes education by the investigator. He will be informed about trigger factors of hypoglycaemia and symptoms suggestive of hypoglycaemia and will be instructed about the procedure to follow in case of any symptom suggestive of hypoglycaemia: then he will be instructed to take immediately afterwards sugar, food or fluid containing sugar (as described in the patient's diary card) and then to report the episode in his diary card. The patient will return his diary at each visit. At the study visit, the investigator will check for occurrence of hypoglycaemic episodes by patient questioning and examination of patient's diary card. For management and specific reporting of episodes suggestive of hypoglycaemia, see appendix 8.

- **Oedemas:** the investigator will carefully monitor occurrence of pedal oedema at each study visit. If oedemas develop see appendix 11.

- **Heart failure:** In case of suspicion of heart failure, the investigator will refer the patient to a cardiologist (see appendix 10).

- **Centralised biochemistry and haematology assessments**
  All the laboratory parameters will be assessed by a central laboratory during the study. The sampling will be performed locally within the 7 days before the planned study visit in order to get the results available at the visit. The patients will have to be in fasting conditions and have stopped taking their OAD (study treatment and sulfonylurea) since at least 8 hours before sampling. Sampling and shipment materials will be provided by the central laboratory. The methods for sampling, handling and analysis will be fully detailed in the instruction manual provided by the central laboratory. The normal laboratory values will be also provided prior to the start of the study and updated if necessary during

the study. They will be present on every laboratory report. The investigator will be responsible for the clinical assessment of the biological results.

The following safety laboratory assessments will be performed:

- Standard haematology: red blood cell count, red blood cell indexes, white blood cell count, differential white blood cell count, blood smears, platelet count. They will be assessed at each visit and in case of premature discontinuation.
- Biochemistry
    - o Total proteins, creatinine, albumin, sodium, potassium, chlorides calcium, total bilirubin, alkaline phosphatase. They will be assessed at W0, W28 and W52 and in case of premature discontinuation.
    - o Lactic acid and uric acid will be assessed at W0, W52 and in case of premature discontinuation.
- NT-proBNP will be assessed centrally at W0, W28, W52, in case of premature discontinuation and in case of oedema (to evaluate an underlying heart failure).

Liver enzymes (ALAT, ASAT, γ-GT) will be assessed at W0, W4, W8, W16, W28, W40, W52 and in case of premature discontinuation.

- **If ALAT level is > 2.5 times the upper normal limit prior to inclusion, the patient should not be included.**
- **If ALAT level is > 3 times the upper normal limit after inclusion,** a re-test should be performed within 7 days. If the value remains 3 times the normal upper limit, the study treatment will be stopped and the patient withdrawn.
- **In case of unexplained symptoms suggestive of hepatic disease** (nausea, vomiting, abdominal pain, fatigue, loss of appetite, jaundice, dark urines), complete liver function tests should be performed. Until the results are obtained, the decision to maintain study treatment will relay on clinical judgement. In case of jaundice, the study treatment will be discontinued.

Haemoglobin and haematocrit will be assessed at every visit and in case of premature discontinuation.

- **If, at inclusion, haemoglobin level is < 12g/dL in men and < 11g/dL in women, the patient should not be included**.
- **If, after inclusion, clinically significant anaemia** is detected, see appendix 12.

The results (with the clinical assessment of the investigator) will be included in the CRF and one copy kept in the patient file. The investigator will report in the adverse event section of the CRF the abnormal biological parameters **only** if considered as clinically significant and not already reported/considered as pre-existing conditions without aggravation.

- **Body weight, heart rate, blood pressure, ECG and echocardiography:**
    - o The body weight will be measured at each study visit particularly in case of oedema and congestive heart failure, in the same conditions (same scales, patient lightly dressed, without shoes). The patient will be encouraged to control his body weight all along the study and to report any unusual weight gain, particularly if the onset is acute and the weight increases rapidly (around 2 kg within one week).

- o Heart rate and blood pressure will be measured at each visit in the same conditions: sitting position after a 10 minutes rest. For a given patient, blood pressure will be measured on the same arm during the whole study.
- o ECG 12-lead as well as echocardiography (with central reading) will be performed between ASSE and W0 (to be available at inclusion visit), at W52 and in case of suspicion of heart failure.

12.3. Adverse events

At each visit, adverse events will be recorded in the CRF (see section 12.3.1.). Assessment will be performed by questioning the patient and by physical examination including vital signs (sitting systolic and diastolic blood pressure, heart rate).

All adverse events must be followed up, fully and precisely documented to enable assessment of the safety of the study drug.

12.3.1. Responsibilities of investigator

12.3.1.1. Recording of adverse events in the case report form

12.3.1.1.1. Events to be recorded

An adverse event is defined as any untoward medical occurrence in a subject participating in a clinical study, whether or not there is a causal relationship with the study drug and/or experimental procedures, occurring or detected from the participant's signature of information and consent form, whatever the period of the study (periods without administration of the study drug are also concerned).

The investigator must therefore document as an adverse event:

- any unfavourable and unintended sign, including an abnormal finding from an additional examination (laboratory tests, X-rays, ECG, …) deemed clinically relevant by the investigator,
- any symptom (including symptoms suggestive of hypoglycaemia) or intercurrent disease,
- any worsening during the study of a symptom or a disease already present when the participant entered the study (increase in frequency and/or intensity),
  and which:
    - o is detected during a study visit or at an additional examination,
    - o occurred since the previous study visit and is notified by the participant,
    - o is documented by the participant in his/her diary card, when the investigator considers it clinically relevant.

An adverse event must be **notified immediately** when it is:

- a **serious adverse event**, i.e. an event which, whatever the dose of the study drug administered:
    - o **results in the death of the participant**,
    - o **is life-threatening**:

Note: the term “life-threatening” refers to an event in which the patient was at risk of death at the time of the event; it does not refer to an event which hypothetically might have caused death if it were more severe,

- **is medically important**:

Note: any event that may not be immediately life-threatening or result in death or hospitalisation, but may jeopardise the participant or may require intervention to prevent one of these outcomes (for example: oedema or allergic bronchospasm that required intensive treatment at home, blood dyscrasia, convulsions that do not result in hospitalisation, or development of drug dependence or drug abuse, heart failure, myocardial infarction…). The investigator should exercise his/her scientific and medical judgement to decide whether or not such an event requires expedited reporting to sponsor

- **requires inpatient hospitalisation or prolongation of existing hospitalisation**,
- **results in persistent or significant disability / incapacity**:

Note: any event that seriously disrupts the ability of the participant to lead a normal life, in other words leads to a persistent or permanent significant change, deterioration, injury or perturbation of the participant's body functions or structure, physical activity and/or quality of life,

- **an overdosage of the study drug:**

Note: any accidental or deliberate overdosage with the study drug in the participant or a person in his/her family (e.g. child) must be notified, whether or not it has medical consequences (serious or non-serious adverse event, or absence of signs and symptoms).
Sofar as possible, a blood sample should be collected for possible assay of the study drug taken. The sampling technique and the method of storage are described in appendix 9.

- **pregnancy**

Should anyone of the study participant become pregnant , the investigator must:
- stop the participant’s treatment
- report the pregnancy to one of the persons named in section 12.3.1.3
- contribute to the follow-up of this pregnancy and provide the sponsor with the relevant information.

- **severe hypoglycaemia** (see appendix 8)

12.3.1.1.2. Recording methods

Adverse events must be documented on an "Adverse event" form ("Initial information" page and if necessary "Additional information" pages) according to the general instructions for completion appended in the CRF

Some sections of the page must always be completed, whether or not the event requires immediate notification. Further sections must be completed only when the event requires immediate notification.

In the case of disease progressing by episodes (chronic disease):

- if the disease is known when the participant enters the study, only worsening (increased frequency and/or intensity of the episodes/attacks) will be documented as an adverse event,
- if the disease is detected during the study and if repeated episodes enable diagnosis of a chronic disease, the episodes will be grouped on the same page "Adverse event – Initial information" which will clearly describe the diagnosis.

For the following adverse events, additional information will be requested and specific procedure should be followed:

- **Hypoglycaemia**: the episodes of hypoglycaemia will be recorded on the « Suspected hypoglycaemia form ». See specific procedure Appendix 8.
- **Oedemas:** See specific procedure Appendix 11.
- **Heart failure:** See specific procedure Appendix 10.
- **Anaemia:** See specific procedure Appendix 12.

12.3.1.2. Follow-up of adverse events

The investigator must ensure that follow-up of the participant is appropriate to the nature of the event, and that it continues until resolution. He/She must immediately inform the sponsor of any secondary worsening.

Any change in terms of diagnosis, intensity, seriousness, measures taken, causality or outcome regarding an adverse event already reported must be written up in a new complete evaluation of the event documented on the "Adverse event - Additional information" page.

If the adverse event has not resolved at the participant's final visit in the study, the participant must be followed up suitably and any information on the outcome of the event will be noted on an "Adverse event – Additional information" page.

If the follow-up of the participant is not done by the investigator him/herself (hospitalisation, followed by a specialist or the participant's general practitioner, …), the investigator will do everything to establish/maintain contact with the person/department in charge of follow-up of

the participant, so as to have additional information and report it on an "Adverse event - Additional information" page.

12.3.1.3. Procedure for an event requiring immediate notification

In the case of an event requiring immediate notification that occurs:

- during the study, or,
- during the 30 calendar days after the participant's final study visit, regardless of the supposed role of the research (study drug or experimental procedures required by the technical protocol) or,
- beyond these 30 days, irrespective of the time of onset after the end of the study, if the event is likely to be due to the research,

the investigator must:

- **note** in the participant's medical file the date on which he/she learned of the event (at a follow-up visit or a telephone contact with the participant or a third person, …),
- **immediately inform** by telephone or fax:

COUNTRY	STRUCTURE	PERSON(S) TO BE CONTACTED	PHONE NUMBER	FAX NUMBER
FRANCE	CRO	V. ENON	+33 2 47 74 30 81	+33 2 47 74 30 45
ARGENTINA	CRO			
GERMANY	CRO			
INDIA	CRO			
SOUTH AFRICA	CRO			
ALL COUNTRIES	I.R.I.S.	B. PICANDET	+ 33 1 55 72 34 34	+ 33 1 55 72 73 38
ALL COUNTRIES	I.R.I.S.	Help Desk	+ 33 1 55 72 60 00	

- complete an "Adverse event – Initial information" page of the CRF according to the general instructions appended in the CRF, and send it by fax to the persons responsible designated above, **immediately** after of being informed of this event, without waiting for the results of the clinical outcome or of additional investigations. As soon as the "Adverse event – Initial information" form is completed, and according to DPP solution, an alert e-mail will warm monitors, medical reviewers and I.R.I.S.
- provide the persons designated above, as they become available, with anonymised copies of the documents which provide additional useful information, such as hospital admission reports, reports of further consultations, laboratory test reports, reports of other examinations leading to diagnosis (where possible, the results from pre-treatment assessments should be appended for comparison with the results obtained under treatment), or the autopsy report, if autopsy is performed,

- fulfil his/her regulatory obligations to the Regulatory Authorities and/or to the Ethics Committee, in accordance with local regulations.

If an adverse event initially non-serious worsens and becomes serious, this must be reported **immediately** to the persons designated above and documented on an "Adverse event - Additional information" page. The investigator should fax **immediately** together the "Initial information" and "Additional information" pages to these persons.

If a female participant in the study becomes pregnant, the investigator must:
- stop the participant's treatment,
- complete an "Adverse event – Initial information" page of the CRF and fax it **immediately** to the person designated above for the country,
- complete a pregnancy follow-up report form (1st page) send by I.R.I.S.,
- contribute to the follow-up of this pregnancy and provide the sponsor with information concerning this follow-up (notably using the 2nd page of the pregnancy follow-up report form).

12.3.1.4. Evaluation of causality

It is important that the investigator gives his/her opinion regarding the cause-effect relationship between an adverse event and the study drug, for the following reason:
certain adverse events that occur during clinical investigations can be sufficiently significant to lead to changes in the drug development programme (for example: change in dose, study population or in the information given to participants that may lead to the preparation of new information and consent forms). This is particularly true for events suspected to be related to the study drug (adverse drug reaction) and which, in their most severe forms, are life-threatening.

In the case of a drug interaction study, the causality will be evaluated regarding the study drug and not the associated treatment(s).

12.3.2. Responsibilities of sponsor

Independently of the regulatory obligations of the investigator, the sponsor must report the pharmacovigilance data to the appropriate Authorities and to all the investigators involved, according to the requirements stated in ICH Good Clinical Practice guidelines and local regulations.

12.3.3. Responsibilities of Adjudication Committee

Refer to section 16.4.2

13. OTHER ASSESSMENTS NOT SPECIFICALLY RELATED TO EFFICACY OR SAFETY

13.1. Economic balance

For the purpose of a further pharmacoeconomic evaluation comparing the economic outcomes of the two treatments, the investigator will report on a specific page of the CRF in case of adverse events the additional information:

- duration of the illness, GP visit, specialist visit or paramedical visit in addition to the study visits,
- in case of hospitalisation: duration of hospitalisation, type of service (emergency, intensive care unit…) and prescription at discharge,
- exam and procedures other than those required by the protocol (biological, ophthalmological, ECG…),
- transportation costs to access to health care services, if any,
- any dose modification of previous treatment,
- any new medication prescribed with start and stop dates,
- substitute equipments (crutches, plaster…) if prescribed,
- any other information such as sick leaves and home helps (nurse, home cleaner…).

13.2. Sample library

Sample library will be taken at W0 and W52. The blood volume taken will be 17 mL (8,5 mL x 2). The sampling will be performed within 7 days before the corresponding visit and sent to the centralised laboratory with other tubes.
The sample library will be kept at minus 70 C° in the centralised laboratory.
At the end of the study, the Scientific Committee will decide which tests should be performed.

14. STATISTICS

14.1. Statistical analysis

A Statistical Analysis Plan will be written after finalising the protocol and definitively completed before breaking the blind. These specifications will detail the implementation of all the planned statistical analyses in accordance with the principal features stated in the protocol.

14.1.1. Evaluation criteria

Efficacy criteria

- Main criterion :
  - ✓ HbA1c (%):
    - Main analytical approach: Change from baseline to last post-baseline value

- Secondary criteria
  - ✓ LDL cholesterol (mmol/L)
  - ✓ Fasting plasma glucose (mmol/L)
  - ✓ Fasting plasma insulin (pmol/L)
  - ✓ HOMA-IR
  - ✓ Other fasting serum lipids: total cholesterol (mmol/L), HDL cholesterol (mmol/L), triglycerides (mmol/L), non-HDL cholesterol (mmol/L), Apo A1(mmol/L), Apo B (mmol/L) and LDL particle size
  - ✓ hsCRP (mg/L)
  - ✓ Waist circumference (cm)

Safety criteria

- ✓ Adverse events (including oedemas and cardiovascular events)
- ✓ Hypoglycaemia
- ✓ Physical examination (weight, BMI, blood pressure and heart rate)
- ✓ 12-lead ECG and echocardiography
- ✓ Centralised biochemistry (including lactic acid (mmol/L)), haematology and NT-proBNP (pg/mL)

14.1.2. Statistical elements

The type one error of the statistical analyses will be set at 5% in two tailed situation and 2.5% in one tailed situation.

The following descriptive statistics will be provided depending on the nature of variables:

Quantitative variable: number of observed values, mean and standard deviation, minimum and maximum, and if necessary median, first and third quartiles.

Qualitative or ordinal variable: number and percentage by class.

14.1.3. Analysis sets

Randomised Set (RS)

This set will correspond to all randomised patients.

Safety Set (SS)

This set will correspond to patients who received at least one dose of study treatment.

Full Analysis Set (FAS)

Based on the intention-to-treat principle, this set will correspond to randomised patients who have taken at least one dose of study treatment and who have at least one baseline value and one post-baseline value of HbA1c.

Per Protocol Set (PPS)

This set will correspond to patients of the Full Analysis Set with a correct treatment compliance, with a long term measure of HbA1c and whithout relevant deviation(s) which could affect the evaluation of HbA1c (primary criterion).

Based on the actual deviations, the criteria for exclusion of patients from the different data sets will be specified and updated if necessary before breaking the blind.

Different subgroups will be defined according to age, baseline value of HbA1c according to EMEA guideline on diabetes (CPMP/EWP/1080/00, 30 May 2002 or new release version).

14.1.4. Statistical methodology

<u>Study Outcome</u>

Characteristics of patients including demography, prognostic factors, baseline values of assessment criteria will be described by treatment group and overall for the RS.

Treatment duration, duration of exposure to treatment, global treatment compliance (test drugs and sulfonylurea), status of patients and reason for withdrawal, protocol deviations, concomitant treatments will be described by treatment group and overall for the RS.

Main characteristics of patients will also be described for the SS, the FAS and the PPS.

<u>Efficacy</u>

Efficacy analyses will be carried out primarily on the FAS and secondarily on the PPS.

- Main criterion

  <u>Main analysis:</u>

  The main analysis will test the non-inferiority of benfluorex to pioglitazone on the change from baseline value to last post-baseline value using a general linear model studying treatment effect with baseline and country as covariates. The estimate of treatment difference, its standard error and its 95% confidence interval will be provided.

The non-inferiority limit will be set at 0.4%. If non-inferiority is demonstrated, the superiority of benfluorex to pioglitazone will be tested.

In case of sufficient number of centers with minimal number of patients, a mixed model with center as random factor will be used as sensitivity analysis.

<u>Secondary analyses:</u>

- Secondary criteria

The same model as for the main analysis will be performed (estimate of treatment difference, standard error and 95% confidence interval will be provided). A non-parametric approach could be used if necessary.

For LDL cholesterol, superiority of benfluorex to pioglitazone will be tested.

The responder rates on main criteria will be compared using a definition of responders to be defined in the statistical plan (current definition at time of analysis).

<u>Safety</u>

Safety analyses will be carried out on the Safety Set.

- Adverse events

Number of adverse events, number and percentage of patients reporting at least one adverse event will be described by System Organ Class and Preferred Term, by treatment group and overall. The same analysis will be performed for emergent adverse events and serious adverse events.

- Hypoglycaemia, oedema and cardiovascular events:

A survival analysis will be performed in case of sufficient number of events.

- Clinical laboratory evaluation

Laboratory parameters will be described by group for the safety set using value at the visit, last value under treatment and change from baseline to last value under treatment.
Moreover, biochemistry and haematology parameters will be classified according to the laboratory reference ranges and alert values for the potentially clinically significant abnormal values, and shift tables from baseline to first event will be presented.

- Vital signs

Vital signs will be described by group using value at the visit, last post-baseline value and change between baseline and last post-baseline value.
Moreover, blood pressure and heart rate will be classified according to the abnormal low and high values.

- ECG
ECG abnormalities will be described.

- Echocardiography

Centralised LVEF will be described by group using value at the visit, last post-baseline value and change between baseline and last post-baseline value. Moreover centralised echocardiography abnormalities will be described.

14.2. Determination of sample size

Sample size is estimated on the change from baseline to last value of HbA1c, to evidence a non-inferiority of benfluorex group as compared to pioglitazone group, using the one-sided Student t test at 2.5% type I error.

For a 1.5% standard deviation and a 0.40% clinical equivalence limit, 479 subjects per group to analyse in the Full Analysis Set are necessary to show a non-inferiority between groups if the true difference is equal to zero (± 0.05%), with at least a power of 95%.

Taking into account the Full Analysis Set definition (975 patients to be analysed), about 500 patients per group will be included.

15. DIRECT ACCESS TO SOURCE DATA / DOCUMENTS

The investigator will allow the monitors, the persons responsible for the audit, the representatives of the Ethics Committee, and of the Regulatory Authorities to have direct access to source data / documents.

16. QUALITY CONTROL AND QUALITY ASSURANCE

16.1. Study monitoring

16.1.1. Before the study

The investigator will allow the monitor to visit the site and facilities where the study will take place in order to ensure compliance with the protocol requirements.

The investigator will accept to participate a training session organised during the investigators meeting. This training may concern the materials' utilisation (waist measuring tape, IVRS system, digital pen and paper (DPP)).

16.1.2. During the study

The investigator will allow the monitor to:
- inspect the site, the facilities and the material used for the study,
- meet all members of his/her team involved in the study,
- consult all of the documents relevant to the study,
- have access to the case report form,
- check that the case report forms have been filled out correctly,
- directly access source documents for comparison of data therein with the data in the case report forms,
- verify that the study is carried out in compliance with the protocol and local regulatory requirements.

If computerised medical files are used, the investigator undertakes:
- at the start of the study, to print all the medical files of all the participants,
- during the study, to print in real time each data entry and each data change.

The investigator will personally sign and date the first page of the print-out and indicate the number of pages. At each visit by the monitor, the investigator will provide all the print-outs of the medical files of the participants. The monitor will personally date and sign all pages of each print-out, and indicate on the first page, the number of pages.

If the computer system used allows the tracking of the changes made to the medical files, the investigator will supply the monitor, at each visit, with a print-out of the medical files of the participants and the records of the changes made. Each print-out will be personally dated and signed, by the investigator on the first page and on all the pages by the monitor. The number of pages will also be indicated by the investigator and the monitor on the first page.

The study monitoring will be carried out at regular intervals, depending on the recruitment rate, and arranged between the investigator and monitor.

All information dealt with during these visits will be treated as strictly confidential.

16.2. Computerised medical file

If computerised medical files are used, and if the computer system allows, no change made in the medical files by the investigator should obscure the original information. The record must clearly indicate that a change was made and clearly provide a means to locate and read the prior information. The investigator will save data at regular intervals.

The investigator must guarantee the safety of the study data in the medical files by implementing security measures to prevent unauthorised access to the data and to the computer system.

The investigator undertakes to keep:
- in the study file, all medical file print-outs signed and dated by him/her and by the monitor,
- if the computer system used allows, the documentation of the changes made during the study to the medical files in the study file,
- all original source-documents (originals of specific examinations, informed consent forms,...).

16.3. Audit – Inspection

The investigator should be informed that an audit may be carried out during or after the end of the study.

The investigator should be informed that the Regulatory Authorities may also carry out an inspection in the facilities of the sponsor and/or the study centres. The sponsor will inform all the investigators immediately upon notification of a pending inspection. Likewise, the investigator will inform the sponsor of any pending inspection.

The investigator must allow the representatives of the Regulatory Authorities and persons responsible for the audit:
- to inspect the site, facilities and material used for the study,
- to meet all members of his/her team involved in the study,
- to have direct access to study data and source documents,
- to consult all of the documents relevant to the study.

If computerised medical files are used, the investigator undertakes to provide all the source-documents and the print-outs of the medical files of the participants and, if the computer system used allows, the record of the changes made during the study.

16.4. Supervisory Committees

16.4.1. Scientific Committee

It comprises 5 members named in section 5 of this protocol "Global level".
The committee has been named by the Therapeutic Division of I.R.I.S.:
- to guarantee the high scientific quality of the clinical protocol,
- to advise the sponsor in general and on clinical issues arising the study,
- to cooperate to the study report.

16.4.2. The Adjudication Committee

It comprises 3 members named in section 5 of this protocol "Global level".
The committee has been named by the Therapeutic Division of I.R.I.S. and will be responsible for:

- Defining echocardiographic parameters for cardiovascular monitoring of patients.
- Reviewing during the course of the study of:
    - All serious adverse events,
    - Adverse events which involve any cardiac disease, vascular disease (excluding pedal oedema with normal NT-proBNP),
    - Other cardiac events such as modification of cardiovascular concomittant treatment.
    - Any clinically significant adverse event whose diagnosis is uncertain.
- Providing procedure for analysis of clinical cases in order to rule on the diagnosis and on the aetiology of the event (see the procedure detailed in appendix 13).
- Cooperating to the study report.

17. ETHICS

17.1. Ethics Committee(s)

The study protocol, the "Participant information and consent form" document, and the list of investigators document will be submitted to an independent Ethics Committee by the coordinator or the sponsor in accordance with local regulations.

The study will not start in a centre before written approval by corresponding Ethics Committee has been obtained, the local regulatory requirements have been complied with, and the signature of the technical protocol of each contractual party involved has been obtained.

17.2. Study conduct

The study will be performed in accordance with the ethical principles stated in the Declaration of Helsinki 1964, as revised in Tokyo, 2004 (see Appendix 1)

17.3. Participant information and informed consent

The investigator or a person designated by him/her is to collect written consent from each patient before, or at the latest on, the day of the first study visit. Prior to this, the investigator or his/her delegate must inform each patient of the objectives, benefits, risks and requirements imposed by the study, as well as the nature of the study products.

The patient will be provided with an information and consent form in clear, simple language. He/she must be allowed ample time to inquire about details of the study and to decide whether or not to participate in the study.

Two original information and consent forms must be completed, dated and signed personally by the patient and by the person responsible for collecting the informed consent.

If the patient is unable to read, an impartial witness should be present during the entire informed consent discussion. The patient must give consent orally and, if capable of doing so, complete, sign and personally date the information and consent form. The witness must then complete, sign and date the form together with the person responsible for collecting the informed consent.

The patient will be given one signed original information and consent form, the second original will be kept by the investigator.

A copy of the information and consent form in the language(s) of the country is given in the “Participant information and consent form” document attached to the protocol.

17.4. Modification of the information sheet and consent form

Any change to the information and consent form constitutes an amendment and must be submitted for approval to the Ethics Committee(s), and if applicable to the Regulatory Authorities.

A copy of the new version of the information and consent form in the language(s) of the country will be given in the "Participant Information and consent form" document attached to the amendment.

Such amendments may only be implemented once written approval of the Ethics Committee has been obtained, the local regulatory requirements have been complied with, and the signature of the amendment of each contractual party involved has been obtained, with the exception of an amendment needed to eliminate immediate hazards to the participants in the study.

Each participant affected by the amendment must complete, date and sign two originals of the new version of the information and consent form together with the person who conducted the informed consent discussion. He/she will receive one signed original information and consent.

18. DATA HANDLING AND RECORD KEEPING

18.1. Study data

A case report form is designed to record all the data required by the protocol and collected by the investigator. A case report form is completed for each participant.

The case report forms will be designed by I.R.I.S. and manufactured by the DPP's CRO in compliance with I.R.I.S. standard operating procedures.

The case report form will be printed on a digital duplicated paper.

The case report forms display an identification number pre-printed in five digits.

The investigator or the designated person from his/her team is agree to complete the case report form sheets, at each participant visit, and all other documents provided by the sponsor (documents relating to the treatment management…). I.R.I.S. will provide the investigators with a digital pen to complete the CRF.

All corrections and alterations of data on the case report forms must be made by the investigator or by the designated person from his/her team according to the instructions provided.

The monitor must make certain that all data are reported on the case report forms.

At the end of each visit, the investigator or co-investigator and the monitor must sign and date the case report form in order to attest respectively:
- authenticity of the data collected in the case report form and,
- coherence between the data in the case report form and those in the source documents.

After comparing the data to the source documents, and resolution of all the problems detected, the monitor will detach the original copy to be returned to the sponsor for data management processing.

18.2. Data management

Data entry, coding and processing will be performed by a CRO, BIOTRIAL, under the responsibility of the I.R.I.S. Data Management and ADMA departments.

- Data entry: Independent, blind, double data entry with a third person resolving any discrepancy between first and second entry. CLINTRIAL (version 4.3) will be used as software and data will be stored in an ORACLE database.

- Data coding: medical history, adverse events and procedures and ECG abnormalities are coded using MedDRA. Medications are coded using WHO-DRL. The coding process is described in a specification manual.

- Data transfer: CROs (centralised laboratory, IVRS, Echocardiography) provide electronic transfer of computerised data into the CRO database. Data are transferred according to a

transfer protocol issued by the CRO data manager and validated by I.R.I.S. data manager. Data are transferred from the CRO database into the I.R.I.S. database according to a transfer protocol issued by I.R.I.S.

- Data validation: performed using SAS software and according to a specification manual describing the checks to be carried out. As a result of data validation, data may require some changes. A data clarification form is issued and sent to the investigator for confirmation or correction and signature. In some cases, as mentioned in the specification manual, changes (obvious errors) are not subjected to investigator's approval. A record of these data changes will be provided to the investigator when the study is completed.

  When data validation is completed and before breaking the blind, a blind review of the data is performed according to I.R.I.S. Standard Operating Procedure.

- The computer systems used for data processing are described in specific documents at the I.R.I.S. Biometry department.

18.3. Archiving

The investigator will keep all information relevant to the study for at least 15 years after the end of the study.

19. INSURANCE

I.R.I.S. has a civil liability insurance policy (products and investigations) which covers studies in all countries except for France and Germany in which a specific indemnity system and/or mandatory insurance is set up (see appendix 2). In the event of specific policies, a copy of the insurance policy will be given by the sponsor to the investigator before the study begins

20. OWNERSHIP OF THE RESULTS – PUBLICATION POLICY

A summary report of the study will be made available after the end of the study. The final report may be submitted to relevant regulatory authorities to support a request for product registration.

I.R.I.S. recognizes the rights of the Investigators to publish data from the study in compliance with the publication policy (see appendix 3).

I.R.I.S., acting as the study sponsor, assumes full responsibility relating to this function and retains exclusive property rights over the results of the study, which it may use as it deems fit.

No publication will be allowed without the written agreement of the Publication Committee.

21. ADMINISTRATIVE CLAUSES

21.1. Concerning the company I.R.I.S. and the investigator

21.1.1. Persons to inform

In accordance with local regulations, the investigator and/or I.R.I.S. will inform, the Director of the Healthcare Establishment, the pharmacist involved in the study and the Director of the analysis laboratory.

21.1.2. Protocol amendment

If the protocol must be altered after it has been signed, the modification or amendment must be discussed and approved by the coordinator and I.R.I.S.

The protocol amendment must be drafted in accordance with I.R.I.S. standard operating procedure and signed by both parties. It must be kept with the initial protocol. The number and date of issue of the amendment must be noted on the cover page of the protocol kept by the investigator.

All amendments must be sent by the investigator(s) or the coordinator(s) or the sponsor, in accordance with local regulations, to the Ethics Committee that examined the initial protocol. They can only be implemented after a favourable opinion of the Ethics Committee has been obtained, local regulatory requirements have been complied with, and the amendment document has been signed, with the exception of an amendment needed to eliminate immediate hazards to the participants in the study.

When the submisson is performed by the investigator or the coordinator, the latter must transmit a copy of Ethics Committee’s new written opinion to the sponsor, immediately upon receipt.

Furthermore, the amendment is to be submitted to the Regulatory Authorities in accordance with local regulations**.**

In accordance with local regulations, any document describing logistical or administrative changes to the protocol will be sent for information by the investigator(s) or the coordinator(s) or I.R.I.S. to the Ethics Committee having examined the initial protocol.

21.1.3. Final study report

The study report will be drafted by I.R.I.S. or a CRO (to be determined) in compliance with I.R.I.S. standard operating procedure.

The sponsor’s representative and the international coordinator must mutually agree on the final version. One copy of the final report, must be dated and signed by the coordinator(s) and the Director of the Therapeutic Research Department.

21.2. Concerning I.R.I.S.

The company I.R.I.S. undertakes to:

- supply the investigator with adequate and sufficient information concerning the treatment(s) administered during the study to enable him/her to carry out the study,
- obtain any authorisation to perform the study **and/or** import licence for the treatments administered that may be required by the local authorities before the beginning of the study,
- provide the coordinator or the investigators annually, or with another frequency defined by the local regulations, with a document describing study progress which is to be sent to the Ethics Committees.

21.3. Concerning the investigator

21.3.1. Confidentiality - Use of information

All documents and information given to the investigator by I.R.I.S. with respect to **benfluorex** and study **CL3-00780-148** are strictly confidential.

The investigator is informed that the follow-up of the study is computer-processed. Therefore, he/she is entitled to have access and to modify the data relating to him/her by applying to the study monitor.

The investigator agrees that he/she and the members of his/her team will use the information only in the framework of this study, for carrying out the protocol. This agreement is binding as long as the confidential information has not been disclosed to the public by I.R.I.S. The clinical study protocol given to the investigator may be used by him/her or his/her colleagues to obtain the informed consent of study participants. It must not be disclosed to other parties without the written authorisation of I.R.I.S.

A subject screening log and a full identification and enrolment list of each participant will be completed and kept by the investigator who should agree to provide access on site to the auditor and/or the Regulatory Authorities. The information will be treated in compliance with professional secrecy.

The subject screening log must be completed from the moment the investigator checks that a participant could potentially take part in the study (by assessment of participant medical history during a visit or by examination of the medical file).

21.3.2. Organisation of the centre

Every person to whom the investigator delegates a part of the follow-up of the study (co-investigator, nurse…) and any other person involved in the study for this centre (cardiologist, pharmacist, …) must figure in the "Organisation of centre" document.

This document should be filled in at the beginning of the study and updated at any change of a person involved in the study in the centre.

21.3.3. Documentation supplied to I.R.I.S.

The investigator undertakes before the study begins:

- to provide his/her dated and signed English Curriculum Vitae (CV) (maximum 2 pages) or to complete in English the CV form provided by the sponsor and to send it to I.R.I.S., together with that of his/her co-investigator(s),
- to provide a detailed description of the methods, techniques, and investigational equipment, and the reference values for the parameters measured,
- to send, a copy of the Ethics Committee's opinion with details of its composition and the qualifications of its constituent members.

The CV of other members of the team involved in the study (if possible in English) will be collected during the course of the study.

22. REFERENCES

1. King H, Aubert RE, Hermann WH. Global burden of diabetes 1995-2025. Prevalence, numerical estimates, and projections. *Diabetes Care* 21:1414-1431, 1998

2. UK Prospective Diabetes Study group: Association of glycaemia with macrovascular and microvascular complications of Type II diabetes: prospective observational study (UKPDS 35). *BMJ* 321:405-412, 2000

3. Gaede P, Vedel P, Parving HH, Pedersen O. Intensified multifactorial intervention with type 2 diabetes mellitus and microalbuminuria: the Steno type 2 randomised study. *Lancet* 353:617-622, 1999

4. European Diabetes Policy Group 1999: A desktop guide to type 2 diabetes mellitus. *Diabet Med* 16:716-730, 1999

5. American Diabetes Association: Standards of Medical Care in Diabetes. *Diabetes Care* 28:S4-S36, 2005

6. International Diabetes Federation 2005. Clinical Guidelines Task force. Global Guidelines for type 2 Diabetes.

7. Bianchi R, Bongers V, Bravenboer B, Erkelens DW. Benfluorex decreases insulin resistance and improves lipid profile in obese type II diabetic patients. *Diabetes Care* 16:557-559, 1993

8. Riccio A, Vigili de Kreutzenberg S, Dorella M et al. Mechanism(s) of the blood glucose lowering action of benfluorex. *Diab Metab Rev*. 9(suppl. 1):19S-27S, 1993

9. De Feo P, Lavielle R, De Gregoris P, Bolli GB. Anti-hyperglycemic mechanisms of benfluorex in type 2 diabetes mellitus. *Diab Metab Rev*. 9(suppl. 1):35S-41S, 1993

10. Geelen MJH, Vaartjes WJ. Recherche du mécanisme d'action d'une nouvelle substance, le benfluorex, sur le métabolisme intermédiaire: étude expérimentale sur hépatocytes isolés de rat. *Pharmatherapeutica* 1:466-476, 1977

11. Kohl C, Ravel D, Girard J, Pégorier JP. Effects of benfluorex on fatty acid and glucose metabolism in isolated rat hepatocytes: from metabolic fluxes to gene expression. *Diabetes* 51:2363-2368, 2002

12. Sevilla L, Guma A, Munoz P, Testar X, Palacin M, Zorzano A. Benfluorex improves muscle insulin responsiveness in middle-aged rats previously subjected to long-term high-fat feeding. *Life Sciences* 64:25-36, 1999

13. Storlien LH, Oakes ND, Pan DA, Kusunoki M, Jenkins A. Syndromes of insulin resistance in the rat: inducement by diet and amelioration with benfluorex. *Diabetes* 42:457-462, 1993

14. Bailey CJ, Page T, Day C, Thornburn CC. Acute effect of benfluorex on glucose metabolism. *Biochem. Pharmacol*. 44:379-82, 1992

15. Zorzano A. Effects of benfluorex treatment on insulin resistance in aged rats subjected to high-fat feeding. PHA-780-013.ESP.NP 1996

16. Tielens AGM, Van der Heuvel JM, Schmitz MGJ, Geelen MJH. Effect of chronic benfluorex treatment on the activities of key enzymes of hepatic carbohydrate metabolism in old Sprague-Dawley rats. *Biochem Pharmacol* 46:1539-1544, 1993

17. Serradas P, Blondel O, Bailbe D, Portha B. Benfluorex normalizes hyperglycemia and reverses hepatic insulin resistance in STZ-induced diabetic rats. *Diabetes* 42:564-570, 1993

18. Portha B, Serradas P, Bailbe D, Blondel O, Picarel F. Efect of benfluorex on insulin secretion and insulin action in streptozotocin diabetic rats. *Diabetes Metab. Rev.* 9 suppl. 1: 575-635, 1993

19. Roger P, Auclair J, Drain P. Addition of benfluorex to biguanide improves glycemic control in obese non-insulin-dependent diabetes: a double-blind study versus placebo. *J Diabet Complic* 13:68-73, 1999

20. Stucci N, de Gregoris P, Lavielle R, Tomasi F. Therapeutic benefit of benfluorex in type II diabetic patients treated with sulfonylureas. *J Diabet Complic* 10:267-273, 1996

21. Yki-Järvinen H. Thiazolidinediones. *N Engl J Med* 351:1106-1118, 2004

22. Investigator's Brochure n° 3 (2005)

23. Apple FS, Panteghini M, Ravkilde J, Mair J, Wu A H.B., Tate J, Pagani F, Christenson RH, Jaffe AS, on behalf of the Committee on Standardization of Markers of Cardiac Damage of the IFCC. Quality Specifications for B-Type Natriuretic Peptide Assays. *Clinical Chemistry* 51:3:486-493, 2005

24. Galasko G I.W., Lahiri A, Barnes SC, Collinson P, Senior R. What is the normal range for N-terminal pro-brain natriuretic peptid? How well does this normal range screen for cardiovascular disease? *European Heart Journal* 26:2269-2276, 2005

Statistical references

Guideline EMEA "Note for guidance on clinical investigation of medical products in the treatment of diabetes mellitus" - CPMP/EWP/1080/00 30 May 2002

Brown H., Prescott R. Applied Mixed Models in Medicine. *John Wiley & Sons Ltd*, 1999

23. APPENDICES

- APPENDIX 1: World Medical Association Declaration of Helsinki
- APPENDIX 2: Statement on insurance policy
- APPENDIX 3: General Policy for handling study data
- APPENDIX 4: WHO criteria of diabetes mellitus
- APPENDIX 5: Summary of product characteristics of Actos® (Pioglitazone)
- APPENDIX 6: Summary of product characteristics of Mediator® (Benfluorex)
- APPENDIX 7: Concomitant medications
- APPENDIX 8: Hypoglycaemia
- APPENDIX 9: Blood sampling in case of overdosage
- APPENDIX 10: Procedure to be followed in case of suspicion of heart failure
- APPENDIX 11: Procedure to be followed in case of oedema
- APPENDIX 12: Procedure to be followed in case of anaemia
- APPENDIX 13: Adjudication Committee Procedure
- APPENDIX 14: Chronic Heart Failure Classification NYHA
- APPENDIX 15: Creatinine Clearance, Cockroft and Gault Formula
- APPENDIX 16: Protocol of Echocardiography Doppler

Appendix 1: World Medical Association Declaration of Helsinki

Initiated: 1964 Original: English

WORLD MEDICAL ASSOCIATION DECLARATION OF HELSINKI
Ethical Principles
for
Medical Research Involving Human Subjects

Adopted by the 18th WMA General Assembly, Helsinki, Finland, June 1964
and amended by the
29th WMA General Assembly, Tokyo, Japan, October 1975
35th WMA General Assembly, Venice, Italy, October 1983
41st WMA General Assembly, Hong Kong, September 1989
48th WMA General Assembly, Somerset West, Republic of South Africa, October 1996
and the 52nd WMA General Assembly, Edinburgh, Scotland, October 2000
Note of Clarification on Paragraph 29 added by the WMA General Assembly, Washington 2002
Note of Clarification on Paragraph 30 added by the WMA General Assembly, Tokyo 2004

A. INTRODUCTION

1. The World Medical Association has developed the Declaration of Helsinki as a statement of ethical principles to provide guidance to physicians and other participants in medical research involving human subjects. Medical research involving human subjects includes research on identifiable human material or identifiable data.

2. It is the duty of the physician to promote and safeguard the health of the people. The physician's knowledge and conscience are dedicated to the fulfillment of this duty.

3. The Declaration of Geneva of the World Medical Association binds the physician with the words, "The health of my patient will be my first consideration," and the International Code of Medical Ethics declares that, "A physician shall act only in the patient's interest when providing medical care which might have the effect of weakening the physical and mental condition of the patient."

4. Medical progress is based on research which ultimately must rest in part on experimentation involving human subjects.

5. In medical research on human subjects, considerations related to the well-being of the human subject should take precedence over the interests of science and society.

6. The primary purpose of medical research involving human subjects is to improve prophylactic, diagnostic and therapeutic procedures and the understanding of the aetiology and pathogenesis of disease. Even the best proven prophylactic, diagnostic, and therapeutic methods must continuously be challenged through research for their effectiveness, efficiency, accessibility and quality.

7. In current medical practice and in medical research, most prophylactic, diagnostic and therapeutic procedures involve risks and burdens.

8. Medical research is subject to ethical standards that promote respect for all human beings and protect their health and rights. Some research populations are vulnerable and need special protection. The particular needs of the economically and medically disadvantaged must be recognized. Special attention is also required for those who cannot give or refuse consent for themselves, for those who may be subject to giving consent under duress, for those who will not benefit personally from the research and for those for whom the research is combined with care.

9. Research Investigators should be aware of the ethical, legal and regulatory requirements for research on human subjects in their own countries as well as applicable international requirements. No national ethical, legal or regulatory requirement should be allowed to reduce or eliminate any of the protections for human subjects set forth in this Declaration.

B. BASIC PRINCIPLES FOR ALL MEDICAL RESEARCH

10. It is the duty of the physician in medical research to protect the life, health, privacy, and dignity of the human subject.

11. Medical research involving human subjects must conform to generally accepted scientific principles, be based on a thorough knowledge of the scientific literature, other relevant sources of information, and on adequate laboratory and, where appropriate, animal experimentation.

12. Appropriate caution must be exercised in the conduct of research which may affect the environment, and the welfare of animals used for research must be respected.

13. The design and performance of each experimental procedure involving human subjects should be clearly formulated in an experimental protocol. This protocol should be submitted for consideration, comment, guidance, and where appropriate, approval to a specially appointed ethical review committee, which must be independent of the investigator, the sponsor or any other kind of undue influence. This independent committee should be in conformity with the laws and regulations of the country in which the research experiment is performed. The committee has the right to monitor ongoing trials. The researcher has the obligation to provide monitoring information to the committee, especially any serious adverse events. The researcher should also submit to the committee, for review, information regarding funding, sponsors, institutional affiliations, other potential conflicts of interest and incentives for subjects.

The Declaration of Helsinki (Document 17.C) is an official policy document of the World Medical Association, the global representative body for physicians. It was first adopted in 1964 (Helsinki, Finland) and revised in 1975 (Tokyo, Japan), 1983 (Venice, Italy), 1989 (Hong Kong), 1996 (Somerset-West, South Africa) and 2000 (Edinburgh, Scotland). Note of clarification on Paragraph 29 added by the WMA General Assembly, Washington 2002. Note of Clarification on Paragraph 30 added by the WMA General Assembly, Tokyo 2004.

14. The research protocol should always contain a statement of the ethical considerations involved and should indicate that there is compliance with the principles enunciated in this Declaration.

15. Medical research involving human subjects should be conducted only by scientifically qualified persons and under the supervision of a clinically competent medical person. The responsibility for the human subject must always rest with a medically qualified person and never rest on the subject of the research, even though the subject has given consent.

16. Every medical research project involving human subjects should be preceded by careful assessment of predictable risks and burdens in comparison with foreseeable benefits to the subject or to others. This does not preclude the participation of healthy volunteers in medical research. The design of all studies should be publicly available.

17. Physicians should abstain from engaging in research projects involving human subjects unless they are confident that the risks involved have been adequately assessed and can be satisfactorily managed. Physicians should cease any investigation if the risks are found to outweigh the potential benefits or if there is conclusive proof of positive and beneficial results.

18. Medical research involving human subjects should only be conducted if the importance of the objective outweighs the inherent risks and burdens to the subject. This is especially important when the human subjects are healthy volunteers.

19. Medical research is only justified if there is a reasonable likelihood that the populations in which the research is carried out stand to benefit from the results of the research.

20. The subjects must be volunteers and informed participants in the research project.

21. The right of research subjects to safeguard their integrity must always be respected. Every precaution should be taken to respect the privacy of the subject, the confidentiality of the patient's information and to minimize the impact of the study on the subject's physical and mental integrity and on the personality of the subject.

22. In any research on human beings, each potential subject must be adequately informed of the aims, methods, sources of funding, any possible conflicts of interest, institutional affiliations of the researcher, the anticipated benefits and potential risks of the study and the discomfort it may entail. The subject should be informed of the right to abstain from participation in the study or to withdraw consent to participate at any time without reprisal. After ensuring that the subject has understood the information, the physician should then obtain the subject's freely-given informed consent, preferably in writing. If the consent cannot be obtained in writing, the non-written consent must be formally documented and witnessed.

The Declaration of Helsinki (Document 17.C) is an official policy document of the World Medical Association, the global representative body for physicians. It was first adopted in 1964 (Helsinki, Finland) and revised in 1975 (Tokyo, Japan), 1983 (Venice, Italy), 1989 (Hong Kong), 1996 (Somerset-West, South Africa) and 2000 (Edinburgh, Scotland). Note of clarification on Paragraph 29 added by the WMA General Assembly, Washington 2002. Note of Clarification on Paragraph 30 added by the WMA General Assembly, Tokyo 2004.

23. When obtaining informed consent for the research project the physician should be particularly cautious if the subject is in a dependent relationship with the physician or may consent under duress. In that case the informed consent should be obtained by a well-informed physician who is not engaged in the investigation and who is completely independent of this relationship.

24. For a research subject who is legally incompetent, physically or mentally incapable of giving consent or is a legally incompetent minor, the investigator must obtain informed consent from the legally authorized representative in accordance with applicable law. These groups should not be included in research unless the research is necessary to promote the health of the population represented and this research cannot instead be performed on legally competent persons.

25. When a subject deemed legally incompetent, such as a minor child, is able to give assent to decisions about participation in research, the investigator must obtain that assent in addition to the consent of the legally authorized representative.

26. Research on individuals from whom it is not possible to obtain consent, including proxy or advance consent, should be done only if the physical/mental condition that prevents obtaining informed consent is a necessary characteristic of the research population. The specific reasons for involving research subjects with a condition that renders them unable to give informed consent should be stated in the experimental protocol for consideration and approval of the review committee. The protocol should state that consent to remain in the research should be obtained as soon as possible from the individual or a legally authorized surrogate.

27. Both authors and publishers have ethical obligations. In publication of the results of research, the investigators are obliged to preserve the accuracy of the results. Negative as well as positive results should be published or otherwise publicly available. Sources of funding, institutional affiliations and any possible conflicts of interest should be declared in the publication. Reports of experimentation not in accordance with the principles laid down in this Declaration should not be accepted for publication.

C. ADDITIONAL PRINCIPLES FOR MEDICAL RESEARCH COMBINED WITH MEDICAL CARE

28. The physician may combine medical research with medical care, only to the extent that the research is justified by its potential prophylactic, diagnostic or therapeutic value. When medical research is combined with medical care, additional standards apply to protect the patients who are research subjects.

The Declaration of Helsinki (Document 17.C) is an official policy document of the World Medical Association, the global representative body for physicians. It was first adopted in 1964 (Helsinki, Finland) and revised in 1975 (Tokyo, Japan), 1983 (Venice, Italy), 1989 (Hong Kong), 1996 (Somerset-West, South Africa) and 2000 (Edinburgh, Scotland). Note of clarification on Paragraph 29 added by the WMA General Assembly, Washington 2002. Note of Clarification on Paragraph 30 added by the WMA General Assembly, Tokyo 2004.

29. The benefits, risks, burdens and effectiveness of a new method should be tested against those of the best current prophylactic, diagnostic, and therapeutic methods. This does not exclude the use of placebo, or no treatment, in studies where no proven prophylactic, diagnostic or therapeutic method exists. *(See footnote*)*

30. At the conclusion of the study, every patient entered into the study should be assured of access to the best proven prophylactic, diagnostic and therapeutic methods identified by the study. *(See footnote*)*

31. The physician should fully inform the patient which aspects of the care are related to the research. The refusal of a patient to participate in a study must never interfere with the patient-physician relationship.

32. In the treatment of a patient, where proven prophylactic, diagnostic and therapeutic methods do not exist or have been ineffective, the physician, with informed consent from the patient, must be free to use unproven or new prophylactic, diagnostic and therapeutic measures, if in the physician's judgement it offers hope of saving life, re-establishing health or alleviating suffering. Where possible, these measures should be made the object of research, designed to evaluate their safety and efficacy. In all cases, new information should be recorded and, where appropriate, published. The other relevant guidelines of this Declaration should be followed.

*** FOOTNOTES:**

Note of Clarification on Paragraph 29 of the WMA Declaration of Helsinki

The WMA hereby reaffirms its position that extreme care must be taken in making use of a placebo-controlled trial and that in general this methodology should only be used in the absence of existing proven therapy. However, a placebo-controlled trial may be ethically acceptable, even if proven therapy is available, under the following circumstances:

Where for compelling and scientifically sound methodological reasons its use is necessary to determine the efficacy or safety of a prophylactic, diagnostic or therapeutic method; or

Where a prophylactic, diagnostic or therapeutic method is being investigated for a minor condition and the patients who receive placebo will not be subject to any additional risk of serious or irreversible harm.

All other provisions of the Declaration of Helsinki must be adhered to, especially the need for appropriate ethical and scientific review.

Note of Clarification on Paragraph 30 of the WMA Declaration of Helsinki

The WMA hereby reaffirms its position that it is necessary during the study planning process to identify post-trial access by study participants to prophylactic, diagnostic and therapeutic procedures identified as beneficial in the study or access to other appropriate care. Post-trial access arrangements or other care must be described in the study protocol so the ethical review committee may consider such arrangements during its review.

♣ ♣ ♣

9.10.2004

The Declaration of Helsinki (Document 17.C) is an official policy document of the World Medical Association, the global representative body for physicians. It was first adopted in 1964 (Helsinki, Finland) and revised in 1975 (Tokyo, Japan), 1983 (Venice, Italy), 1989 (Hong Kong), 1996 (Somerset-West, South Africa) and 2000 (Edinburgh, Scotland). Note of clarification on Paragraph 29 added by the WMA General Assembly, Washington 2002. Note of Clarification on Paragraph 30 added by the WMA General Assembly, Tokyo 2004.

Appendix 2: Statement on insurance policy

GERLING FRANCE

ATTESTATION D'ASSURANCE
N°2005/01/ 28
RESPONSABILITE CIVILE
PROMOTEUR DE RECHERCHE
BIOMEDICALE

Nous soussignés GERLING FRANCE - 111, rue de Longchamp 75116 PARIS, agissant en qualité d'assureur, attestons par la présente que :

Société IRIS
6, place des Pléiades
92400 COURBEVOIE

Agissant tant pour son compte que pour celui des sociétés suivantes :

ARDIX MEDICAL
ASSOCIATION POUR LA NEURO-PSYCHO-PHARMACOLOGIE (A.N.P.P.)
BIOGARAN
BIOPHARMA
EUTHERAPIE
SERVIER MEDICAL
THERVAL MEDICAL

a souscrit un contrat de Responsabilité Civile sous le n° **16/8090722**, conforme aux dispositions légales et réglementaires Françaises sur les recherches biomédicales et notamment aux dispositions de la loi n ° 88.1138 du 20.12.88 modifiée par la loi 90.86 du 23.01.90 et par la loi n° 94.630 du 25 juillet 1994 et les décrets N° 97.888 du 1er octobre 1997 et N° 2002-722 du 3 mai 2002.

Dénomination précise de la recherche assurée :

A one-year multicentre, international, randomised, double-blind study with comparison of benfluorex (150 mg od to 150 mg tid) versus pioglitazone (30 mg od to 45 mg od) in combination with sulfonylurea administered orally for the treatment of type 2 diabetes.

Protocole N° CL3-00780-148

La garantie est conforme à l'obligation d'assurance instituée par les textes de la loi précitée, article L 1121-7 du Code de la Santé Publique,à la charge du promoteur, tant pour sa responsabilité que pour celle des intervenants.

La garantie prévue au contrat restera acquise à l'Assuré en cas de modification affectant la durée du protocole et/ou la prise d'effet du protocole.

La présente attestation vaut présomption de garantie à la charge de l'assureur.

Fait le : 17/10/2005

Marie-France Hurel/Laurence Enizan

GERLING
Allgemeine Versicherungs-AG
Cologne
Capital 224 789 463 EUR
Direction pour la France
111, rue de Longchamp 75116 PARIS

Direction pour la France
111, rue de Longchamp
75116 PARIS
Téléphone : +33 (0) 1 44 05 56 00
Téléfax : +33 (0) 1 44 05 56 66
e-mail : info@gerling.fr
Web : www.gerling.fr

Entreprise privée régie
par le Code des Assurances
Capital 224 789 463 €
R.C.S. Paris B 775 746 480

Siège social : Gerling-Konzern
Allgemeine Versicherungs-AG,
Von-Werth-Strasse. 4-14, D - 50670 Köln
Téléphone : 00 49 221 144 1
Telefax : 00 49 221 144- 33 19

GERLING FRANCE

LIABILITY INSURANCE CERTIFICATE
N°2005/01/ 28
BIOMEDICAL RESEARCH WITH RESPECT TO THE LAW n°88.1138 OF 20/12/1988 and its modifications

We the undersigned GERLING France – 11 rue de Longchamp ; 75016 PARIS – hereby certify that the company :

Société IRIS
6, place des Pléiades
F.92400 COURBEVOIE

Acting on its own behalf as well as on behalf of the following companies :

ARDIX MEDICAL
ASSOCIATION POUR LA NEURO-PSYCHO-PHARMACOLOGIE (A.N.P.P.)
BIOGARAN
BIOPHARMA
EUTHERAPIE
SERVIER MEDICAL
THERVAL MEDICAL

Is insured under the policy n° **16/8090722** for its liability as sponsor or arising from any participating parties, according to article L1121-.7 of the Public Health Code, amended by the law n° 94 630 of July 25th, 1994.

The policy complies with the compulsory insurance requirements of the Decree n° 91.440 of May 14th, 1991, n°97-888 of October 1st, 1997 and n° 2002-722 of May 3rd, 2002.

Title of the insured trial :

A one-year multicentre, international, randomised, double-blind study with comparison of benfluorex (150 mg od to 150 mg tid) versus pioglitazone (30 mg od to 45 mg od) in combination with sulfonylurea administered orally for the treatment of type 2 diabetes.

Protocol N° CL3-00780-148

The above mentioned coverage remains available in the event of a modification of the Protocol's duration or inception date.

The present certificate does not bind the insurer beyond the terms and the limits of the policy it refers to, notably the article L.1121-7 of the Public Health Code.

Date : 17/10/2005

Marie-France Hurel/Laurence Enizan

GERLING
Allgemeine Versicherungs-AG
Cologne
Capital 224 789 463 EUR
Direction pour la France
111, rue de Longchamp 75116 PARIS

Direction pour la France
111, rue de Longchamp
75116 PARIS
Téléphone : +33 (0) 1 44 05 56 00
Téléfax : +33 (0) 1 44 05 56 66
e-mail : info@gerling.fr
Web : www.gerling.fr

Entreprise privée régie
par le Code des Assurances
Capital 224 789 463 €
R.C.S. Paris B 775 746 480

Siège social : Gerling-Konzern
Allgemeine Versicherungs-AG,
Von-Werth-Strasse. 4-14, D - 50670 Köln
Téléphone : 00 49 221 144 1
Telefax : 00 49 221 144- 33 19

AXA CORPORATE SOLUTIONS

LIABILITY INSURANCE CERTIFICATE

AXA Corporate Solutions Assurance, A French "Societé Anonyme" governed by the French Insurance Code, With a share capital of 190,069,080 EUR, Registered with the Trade Register of Paris under number 399 227 354, Registered Office : 4 Rue Jules Lefebvre, 75426 Paris Cedex 09, hereby certifies that the following company :

IRIS
6 place des Pléiades
92415 COURBEVOIE CEDEX

is insured under the Comprehensive General Liability Policy n° **413 030 220 20** of which policyholder is the LABORATOIRES SERVIER, company duly organised in accordance with the french laws.

This policy could cover, where necessary and subject to compliance with any regulations in force, against **IRIS** ' liability at law arising out of sponsorship of clinical trial carried out within the scope of the protocol **CL3-00780-148** entitled as follows and authorised to be performed in **ARGENTINA**.

It is understood that such a coverage shall no substitute for any insurance locally required to be effected in accordance with local rules.

Title of study :	**A one-year multicentre, international, randomised, double-blind study with comparison of benfluorex (150 mg od to 150 mg tid) versus pioglitazone (30 mg od to 45 mg od) in combination with sulfonylurea administered orally for the treatment of type 2 diabetes.**
Protocol N° :	**CL3-00780-148**
Phase of development :	**III**

AMOUNT OF COVERAGE

The above policy provides with the following limit of liability as a maximum in respect of one combined single loss and, where applicable, in the whole aggregate during one period of insurance, such an amount including the following sub-limits, regardless of the number of insureds, of claims made or suits brought.

All combined damage .... EUR 15 000 000 each and every loss and in the aggregate

This certificate is issued as a matter of information only and confers no rights upon the certificate holder. It does not bind the insurer beyond the terms, conditions and limits of the aforementioned original policy and is valid until 30th September 2006, subject to « coverage suspension and/or cancellation » clauses where applicable, as provided for under the aforesaid policy.

This certificate is not valid for risks in any foreign countries where local legislation requires that such an insurance can only be effected with approved insurers operating in the country in question.

Issued in Paris, this 19th day of October, 2005
SIGNED FOR AND ON BEHALF OF
AXA CORPORATE SOLUTIONS ASSURANCE

AXA CORPORATE SOLUTIONS ASSURANCE
Société Anonyme de droit français, régie par le code des Assurances [illegible]
Siège Social : 4, rue Jules Lefebvre
75426 PARIS Cedex 09
Tél. 01 56 92 80 00 - Fax 01 56 92 80 01
www.axa-corporatesolutions.com

AXA CORPORATE SOLUTIONS ASSURANCE
4, Rue Jules Lefebvre 75426 Paris Cedex 09, France. Tél. : +33 1 56 92 80 00. Fax : +33 1 56 92 80 01
Société Anonyme de droit français, régie par le code des Assurances au capital de 190 069 080 €. 399 227 354 RCS Paris
Site Internet : www.axa-corporatesolutions.com

AXA Versicherung AG
Niederlassung Köln

Versicherungsnehmer: IRIS Servier Forschung
6, Place de Pléiades
F-92400 Courbevoie

Police-Nr.: 50235016222

Studie Nr.: **CL3-00780-148**

Studie:
A one-year multicentre, international, randomised, double-blind study with comparison of benfluorex (150 mg o.d. to 150 mg tid) versus pioglitazone (30 mg o.d. to 45 mg o.d.) in combination with sulfonylurea administered orally for the treatment of type 2 diabetes.

Dauer der Studie: 01/2006 bis zum Ablauf der Studie

Versicherungsdauer:

Die vorbezeichnete Probandenversicherung besteht für die gesamte Dauer der im Betreff genannten Studie.

Vertragsgrundlage:

Vertragsgrundlage sind die Allgemeinen Versicherungsbedingungen für die klinischen Prüfungen von Arzneimitteln (Probandenversicherung).

Der Versicherer gewährt Versicherungsschutz für den Fall, daß bei einer von dem Versicherungsnehmer nach den Vorschriften der §§ 40 und 41 des Arzneimittelgesetzes (AMG) durchgeführten oder veranlaßten klinischen Prüfung eines Arzneimittels eine von der Prüfung betroffene Person (Versicherter) getötet oder ihr Körper oder ihre Gesundheit verletzt wird (Gesundheitsschädigung).

Versicherungssummen:

Die Höchstersatzleistung beträgt für alle Versicherungsfälle aus der klinischen Prüfung eines Arzneimittels

10.225.838 EUR, wenn bis zu 1.000 Personen
20.451.675 EUR, wenn mehr als 1.000 bis 3.000 Personen
30.677.512 EUR, wenn mehr als 3.000 Personen
an den klinischen Prüfungen teilnehmen.

Je versicherte Person bilden

511.292 Eur die Höchstgrenze für die Leistungen des Versicherers.

Die Höchstleistung für alle Versicherungsfälle aus den im Versicherungsjahr begonnenen klinischen Prüfungen von Arzneimitteln beträgt

51.129.189 EUR

Köln, den 18.10.2005

AXA Versicherung AG
Niederlassung Köln
Industriedirektion

i. A. [illegible] i. A. [illegible]

CL3-00780-148.doc
Gesellschaftsangaben umseitig

CORPORATE SOLUTIONS

LIABILITY INSURANCE CERTIFICATE

AXA Corporate Solutions Assurance, A French "Societé Anonyme" governed by the French Insurance Code, With a share capital of 190,069,080 EUR, Registered with the Trade Register of Paris under number 399 227 354, Registered Office : 4 Rue Jules Lefebvre, 75426 Paris Cedex 09, hereby certifies that the following company :

IRIS
6 place des Pléiades
92415 COURBEVOIE CEDEX

is insured under the Comprehensive General Liability Policy n° **413 030 220 20** of which policyholder is the LABORATOIRES SERVIER, company duly organised in accordance with the french laws.

This policy could cover, where necessary and subject to compliance with any regulations in force, against **IRIS** ' liability at law arising out of sponsorship of clinical trial carried out within the scope of the protocol **CL3-00780-148** entitled as follows and authorised to be performed in **INDIA**.

It is understood that such a coverage shall no substitute for any insurance locally required to be effected in accordance with local rules.

Title of study : **A one-year multicentre, international, randomised, double-blind study with comparison of benfluorex (150 mg od to 150 mg tid) versus pioglitazone (30 mg od to 45 mg od) in combination with sulfonylurea administered orally for the treatment of type 2 diabetes.**

Protocol N° : **CL3-00780-148**

Phase of development : **III**

AMOUNT OF COVERAGE

The above policy provides with the following limit of liability as a maximum in respect of one combined single loss and, where applicable, in the whole aggregate during one period of insurance, such an amount including the following sub-limits, regardless of the number of insureds, of claims made or suits brought.

All combined damage .... EUR 15 000 000 each and every loss and in the aggregate

This certificate is issued as a matter of information only and confers no rights upon the certificate holder. It does not bind the insurer beyond the terms, conditions and limits of the aforementioned original policy and is valid until 30th September 2006, subject to « coverage suspension and/or cancellation » clauses where applicable, as provided for under the aforesaid policy.

This certificate is not valid for risks in any foreign countries where local legislation requires that such an insurance can only be effected with approved insurers operating in the country in question.

Issued in Paris, this 19th day of October, 2005
SIGNED FOR AND ON BEHALF OF
AXA CORPORATE SOLUTIONS ASSURANCE

AXA CORPORATE SOLUTIONS ASSURANCE
Société Anonyme de droit français, régie par le Code des Assurances
au capital de 190 [illegible] RCS Paris
Siège Social : 4, rue Jules Lefebvre
75426 PARIS Cedex 09
Tél. 01 56 92 80 00 - Fax 01 56 92 80 01
www.axa-corporatesolutions.com

AXA CORPORATE SOLUTIONS ASSURANCE
4, Rue Jules Lefebvre 75426 Paris Cedex 09, France. Tél. : +33 1 56 92 80 00. Fax : +33 1 56 92 80 01
Société Anonyme de droit français, régie par le code des Assurances au capital de 190 069 080 €. 399 227 354 RCS Paris
Site Internet : www.axa-corporatesolutions.com

AXA
CORPORATE
SOLUTIONS

LIABILITY INSURANCE CERTIFICATE

AXA Corporate Solutions Assurance, A French "Societé Anonyme" governed by the French Insurance Code, With a share capital of 190,069,080 EUR, Registered with the Trade Register of Paris under number 399 227 354, Registered Office : 4 Rue Jules Lefebvre, 75426 Paris Cedex 09, hereby certifies that the following company :

IRIS
6 place des Pléiades
92415 COURBEVOIE CEDEX

is insured under the Comprehensive General Liability Policy n° **413 030 220 20** of which policyholder is the LABORATOIRES SERVIER, company duly organised in accordance with the french laws.

This policy could cover, where necessary and subject to compliance with any regulations in force, against **IRIS** ' liability at law arising out of sponsorship of clinical trial carried out within the scope of the protocol **CL3-00780-148** entitled as follows and authorised to be performed in **SOUTH AFRICA**.

It is understood that such a coverage shall no substitute for any insurance locally required to be effected in accordance with local rules.

Title of study :	**A one-year multicentre, international, randomised, double-blind study with comparison of benfluorex (150 mg od to 150 mg tid) versus pioglitazone (30 mg od to 45 mg od) in combination with sulfonylurea administered orally for the treatment of type 2 diabetes.**
Protocol N° :	**CL3-00780-148**
Phase of development :	**III**

AMOUNT OF COVERAGE

The above policy provides with the following limit of liability as a maximum in respect of one combined single loss and, where applicable, in the whole aggregate during one period of insurance, such an amount including the following sub-limits, regardless of the number of insureds, of claims made or suits brought.

All combined damage .... EUR 15 000 000 each and every loss and in the aggregate

The above mentioned policy applies as an excess layer upon the policy **n° 394/99009610199/6** effected by **SERVIER LABORATORIES S.A. PTY** acting on behalf of IRIS as well, being agreed that the liability of the Insurer shall not exceed the above amount of coverage, all policies combined including the local policy **n° 394/99009610199/6**.

This certificate is issued as a matter of information only and confers no rights upon the certificate holder. It does not bind the insurer beyond the terms, conditions and limits of the aforementioned original policy and is valid until 30th September 2006, subject to « coverage suspension and/or cancellation » clauses where applicable, as provided for under the aforesaid policy.

This certificate is not valid for risks in any foreign countries where local legislation requires that such an insurance can only be effected with approved insurers operating in the country in question.

Issued in Paris, this 19th day of October, 2005
SIGNED FOR AND ON BEHALF OF
AXA CORPORATE SOLUTIONS ASSURANCE

AXA CORPORATE SOLUTIONS ASSURANCE
Société Anonyme de droit français, régie par le Code des Assurances
au capital de 190 069 080 euros - 399 227 354 RCS Paris
Siège Social : [illegible]
75426 PARIS Cedex 09
Tél. 01 56 92 80 00 - Fax 01 56 92 80 01
www.axa-corporatesolutions.com

AXA CORPORATE SOLUTIONS ASSURANCE
4, Rue Jules Lefebvre 75426 Paris Cedex 09, France. Tél. : +33 1 56 92 80 00. Fax : +33 1 56 92 80 01
Société Anonyme de droit français, régie par le code des Assurances au capital de 190 069 080 €. 399 227 354 RCS Paris
Site Internet : www.axa-corporatesolutions.com

Appendix 3: General Policy for handling study data

Responsibility for the content of scientific presentations and publications lies with the authors, eg Scientic Committee members, coordinators, investigators and clinical scientists from Servier.

I. Publications Committee

A. Purpose

1. To ensure the accuracy and high quality of publications and presentations representing the CL3-00780-148 Study effort, serving as a "pre-peer review". This is to facilitate rather than hinder publication.

2. To promote the publication and presentation of data arising from the effort.

3. To monitor and adjudicate disputes over autorship or duplication of effort. This is primarily a preventive function.

B. Membership

1. Members will be all members of Scientific Committee from a subset of investigators and one clinical scientist from Servier.

2. The Chairman of the Publications Committee will be the chairman of the Scientific Committee.

C. Data Access

The CRO in charge of data management will provide to I.R.I.S. the cleaned and locked database and initial analysis of the pooled data. The chairman of the Publication Committee will adise the Publication Committee on the range of analyses the collected data will support.

1. Definitions

a) Pooled data is data from more than one site. This data is the property of I.R.I.S.. Decisions as to modes of data analysis will be made by I.R.I.S. in consultation with Scientific Committee.

b) Site data refers to data collected within an individual site. It may be analyzed collectively and published in the appropriate national journals and presented at appropriate national meetings.

2. Data Analysis

a) Analysis of pooled data will be performed by CRO in charge of biostatistics consultation with the Data

Management Center and the Publication Committee members.

b) Suggestions for data analysis of pooled data should be directed, prior to database lock, to Publication Committee members.

3. Access of Data

The data collected from the CL3-00780-148 Study will be the property of I.R.I.S. All members of the Publications Committee will be provided with a complete data file of all pooled data set after the completion of data collection and cleaning of the data.

II. Publications Policies

1. Responsabilities of Committee

a) The Committee will pre-approve or deny all requests for data access, secondary analyses, publications and presentations deriving from pooled data in advance of the analysis or writing of the paper. The purpose of this function is to prevent inadvertent duplication of effort.

b) The Committee will make such decisions in a timely manner (i.e. within 30 days).

c) The Committee will coordinate requests and provide all investigators an opportunity to express interest in participating and collaborating in projects.

d) The Committee will review and comment on all manuscripts and presentations arising from CL3-00780-148 Study pooled data.

e) Disputes over order of autorship, data access, or other matters related to publication and dissemination of the CL3-00780-148 Study finding will be decided by unanimous vote of the Publication Committee.

f) With the exceptions of papers arising from site data not directly related to the study objectives, no papers will be submitted for publication until the major study objectives are submitted for publication.

g) The Committee will submit the protocol to potential review in accordance with their own rules of publication.

2. Responsablities of Authors

a) Authors will submit all manuscripts or proposed presentations to the Committee for review and comment, including pooled or site data.

b) The author writing a paper will be first author, unless otherwise agreed upon in advance.

c) While order of authorship may be proposed in advance, the final order of authorship must be based upon the relative intellectual contribution each author had, including the design, implementation, analysis, or final write-up of the paper. All authors must meet all four of the criteria proposed by the International Committee of Medical Journal Editors. Specifically, each author must:

1. Make substantial contributions either to the "conception and design" of the work or to "analysis and interpretation of the data."

2. Make substantial contributions to drafting the article or revising it critically for important intellectual content.

3. Give approval to the final version of the manuscript.

4. Take public responsibility for the content of the manuscript.

d) A standard template of the original participants of the study will be listed in alphabetical order in the acknowledgements of each publication arising from pooled data of involved sites.

Appendix 4: WHO criteria of diabetes mellitus

Screening for Type 2 Diabetes: "Report of World Health Organisation and International Diabetes Federation meeting. Geneva, World Health Organization, 2003".

World Health Organisation
Department of Noncommunicable Disease Management
Geneva
WHO/NMH/MNC/03.1

Biochemical criteria (venous plasma) for the diagnosis of diabetes mellitus

	**Glucose concentration, mmol/L (mg/dL) (Venous plasma)***
**Diabetes mellitus:**	
Fasting plasma glucose **and/or**	≥ 7.0 (≥ 126)
Casual plasma glucose **and/or**	≥ 11.1 (≥ 200)
Plasma glucose value 2 hours after a 75g oral glucose load	≥ 11.1 (≥ 200)

*In asymptomatic subjects, performing the test on one occasion is not enough to establish the diagnosis (i.e. basis to treat diabetes). This must be confirmed by carrying out at least one further test on a subsequent day.

Appendix 5: Summary of product characteristics of Actos® (Pioglitazone)

1. NAME OF THE MEDICINAL PRODUCT

ACTOS 15 mg or 30 mg or 45 mg, tablets.

2. QUALITATIVE AND QUANTITATIVE COMPOSITION

Each tablet contains 15 mg, 30 mg or 45 mg of pioglitazone as hydrochloride.

For excipients, see 6.1.

3. PHARMACEUTICAL FORM

Tablet.

The tablets are white to off-white, round, convex (ACTOS® 15 mg) or flat (ACTOS® 30 mg and 45 mg) and marked '15', '30' or '45' on one face.

4. CLINICAL PARTICULARS

4.1 Therapeutic indications

Pioglitazone is indicated as oral monotherapy in type 2 diabetes mellitus patients, particularly overweight patients, inadequately controlled by diet and exercise for whom metformin is inappropriate because of contraindications or intolerance.

Pioglitazone is also indicated for oral combination treatment in type 2 diabetes mellitus patients with insufficient glycaemic control despite maximal tolerated dose of oral monotherapy with either metformin or sulphonylurea:

- in combination with metformin particularly in overweight patients
- in combination with a sulphonylurea only in patients who show intolerance to metformin or for whom metformin is contraindicated

4.2 Posology and method of administration

The long-term benefits of therapy with pioglitazone have not been demonstrated (see section 5.1).

Pioglitazone tablets are taken orally once daily with or without food.

Dosage in adults:
Pioglitazone may be initiated at 15mg or 30mg once daily. The dose may be increased in increments up to 45mg once daily.
In combination with metformin, the current metformin dose can be continued upon initiation of pioglitazone therapy.

In combination with sulphonylurea, the current sulphonylurea dose can be continued upon initiation of pioglitazone therapy. If patients report hypoglycaemia, the dose of sulphonylurea should be decreased.

Elderly:
No dosage adjustment is necessary for elderly patients (see section 5.2).

Patients with renal impairment:
No dosage adjustment is necessary in patients with impaired renal function (creatinine clearance < 40 mL/min) (see section 5.2). No information is available from dialysed patients therefore pioglitazone should not be used in such patients.

Patients with hepatic impairment:
Pioglitazone should not be used in patients with hepatic impairment (see section 4.4).

Children and adolescents:
There are no data available on the use of pioglitazone in patients under 18 years of age, and therefore its use is not recommended in this age group.

4.3 Contraindications

Pioglitazone is contraindicated in patients with:
- known hypersensitivity to pioglitazone or to any of the excipients of the tablet
- cardiac failure or history of cardiac failure (NYHA stages I to IV)
- hepatic impairment.

Pioglitazone is also contraindicated for use in combination with insulin.

4.4 Special warnings and special precautions for use

There is no clinical experience with pioglitazone in triple combination with other oral antidiabetics.

Fluid retention and cardiac failure:
Pioglitazone can cause fluid retention, which may exacerbate or precipitate heart failure. Patients should be observed for signs and symptoms of heart failure, particularly those with reduced cardiac reserve. Pioglitazone should be discontinued if any deterioration in cardiac status occurs. There have been cases of cardiac failure reported from the market when pioglitazone was used in combination with insulin. Therefore pioglitazone is contraindicated in combination with insulin. There also have been cases of cardiac failure reported from the market when pioglitazone was used in patients with a history of cardiac failure. Since NSAIDs and pioglitazone are associated with fluid retention, concomitant administration may increase the risk of oedema.

Monitoring of liver function:
There have been rare reports of hepatocellular dysfunction during post-marketing experience (see section 4.8). It is recommended, therefore, that patients treated with pioglitazone undergo periodic monitoring of liver enzymes. Liver enzymes should be checked prior to the initiation of therapy with pioglitazone in all patients. Therapy with pioglitazone should not be initiated

in patients with increased baseline liver enzyme levels (ALT > 2.5 X upper limit of normal) or with any other evidence of liver disease.

Following initiation of therapy with pioglitazone, it is recommended that liver enzymes be monitored periodically based on clinical judgement. If ALT levels are increased to 3 X upper limit of normal during pioglitazone therapy, liver enzyme levels should be reassessed as soon as possible. If ALT levels remain > 3 X the upper limit of normal, therapy should be discontinued. If any patient develops symptoms suggesting hepatic dysfunction, which may include unexplained nausea, vomiting, abdominal pain, fatigue, anorexia and/or dark urine, liver enzymes should be checked. The decision whether to continue the patient on therapy with pioglitazone should be guided by clinical judgement pending laboratory evaluations. If jaundice is observed, drug therapy should be discontinued.

Weight gain:
In clinical trials with pioglitazone there was evidence of weight gain, therefore weight should be closely monitored. Part of the treatment of diabetes is dietary control. Patients should be advised to adhere strictly to a calorie-controlled diet.

Haematology:
There was a small reduction in mean haemoglobin (4 % relative reduction) and haematocrit (4.1 % relative reduction) during therapy with pioglitazone, consistent with haemodilution. Similar changes were seen in metformin (haemoglobin 3 - 4 % and haematocrit 3.6 – 4.1 % relative reductions) and to a lesser extent sulphonylurea (haemoglobin 1 – 2 % and haematocrit 1 – 3.2 % relative reductions) treated patients in comparative controlled trials with pioglitazone.

Others:
As a consequence of enhancing insulin action, pioglitazone treatment in patients with polycystic ovarian syndrome may result in resumption of ovulation. These patients may be at risk of pregnancy.

Patients should be aware of the risk of pregnancy and if a patient wishes to become pregnant or if pregnancy occurs, the treatment should be discontinued (see section 4.6).

4.5 Interaction with other medicinal products and other forms of interaction

Interaction studies have shown that pioglitazone has no relevant effect on either the pharmacokinetics or pharmacodynamics of digoxin, warfarin, phenprocoumon and metformin. Co-administration of pioglitazone with sulphonylureas does not appear to affect the pharmacokinetics of the sulphonylurea. Studies in man suggest no induction of the main inducible cytochrome P450, 1A, 2C8/9 and 3A4. In vitro studies have shown no inhibition of any subtype of cytochrome P450. Interactions with substances metabolised by these enzymes, e.g. oral contraceptives, cyclosporin, calcium channel blockers, and HMGCoA reductase inhibitors are not to be expected.

4.6 Pregnancy and lactation

Use in pregnancy:
There are no adequate human data to determine the safety of pioglitazone during pregnancy. Foetal growth restriction was apparent in animal studies with pioglitazone. This was attributable to the action of pioglitazone in diminishing the maternal hyperinsulinaemia and increased insulin resistance that occurs during pregnancy thereby reducing the availability of metabolic substrates for foetal growth. The relevance of such a mechanism in humans is unclear and pioglitazone should not be used in pregnancy.

Use in breast-feeding:
Pioglitazone has been shown to be present in the milk of lactating rats. It is not known whether pioglitazone is secreted in human milk. Therefore, pioglitazone should not be administered to breast-feeding women.

4.7 Effects on ability to drive and use machines

No effects on ability to drive and use machines have been observed.

4.8 Undesirable effects
Adverse reactions reported in excess (> 0.5 %) of placebo and as more than an isolated case in patients receiving pioglitazone in double-blind studies are listed below as MedDRA preferred term by system organ class and absolute frequency. Frequencies are defined as: common > 1/100, < 1/10; uncommon > 1/1000, < 1/100; rare > 1/10000, < 1/1000; very rare < 1/10000.

Monotherapy:
- Eye disorders
  Common: visual disturbance
- Infection and infestations
  Common: upper respiratory tract infection
  Uncommon: sinusitis
- Investigations
  Common: weight increased
- Nervous system disorders
  Common: hypoaesthesia
  Uncommon: insomnia

Pioglitazone in combination with metformin:
- Blood and lymphatic system disorders
  Common: anaemia
- Eye disorders
  Common: visual disturbance
- Gastrointestinal disorders
  Uncommon: flatulence
- Investigations
  Common: weight increased
- Musculoskeletal system and connective tissue disorders
  Common: arthralgia
- Nervous system disorders
  Common: headache
- Renal and urinary disorders

Common: haematuria
- Reproductive system and breast disorders
  Common: erectile dysfunction

Pioglitazone in combination with sulfonylurea:
- Ear and labyrinth disorders
  Uncommon: vertigo
- Eye disorders
  Uncommon: visual disturbance
- Gastrointestinal disorders
  Common: flatulence
- General disorders and administration site conditions
  Uncommon: fatigue
- Investigations
  Common: weight increased
  Uncommon: increased lactic dehydrogenase
- Metabolism and nutritional disorders
  Uncommon: appetite increased, hypoglycaemia
- Nervous system disorders
  Common: dizziness
  Uncommon: headache
- Renal and urinary disorders
  Uncommon: glycosuria, proteinuria
- Skin and subcutaneous tissue disorders
  Uncommon: sweating

Oedema was reported in 6 – 9 % of patients treated with pioglitazone over one year in controlled clinical trials. The oedema rates for comparator groups (sulphonylurea, metformin) were 2 – 5 %. The reports of oedema were generally mild to moderate and usually did not require discontinuation of treatment.

In active comparator controlled trials mean weight increase with pioglitazone given as monotherapy was 2 – 3 kg over one year. This is similar to that seen in a sulphonylurea active comparator group. In combination trials pioglitazone added to metformin resulted in mean weight increase over one year of 1.5 kg and added to a sulphonylurea of 2.8 kg. In comparator groups addition of sulphonylurea to metformin resulted in a mean weight gain of 1.3 kg and addition of metformin to a sulphonylurea a mean weight loss of 1.0 kg.

Visual disturbance has been reported mainly early in treatment and is related to changes in blood glucose due to temporary alteration in the turgidity and refractive index of the lens as seen with other hypoglycaemic agents.

In clinical trials with pioglitazone the incidence of elevations of ALT greater than three times the upper limit of normal was equal to placebo but less than that seen in metformin or sulphonylurea comparator groups. Mean levels of liver enzymes decreased with treatment with pioglitazone. Rare cases of elevated liver enzymes and hepatocellular dysfunction have occurred in post-marketing experience. Although in very rare cases fatal outcome has been reported, causal relationship has not been established.

In controlled clinical trials the incidence of reports of heart failure with pioglitazone treatment was the same as in placebo, metformin and sulphonylurea treatment groups. Heart failure has been reported rarely with marketing use of pioglitazone.

4.9 Overdose

Patients have taken pioglitazone at higher than the recommended highest dose of 45 mg daily. The maximum reported dose of 120 mg/day for four days, then 180 mg/day for seven days was not associated with any symptoms.

Hypoglycaemia may occur in combination with sulphonylureas or insulin. Symptomatic and general supportive measures should be taken in case of overdose.

5. PHARMACOLOGICAL PROPERTIES

5.1 Pharmacodynamic properties

Pharmacotherapeutic group: oral blood glucose lowering drugs; Thiazolidinediones;
ATC code: A10 BG 03.

Pioglitazone effects may be mediated by a reduction of insulin resistance. Pioglitazone appears to act via activation of specific nuclear receptors (peroxisome proliferator activated receptor gamma) leading to increased insulin sensitivity of liver, fat and skeletal muscle cells in animals. Treatment with pioglitazone has been shown to reduce hepatic glucose output and to increase peripheral glucose disposal in the case of insulin resistance.

Fasting and postprandial glycaemic control is improved in patients with type 2 diabetes mellitus. The improved glycaemic control is associated with a reduction in both fasting and postprandial plasma insulin concentrations. A clinical trial of pioglitazone vs. gliclazide as monotherapy was extended to two years in order to assess time to treatment failure (defined as appearance of HbA1c ¡Ý8.0 % after the first six months of therapy). Kaplan-Meier analysis showed shorter time to treatment failure in patients treated with gliclazide, compared with pioglitazone. At two years, glycaemic control (defined as HbA1c < 8.0 %) was sustained in 69 % of patients treated with pioglitazone, compared with 50 % of patients on gliclazide. In a two-year study of combination therapy comparing pioglitazone with gliclazide when added to metformin, glycaemic control measured as mean change from baseline in HbA1c was similar between treatment groups after one year. The rate of deterioration of HbA1c during the second year was less with pioglitazone than with gliclazide.

HOMA analysis shows that pioglitazone improves beta cell function as well as increasing insulin sensitivity. Two-year clinical studies have shown maintenance of this effect.

In one year clinical trials, pioglitazone consistently gave a statistically significant reduction in the albumin/creatinine ratio compared to baseline.

The effect of pioglitazone (45 mg monotherapy vs. placebo) was studied in a small 18-week trial in type 2 diabetics. Pioglitazone was associated with significant weight gain. Visceral fat was significantly decreased, while there was an increase in extra-abdominal fat mass. Similar changes in body fat distribution on pioglitazone have been accompanied by an improvement

in insulin sensitivity. In most clinical trials, reduced total plasma triglycerides and free fatty acids, and increased HDL cholesterol levels were observed as compared to placebo, with no statistically significant increases in LDL cholesterol levels. In clinical trials of up to two years duration, pioglitazone reduced total plasma triglycerides and free fatty acids, and increased HDL cholesterol levels, compared with placebo, metformin or gliclazide. Pioglitazone did not cause statistically significant increases in LDL cholesterol levels compared with placebo, whilst reductions were observed with metformin and gliclazide. In a 20-week study, as well as reducing fasting triglycerides, pioglitazone reduced postprandial hypertriglyceridaemia through an effect on both absorbed and hepatically synthesised triglycerides. These effects were independent of pioglitazone's effects on glycaemia and were statistically significant different to glibenclamide.

An outcome study is underway with pioglitazone, and until this is completed the long-term benefits associated with improved metabolic control have not been demonstrated.

5.2 Pharmacokinetic properties

Absorption:
Following oral administration, pioglitazone is rapidly absorbed, and peak plasma concentrations of unchanged pioglitazone are usually achieved 2 hours after administration. Proportional increases of the plasma concentration were observed for doses from 2 – 60 mg. Steady state is achieved after 4– 7 days of dosing. Repeated dosing does not result in accumulation of the compound or metabolites. Absorption is not influenced by food intake. Absolute bioavailability is greater than 80 %.

Distribution:
The estimated volume of distribution in humans is 0.25 l/kg.

Pioglitazone and all active metabolites are extensively bound to plasma protein (> 99 %).

Metabolism:
Pioglitazone undergoes extensive hepatic metabolism by hydroxylation of aliphatic methylene groups. This is predominantly via cytochrome P450 3A4 and 2C9 although multiple other isoforms are involved to a lesser degree. Three of the six identified metabolites are active (M-II, M-III, and M-IV). When activity, concentrations and protein binding are taken into account, pioglitazone and metabolite M-III contribute equally to efficacy. On this basis M-IV contribution to efficacy is approximately three-fold that of pioglitazone, whilst the relative efficacy of M-II is minimal.
In vitro studies have shown no evidence that pioglitazone inhibits any subtype of cytochrome P450. There is no induction of the main inducible P450 isoenzymes 1A, 2C8/9, and 3A4 in man.

Interaction studies have shown that pioglitazone has no relevant effect on either the pharmacokinetics or pharmacodynamics of digoxin, warfarin, phenprocoumon and metformin. It is therefore not expected that inducers or inhibitors of P450 isoenzymes will alter pioglitazone or active metabolites in a significant way.

Elimination:
Following oral administration of radiolabelled pioglitazone to man, recovered label was mainly in faeces (55%) and a lesser amount in urine (45 %). In animals, only a small amount of unchanged pioglitazone can be detected in either urine or faeces. The mean plasma elimination half-life of unchanged pioglitazone in man is 5 to 6 hours and for its total active metabolites 16 to 23 hours.

Elderly:
Steady state pharmacokinetics are similar in patients age 65 and over and young subjects.

Patients with renal impairment:
In patients with renal impairment, plasma concentrations of pioglitazone and its metabolites are lower than those seen in subjects with normal renal function, but oral clearance of parent substance is similar. Thus free (unbound) pioglitazone concentration is unchanged.

Patients with hepatic impairment:
Total plasma concentration of pioglitazone is unchanged, but with an increased volume of distribution. Intrinsic clearance is therefore reduced, coupled with a higher unbound fraction of pioglitazone.

5.3 Preclinical safety data

In toxicology studies, plasma volume expansion with haemodilution, anaemia, and reversible eccentric cardiac hypertrophy was consistently apparent after repeated dosing of mice, rats, dogs, and monkeys. In addition, increased fatty deposition and infiltration were observed. These findings were observed across species at plasma concentrations ≤ 4 times the clinical exposure. Foetal growth restriction was apparent in animal studies with pioglitazone. This was attributable to the action of pioglitazone in diminishing the maternal hyperinsulinaemia and increased insulin resistance that occurs during pregnancy thereby reducing the availability of metabolic substrates for foetal growth.

Pioglitazone was devoid of genotoxic potential in a comprehensive battery of in vivo and in vitro genotoxicity assays. An increased incidence of hyperplasia (males and females) and tumours (males) of the urinary bladder epithelium was apparent in rats treated with pioglitazone for up to 2 years. The relevance of this finding is unknown. There was no tumorigenic response in mice of either sex. Hyperplasia of the urinary bladder was not seen in dogs or monkeys treated for up to 12 months.

In an animal model of familial adenomatous polyposis (FAP), treatment with two other thiazolidinediones increased tumour multiplicity in the colon. The relevance of this finding is unknown.

6. PHARMACEUTICAL PARTICULARS

6.1 List of excipients

Carmellose calcium, hydroxypropylcellulose, lactose monohydrate and magnesium stearate.

6.2 Incompatibilities

Not applicable.

6.3 Shelf life

3 years.

6.4 Special precautions for storage

No special precautions for storage.

6.5 Nature and contents of container

Aluminium/aluminium blisters, packs of 14, 28, 50, 56 and 98 tablets.

6.6 Instructions for use and handling

No special requirements.

7. MARKETING AUTHORISATION HOLDER

TAKEDA EUROPE R & D CENTRE LIMITED
Savannah House
11-12 Charles II Street
London
SW1Y 4QU
United Kingdom

8. NUMBER(S) IN THE COMMUNITY REGISTER OF MEDICINAL PRODUCTS

EU/1/00/150/001-003, 007, 009

9. DATE OF FIRST AUTHORISATION/RENEWAL OF THE AUTHORISATION

13-10-2000

10. DATE OF REVISION OF THE TEXT: 1srt FEBRUARY 2005

Appendix 6: Summary of product characteristics of Mediator® (Benfluorex)

1. NAME OF THE MEDICINAL PRODUCT

MEDIATOR 150 mg, coated tablet

2. QUALITATIVE AND QUANTITATIVE COMPOSITION

Benfluorex hydrochloride .... 150.00 mg
per coated tablet of 700 mg

For excipients, cf. section 6.1.

3. PHARMACEUTICAL FORM

Coated tablet.

4. CLINICAL DATA

4.1 Therapeutic indications

- Dietary adjuvant suitable for hypertriglyceridemia,
- Dietary adjuvant suitable for overweight diabetics.

Note: efficacy in the primary and secondary prevention of atherosclerotic complications has not been demonstrated.

4.2 Posology and method of administration

Oral route.

The dosage is usually 3 tablets daily. That dosage may be prescribed as of treatment initiation or achieved gradually in the following manner:
- 1st week: 1 tablet daily during dinner,
- 2nd week: 2 tablets daily: 1 during lunch and 1 during dinner,
- from the 3rd week: 3 tablets daily: 1 during breakfast, 1 during lunch and 1 during dinner.

On the basis of the laboratory results, the dosage may be decreased to 2 tablets daily or even 1 tablet daily.

In combination with an appropriate diet, Mediator constitutes an adjuvant treatment. Regular clinical and laboratory monitoring of each patient is to be instituted.

4.3 Contraindications

- Hypersensitivity to benfluorex hydrochloride or one of its constituents
- Established chronic pancreatitis.

4.4 Special warnings and special precautions for use

The metabolic disorders in patients treated with Mediator are mainly observed in adults. Prescription of Mediator for children is therefore unjustified.

If, after a treatment period of a few months (3 to 6 months), a satisfactory decrease in serum lipid or glucose concentration is not obtained, complementary or alternative therapeutic strategies are to be envisaged.

The attention of sportsmen and athletes is drawn to the fact that this proprietary medicinal product contains an active substance that may induce a positive reaction in illicit drug screens.

Due to the presence of sucrose, this medicinal product is not to be used in cases of fructose intolerance, glucose and galactose malabsorption syndrome or sucrase-isomaltase deficiency.

4.5 Interactions with other medicinal products and other forms of interaction

4.6 Pregnancy and lactation

Use in pregnancy:
The animal studies have not shown a teratogenic effect. In the absence of a teratogenic effect in animals, a malformative effect in humans is not expected. To date, the substances responsible for malformations in humans have been shown to be teratogenic in animals in the course of rigorous studies in 2 species.

In clinical practice, no sufficiently pertinent data enabling evaluation of a potential malformative or fetotoxic effect of benfluorex administered during pregnancy are available.

In consequence, as a precaution, Mediator should not be taken during pregnancy. In the event of fortuitous exposure, discontinue treatment.

Use in breast-feeding:
In the absence of data on benfluorex secretion in breast milk, this medicinal product is not advised during breast-feeding.

4.7 Effects on the ability to drive and use machines

The attention of drivers is drawn to the risk of drowsiness associated with use of this medicinal product.

4.8 Undesirable effects

The following adverse reactions were observed: gastrointestinal disorders (nausea, vomiting, stomach pain, diarrhea), asthenia, confusion, drowsiness or dizziness. These effects are

mainly observed at dosages higher than 3 tablets per day. Individual susceptibility has also been observed.

Very rare cases of **anaphylactic** reactions have been reported: hypotension, shock, rash, urticaria, Quincke oedema.

Increase in hepatic enzymes and hepatitis (very rare) have also been reported.

4.9 Overdose

In the event of massive overdose, treatment will be symptomatic only: gastric lavage, osmotic diuresis, correction of any electrolyte disorders, monitoring of blood pressure, state of consciousness, and respiratory and cardiac function.

5. PHARMACOLOGICAL PROPERTIES

5.1 Pharmacodynamic properties

Pharmacotherapeutic group: Cholesterol- and Triglycerides-lowering agents
ATC code: C10AX04

Actions of Mediator on lipid metabolism:
In animals (rat), Mediator decreases the intestinal absorption of triglycerides.
The same effect has been observed in man in clinical pharmacology studies and is reported to be due to a reduction in pancreatic lipase activity.

The following effects have also been observed in animals:
- decrease in the hepatic synthesis of triglycerides and cholesterol, *in vitro* and *in vivo* (rat),
- decrease in the fatty degeneration of the liver induced by lipid-rich and carbohydrate-rich diets (obese rat), and in experimental diabetes (rat),
- reduction in cholesterol incorporation in artery walls (rabbit).

Those mechanisms may partially explain the cholesterol and triglyceride lowering observed in man.

Actions of Mediator on carbohydrate metabolism:
In animals, the following effects have been observed:
- facilitation of glucose penetration into and use by cells (rat)
- reduction in hyperglycemia in the diabetic rat (insulin-dependent or not), decrease in hyperglycemia (measured by the glucose tolerance test area) in the rabbit.

Mediator has no action on insulin secretion. The occurrence of hypoglycemia is improbable.

Complementary effect of Mediator:
A reduction in blood uric acid of about 14% has been observed in hyperuricemic obese patients receiving Mediator in combination with an appropriate diet.

5.2 Pharmacokinetic properties

Gastrointestinal absorption is rapid and total.

Following oral administration, the peak plasma concentration occurs between 1 and 2 hours post-dosing.

Elimination is rapid and total by the urinary route. Eight hours post-dosing, 74% of the dose administered has been eliminated in the urine.

The elimination occurs in 2 phases:
- an initial fast phase (60% in 3 to 4 hours)
- a second slow phase lasting about 36 hours.

5.3 Preclinical safety data

Not applicable.

6. PHARMACEUTICAL PARTICULARS

6.1 List of excipients

Maize starch, sodium bicarbonate, croscarmellose sodium, white beeswax, titanium dioxide (E 171), ethylcellulose, glycerol monooleate, polysorbate 80, povidone, sucrose, anhydrous colloidal silica, magnesium stearate, talc.

6.2 Incompatibility

Not applicable.

6.3 Shelf-life

3 years.

6.4 Special precautions for storage

There are no special precautions for storage.

6.5 Nature and contents of container

Heat-formed blister packs (PVC plus aluminium foil).

6.6 Instructions for use and handling

(cf. 4.2 Posology and method of administration).

7. MARKETING AUTHORIZATION HOLDER

LES LABORATOIRES SERVIER
22 rue Garnier
92200 NEUILLY SUR SEINE Cedex

8. NUMBER(S) IN THE COMMUNITY REGISTER OF MEDICINAL PRODUCTS

France: 317 557.9 (30 tablets)
317 559.1 (100 tablets)

9. DATE OF FIRST AUTHORISATION/RENEWAL OF THE AUTHORISATION

16-07-1974
modification: 22-04-1987

10. DATE OF REVISION OF THE TEXT

02-10-2002

Appendix 7: Concomitant medications

DRUGS NOT ALLOWED

- Any sulfonylurea other than the one received at selection, and to be prescribed throughout the study at the same dosage unless a decrease in the sulfonylurea dose is judged necessary by the investigator (e.g. severe or multiple hypoglycaemic episode).
- Short acting insulin secretagogues (such as repaglinide or nateglinide).
- Insulin (contraindicated with pioglitazone).
- Biguanides (such as metformin).
- α-glucosidase inhibitors.
- Other thiazolidinedione (Rosaglitazone).
- Drugs contraindicated with sulfonylureas: Miconazole – systemic route, oral gel – is an absolute contraindication (please refer to the Summary of Product Characteristics of the prescribed sulfonylurea).
- Oral injected corticosteroids in chronic use (see "Drugs allowed for short term use in exceptional cases" below).

DRUGS ONLY ALLOWED IN CHRONIC USE

- Usual concomitant medications are authorised if resulting in a well controlled and stabilised pathology. Their dosage should remain stable throughout the study as far as possible
- Weight lowering agents and lipid lowering agents are authorised only if they have been taken for at least 3 months before selection and can be taken throughout the study at stable daily dose. Starting lipid lowering drugs is allowed from W8.

DRUGS ONLY ALLOWED FOR SHORT TERM USE IN EXCEPTIONAL CASES

Oral and injected corticosteroids are accepted only if needed for a duration of less than 10 days and at a distance of at least one month from the forthcoming evaluation visit. Oral and injected corticosteroids are not allowed if chronically used.
Insulin is allowed for short therm treatment (< 7 days).

SPECIAL WARNINGS AND SPECIAL PRECAUTIONS FOR USE

NSAIDs: Since their combination with Pioglitazone is associated with fluid retention, their use should be considered with the greatest precautions.

Appendix 8: Hypoglycaemia

1) Patient information

The investigator will inform the patient on trigger factors of hypoglycaemia, and on symptoms suggestive of hypoglycaemia.

A list of symptoms indicative of hypoglycaemia is given in table 1. A symptomatic hypoglycaemic episode could also be suspected on the acute onset of unusual symptoms on the judgement of the investigator.

Table 1: Symptoms indicative of hypoglycaemia

❑ Confusion	❑ Difficulty concentrating
❑ Sweating	❑ Unsteadiness
❑ Unexplained behaviour or mood change	❑ Visual disturbance
❑ Drowsiness	❑ Hunger
❑ Weakness	❑ Pallor
❑ Lightheadedness	❑ Nausea
❑ Dizziness	❑ Incoordination,
❑ Difficulty speaking	❑ Tremor
❑ Pounding heart	❑ Headache

Table 2: Trigger factors of hypoglycaemia

□ missed meal or meal without carbohydrates,
□ unusual physical exercise,
□ absorption of alcohol without food,
□ new concomitant treatment
□ other

2) Management of hypoglycaemia

By the patient:
Hypoglycaemia must be immediately treated by sufficient sugar intake, i.e. equivalent to 10 g of glucose per os or 9 g of glucose IV.

The patient should be encouraged to report any severe and/or multiple hypoglycaemic episodes to the investigator, even outside planned study visits.

He/she should be encouraged to fill in his/her diary card with as many details as possible, particularly:
- Date and time of onset
- Description of the symptoms
- Management of the episode (sugar intake, other…)
- Duration of symptoms after sugar intake

By the investigator:
The symptomatic hypoglycaemic episodes are considered:
- Mild: if the symptoms did not interfere with usual activities,
- Moderate: if the symptoms transiently interfered with usual activities,
- Severe: defined as symptomatic episode requiring external assistance due to severe impairment in consciousness or behavior.

In case of severe or multiple (> 3 episodes per month) hypoglycaemic episodes, an adjustment in the antidiabetic treatment may be judged necessary by the investigator: the sulfonylurea dose can be decreased and in the same time glucose or glucagon quickly administrated.

In all cases, the investigator will reinforce advice or hypoglycaemia and particularly triggers factors to be avoided.

3) Reporting of hypoglycaemia

In the case report form, the investigator will fill in the specific form "Record of suspected hypoglycaemia" in the CRF:
- date/time of onset,
- measures taken by the patient,
- duration of symptoms after sugar intake,
- severity of the episode (see 2),
- any adjustment in the antidiabetic treatment (see 2).

This form should only be filled in for episodes suggestive of hypoglycaemia. All the other adverse events should be reported in the Adverse Events Form.

In case of severe hypoglycaemic episode, the investigator will also follow the procedure for serious adverse events described in section 12.3.1.3.

Appendix 9: Blood sampling in case of overdosage

Sample treatment

2 mL blood samples will be collected in dry tubes.

The blood will be **immediately** separated into plasma by centrifugation (10 mn, at 3500 rpm at + 4° C) and will be **immediately** stabilized by adding sodium fluoride (50 µL of a 40 mg/mL solution/mL plasma).

Plasma will be separated and divided into 2 aliquots in 2 tubes for determination of S 780 and its metabolites (aliquots n°1 and n°2). Each aliquot will be clearly identified with the following information: protocol number, centre number, patient identification, birth date, time of sampling, aliquot number (1 or 2).

Each sample will then be rapidly deep frozen and stored at – 20°C. These samples can be stored at-20°C for a maximum of one week before transfer to the central laboratory.

Samples transfer

Aliquot n°1 and n°2 will be sent frozen in dry ice by special delivery to the central laboratory. The procedure will be specified in the central laboratory manual.

Appendix 10: Procedure to be followed in case of suspicion of heart failure

All along the study, in case of occurrence of symptoms suggestive of heart failure (dyspnea, dyspnea with exertion, orthopnea, tachycardia, paroxysmal nocturnal dyspnea, unexplained cough, fatigue, oedemas) a **physical examination** should be performed to determine if there are signs of congestive heart failure (jugular venous distension, S_3 gallop, pulmonary rales…).

In case of suspicion of heart failure the investigator should prescribe as soon as possible and prior to any specific treatment, **NT-proBNP and serum creatinine sampling**. **If NT-proBNP ≤ 300 pg/mL heart failure can be ruled out** and the investigator may consider another diagnosis.

If NT-proBNP > 300 pg/mL, a full cardiological examination should be prescribed as soon as possible with:
- ECG: to assess changes from baseline.
- Doppler Echocardiography: to assess left ventricular filling pressure and pulmonary pressure.

The patient file should be transmitted as soon as possible to the **Adjudication Committee for assessment.**

If heart failure is confirmed:
- **Study treatment should be discontinued** (see paragraphe 9.6.1)
- Specific treatment for cardiac heart failure should be started under medical control. According to the level of emergency, the patient will be hospitalized or treated under outpatient medical care.
- **Patient will be controlled two weeks later** by his/her research doctor in order to evaluate his/her clinical medical state.

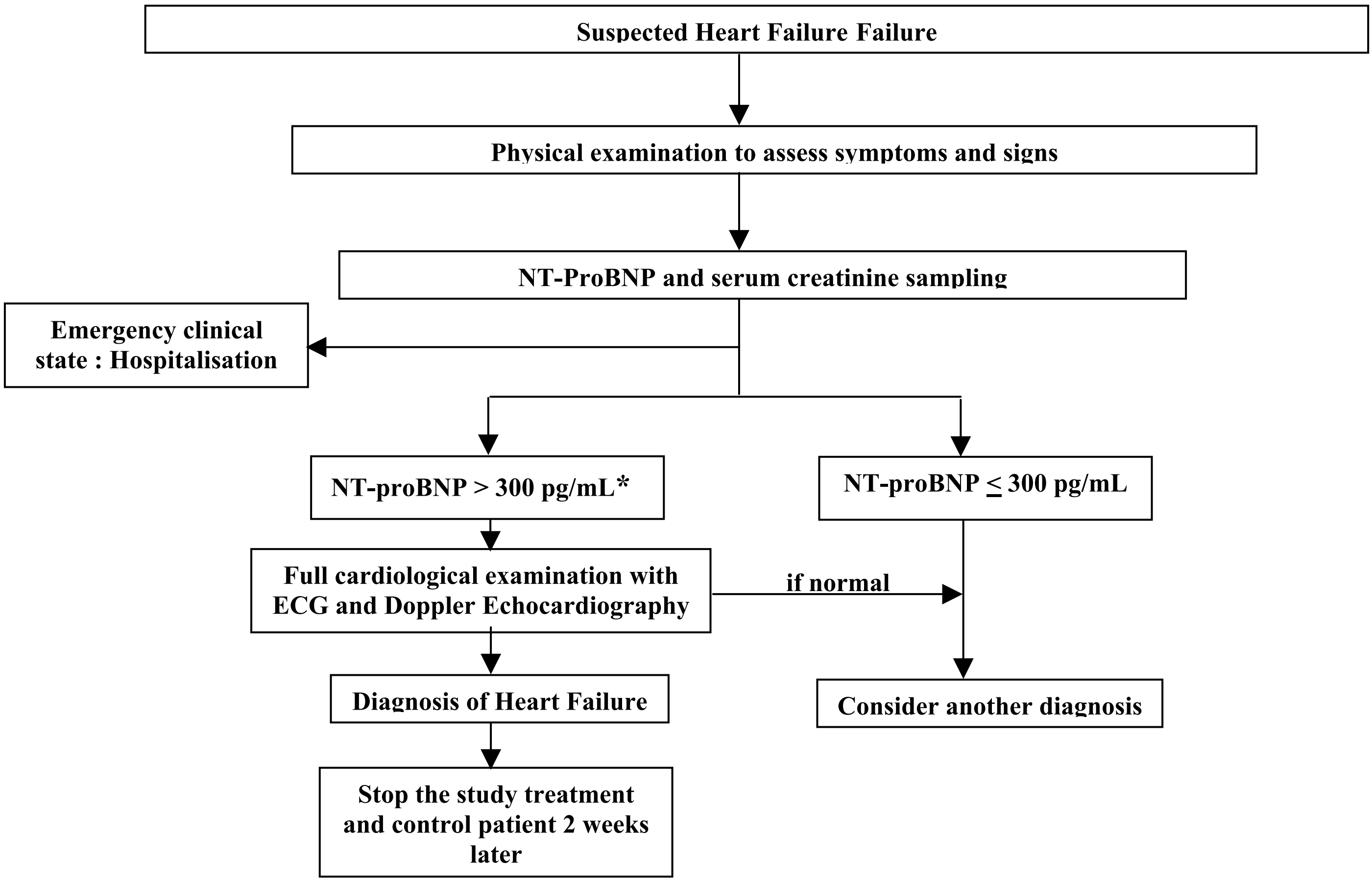

*All files with NT-proBNP > 300 pg/mL will be submitted to the Adjudication Committee

Appendix 11: Procedure to be followed in case of oedema

If oedema is present at selection visit, description should be performed and aetiology should be precised and reported in the part Medical History of the CRF.

All along the study, in case of occurrence of oedema the investigator should proceed as follow (record in CRF):
- **Describe** clinical symptoms.
- **Investigate** for signs of heart failure (eg, jugular venous distension, S_3 gallop, pulmonary rales).
- **Clarify** the aetiology of oedema (refer table bellow).

In case of oedema with hydrosodic retention, the investigator should perform as soon as possible and **prior to any specific treatment NT-proBNP and serum creatinine sampling.**

In case of oedema requiring emergency treatment, the patient should be hospitalized. Hospital report as well as any test performed during hospitalisation will be considered by the Adjudication Committee to analyse the case.

In case of oedema associated with heart failure see appendix 10: procedure to be followed in case of heart failure.

If oedema is moderate, treatment will only be started after biological tests performed and, if applicable, after cardiac examination by a cardiologist.

Description of oedema:

Clinical symptoms:

☐ Depressible ☐ Not depressible
☐ Bilateral ☐ Unilateral
☐ Painless ☐ Painful
☐ Gravity oedema
☐ Non inflammatory ☐ Inflammatory
☐ Increase of body weight ≥ 2kg within a week

Aetiology of oedema :

☐ Venous retention
☐ Lymphatic retention
☐ Renal aetiology
☐ Pre-menstrual oedema
☐ Calcium channel blockers treatment
☐ Vasodilators
☐ NSAID
☐ Hypothyroidism
☐ Other:

Clinical symptoms, if any, of heart failure:
☐ Jugular venous distension
☐ Pulmonary rales
☐ S3 gallop
☐ Hepatomegalia

☐ Heart failure suspected

In case of hydrosodic oedema (not including pre-menstrual oedema) tests should be performed :
- **NT-proBNP dosage**
- **Serum creatinine**

- If NT-proBNP is ≤ 300 pg/mL, you can consider another diagnostic than cardiac insufficiency.
- If NT-proBNP is > 300 pg/mL, patient should be prescribed **a full cardiac examination by a cardiologist.**

Appendix 12: Procedure to be followed in case of anaemia

An anaemia discovered during the study is diagnosed by the association of both:

- **Haemoglobin level < 12g/dL in men and < 11g/dL in women**

AND

- **Decrease of haemoglobin > 1.5g/dL in men and > 1g/dL in women**

The blood test will include erythrocytary indexes, haptoglobin and ferritin to clarify the aetiology of anaemia.

If anaemia is confirmed, the patient will stop his/her treatment (see paragraph 9.6.1), and usual investigations should be performed by the investigator in order to treat the patient. The investigator should also report the anaemia as adverse event (adverse event form should be completed).

Appendix 13: Adjudication Committee Procedure

- The following events, occurring during the course of the study, will be adjudicated:

→ All Serious Adverse Events
→ Adverse events that involve any cardiac or vascular disease (excluding pedal oedema with NT-proBNP ≤ 300 pg/mL)
→ Other cardiac event such as modification of cardiovascular concomitant treatment
→ Any clinically significant adverse event whose diagnosis is uncertain

- The **procedure for adjudication** will be as follows

→ As shortly as possible after the declaration of the event by the investigator in the CRF and under request of the Country Medical Reviewer, a specific file will be set up to provide the committee with all medical information related to the event. This file will include CRF corresponding pages (adverse event form, visit(s), concomitant treatments…), biological results, hospitalisation report or any other useful medical document (ECG, Doppler Echocardiography, cardiological report…).

→ The event's file will be anonymised by the CRO in charge of the monitoring; then it will be collected and transmitted to the Adjudication Committee by I.R.I.S. within the month following the onset of the event

→ Each of the three Adjudication Committee members will receive a copy of the complete adjudication file.

→ An Adjudication Committee meeting will take place under decision and coordination of the President of the Committee when a minimum of ten clinical cases will have been submitted by I.R.I.S.

→ Adjudication will be reported on a specific form (see hereafter) and transmitted to I.R.I.S. The investigator will be informed by I.R.I.S. of the Adjudication Committee opinion.

In case of event with suspected cardiac failure, diagnosis will be confirmed by the followings:

1. Clinical symptoms
2. NT-proBNP > 300 pg/mL
3. Doppler echocardiography
4. ECG

CL3-00780-148 Adjudication Committee Form

Meeting date : __________

Patient # |8 |__|__|__|__|__| **Country__________________________**

Date of onset : |____| |____| |____|
dd mm yyyy

Verbatim :

____

____

Documentation :

☐ |____| CRF

☐ |____| DCF

☐ |____| Adverse event initial form

☐ |____| Adverse event additional form

☐ |____| Hospital report

☐ |____| Cardiologist report :
date : ___________

☐ |____| Echocardiography

☐ |____| _______

☐ |____| _______

☐ |____| _______

OPINION:

Diagnosis: ________________________________

Possible relationship with medical history:

☐ yes

☐ no

☐ doubtful

Comments: _______________________________

____

☐ Impossible to conclude :
Missing data: _____________________________

____

Signatures :

G. Demureaux **H. Rousset** **A. Sérusclat**

Appendix 14: Chronic Heart Failure Classification (NYHA)

Class I	Patients without symptoms or only at levels that would produce symptoms in normal individuals
Class II	Patients with symptoms on ordinary exertion
Class III	Patients with symptoms on less than ordinary exertion
Class IV	Patients with symptoms at rest

The American Journal of Cardiology 1999, January 21

Appendix 15: Creatinine Clearance, Cockroft and Gault Formula

$$\text{Cl (mL/min)} = \frac{140 - \text{age (years) x weight (kg) x K}}{\text{serum creatinine } (\mu\text{mol/L})}$$

K = 1.25 for man and 1 for woman

Creatinine clearance will be automatically calculated by the central laboratory at W0.

Appendix 16: Protocol of Echocardiography Doppler

The following is a minimal list of the echocardiographic recordings that will be collected (3 consecutive cycles). All measurements will be made at the echo core laboratory.

Site echo accreditation :
The Echo Core Lab will request each participating site to submit a sample recording of all study related-views acquired in a patient that could fit the inclusion criteria. The recording will be reviewed at the core lab to assure that each site has appropriate understanding of the echo protocol and can satisfactorily acquire all necessary images and Doppler signals.

Image acquisition and measurements :

1- **M-mode of the left ventricle**
The following parameters will be measured :
   - left ventricular end-diastolic diameter (mm) (sweep speed of 50 mm/sec)
   - left ventricular end-systolic diameter (mm) (sweep speed of 50 mm/sec)
   - left ventricular fractional shortening (%)

2- **Parasternal long-axis of the left ventricle**
The following parameters will be measured :
   - left ventricular out flow tract diameter (mm)

3- **Apical 4-chamber view**
The following parameters will be measured :
   - left ventricular end-diastolic and end-systolic volumes using single plane Simpson's rule left ventricular end-systolic diameter (mm) (sweep speed of 50 mm/sec)
   - left ventricular end-diastolic and end-systolic lengths
   - left ventricular ejection fraction (%)

4- **Apical 4-chamber view with color-Doppler**
The following parameters will be measured :
   - mitral and tricuspid regurgitant jet areas
   - left atrial and right atrial areas

5- **Mitral flow velocity curve with pulsed Doppler (to be recorded at 100 mm/s)**
The following parameters will be measured :
   - E/A ratio
   - E wave deceleration time (ms)
   - Left ventricular filling time (ms)
   - Cycle length

6- **Pulmonary venous flow curve with pulsed Doppler (to be recorded at 100 mm/s)**
The following parameters will be measured :
   - S/D ratio

7- Left ventricular outflow velocity curve with pulsed Doppler (to be recorded at 100 mm/s)

The following parameters will be measured :

- Time velocity-integral of aortic flow (cm) to calculate the left ventricular stroke volume
- Left ventricular ejection time (ms)

8- Apical 2-chamber view

The following parameters will be measured :

- left ventricular end-diastolic and end-systolic volumes using single plane Simpson's rule left ventricular end-systolic diameter (mm) (sweep speed of 50 mm/sec)
- left ventricular end-diastolic and end-systolic lengths
- left ventricular ejection fraction (%)

9- Tricuspid flow velocity curve with continuous Doppler (to be recorded at 100 mm/s)

The following parameters will be measured :

- Pressure gradient (mmHg)

10- Subcostal views :

The IVC diameter will be visualized through several respiratory cycles for an estimate of right atrial pressure

In addition, mitral, aortic and tricuspid valvular thickness will be assessed from the different echo views.
Pay attention to obtain a good quality ECG.

<u>Non-inclusion criteria</u>

- Systolic Ejection Fraction < 40 %
- Aortic regurgitation: level mild or > mild
- Mitral regurgitation: level moderate or > moderate
- Tricuspid regurgitation: level moderate or > moderate
